
JOURNAL INFORMATION
==============================
NLM Title Abbreviation: J Orthop Traumatol
Journal ID: J Orthop Traumatol
NLM Journal ID: 101090931

Journal of Orthopaedics and Traumatology : Official Journal of the Italian Society of Orthopaedics and Traumatology

ISSN: 1590-9921
EISSN: 1590-9999
Publisher: Springer

ARTICLE INFORMATION
==============================
PMCID: PMC4417747
DOI: 10.1007/s10195-011-0149-8
Article ID: 149
Article version: 1
Subjects: Events

In-depth Oral Presentations, Oral Communications and Round Tables

Electronic publication date: 2011 Sep 2
Print publication date: 2011 Oct
Volume: 12
Issue: Suppl 1
First page: 23
Last page: 88
Copyright: © The Author(s) 2011
License: Open AccessThis article is distributed under the terms of the Creative Commons Attribution 2.0 International License (https://creativecommons.org/licenses/by/2.0), which permits unrestricted use, distribution, and reproduction in any medium, provided the original work is properly cited.
License URL: https://creativecommons.org/licenses/by/2.0/

Keywords: Anterior Cruciate Ligament, Anterior Cruciate Ligament Reconstruction, Oswestry Disability Index, Radial Head, Distal Radius Fracture
issue-copyright-statement: © The Author(s) 2011

==============================
J Orthopaed Traumatol (2011) 12 (Suppl 1):S23–S88
DOI 10.1007/s10195-011-0149-8

3 OCTOBER 2011

### In-depth Oral Presentations, Oral Communications and Round Tables

### IN-DEPTH ORAL PRESENTATIONS

### *AT01—BIOTECHNOLOGY IN THE FIELD OF TRAUMA 1*

#### The bioresorbable screws in the tibio-fibular syndesmosis injury

F. Simeone[1], M. Memminger[2], P. Simeone[3]

[1]Clinica Ortopedica e Traumatologica, IRCCS Policlinico San Matteo (Pavia, IT);
[2]Divisione Ortopedia e Traumatologia (Bolzano, IT);
[3]Poliambulatorio Centro Thuja (Piacenza, IT)

**Introduction** The bioresorbable osteosynthesis avoids the implant removal and give advantages (perhaps) in fracture healing. An interesting application is in the tibio-fibular syndesmosis injury because it avoids the hospitalization for screw removal.
**Materials and methods** 19 patients with type C fracture of Danis–Weber classification. We used a 3.5 mm resorbable screw made of phydroxyapatite/poly-L-Lactate, alone (8 patients) and in associations with fibular plate (11 patients). After the operation we didn't allow the patients to load on the affected limb with or without half plaster. After this period load was permitted without restriction. The results were clinically and radiographically evaluated after operation, at 5 weeks and at last follow-up (mean 20 months).
**Results** We didn't have any loss of syndesmosis reduction, with both clinical and radiological satisfactory results. We didn't need either other surgical approach or hospitalizations. We didn't have either inflammation reaction or osteolysis.
**Discussion** In our opinion a bioresorbable osteosynthesis must have high strength, biocompatibility, radiopacity, good bone bonding capability, resorbability with total bone replacement. From 1985 various authors (Rokkanen first) used resorbable syndesmosis screw made of poliglicolic acid. But there were tissue reactions (from 5.3 to 11.1%) and rapid strength loss, inflammation reactions and osteolysis. Thereafter polylactade screws were implanted in different formulations. The tissue reactions diminished, but there was interposition of fibrotic tissue between bone and implant and often there was partial replacement by adipose/fibrotic tissue with slow and nonlinear degradation process; furthermore the screws weren't radiopaque. We used a screw made of polilactic acid reinforced by unsyntered and uncalcified hydroxyapatite (u-HA) = Osteotrans. The bending mechanical strength of Osteotrans is 270 Mpa, the SS one is 280 Mpa, the cortical bone one is 210 Mpa. In Osteortrans screws the bending strength diminishes of 15% after 12 weeks, of 25% after 24 weeks, of 50% after 52 weeks. The SS elastic module is 200 Gpa, the titanium one is 100 Gpa, the Osteotrans one is 7.5 Gpa, near to the bone elastic module (1–20 Gpa): the stress shielding is really diminished. We obtained an excellent bone integration thanks to the surface hydroxyapatite which is responsible of directly bonds with surrounding normal bone without intervening of fibrotic tissues. Radiopacity is ensured by Hydroxiapatite.
**Conclusions** We think that the use of screws made of polilactide and hydroxyapatite is a good solution to avoid a surgical removal procedure with a good compliance and perhaps a lower infection rate.

#### Treatment of long bone nonunion using bone grafting, autologous bone marrow stromal cells and platelet gel

D. Dallari, N. Rani, N. Del Piccolo, C. Stagni, C. Carubbi, A. Giunti

Istituto Ortopedico Rizzoli (Bologna, IT)

**Introduction** Nonunions of long bones are a complication of fractures, often requiring a difficult treatment. They lose the possibility to consolidate because of formation of pathological callus. For this reason it is mandatory to ensure a stable synthesis, in combination with an appropriate biological stimulus, to reach consolidation.
The combined use of homologous bone, concentrated stromal cells and autologous platelet gel is a biological stimulus for bone regeneration, as PDGF, TGF-beta, IGF-1 and VEGF have been shown to positively influence the survival, differentiation and proliferation of bone cells. These effects are also active on stromal cells, with induction of differentiation in osteoblasts, osteocytes, and consequently in bone tissue.
**Materials and methods** From 2003 we treated 82 nonunions of long bones, divided as follows: 58 of lower limb (36 of femur and 22 of tibia) and 24 of the upper limb (12 of humerus, 5 of ulna and 7 of radio). In all patients we used homologous freeze-dried bone, concentrated stromal cells and autologous platelet gel. The average age was 42 years (26–70). In 38 cases we observed hypertrophic nonunion, whereas in the remaining 44 nonunion was atrophic. The type of treatment was also influenced by the characteristics and size of the lesion. In 9 cases we needed to use an intercalary homologous bone graft because of large defects.
**Results** The healing was observed after a median time of 3.7 (2–6) months for hypertrophic nonunion and after 4.5 (3–7) months for atrophic ones.
**Discussion** Several literature data suggest that growth factors can promote bone repair. Our preliminary results demonstrate the usefulness of platelet gel and autologous stromal cells concentrated in the treatment of pathological lesions such as nonunions of long bones.
**Conclusions** A good mechanical stability and a valid blood supply is essential for healing of fractures. The use of biological adjuvants, as reported in several studies in literature, represents a further stimulus to healing, making it particularly useful in difficult cases.

### The treatment of bone defects by homologous bone + DBX + MSC

A. Olmeda*, S. Pasquato, C. Gigante, E. Barina

Azienda Ospedaliera di Padova (Padua, IT)

**Introduction** The treatment of bone defects and pseudarthrosis has been grounded for several years on the use of autoplastic bone graft. Recently, human derived materials and genetics engineering products were introduced, broadening the therapeutic possibilities and reducing healing time. The most promising products are right now demineralized bone matrix (DBM), bone morphogenetic proteins (BMP) and marrow stem cells (MSC).
**Materials and methods** After literature reviews and following our experience, in the last years it has been chosen as common strategy to treat small and medium bone losses and pseudarthrosis with homoplastic graft + DBM + MSC + re-synthesis when needed. The homoplastic material has bee provided as massive graft (spilt) from Treviso's tissues bank. DBX comes from MTF laboratories. Re-synthesis has been performed through endomedullary nailing or plaques with angular stability LCP Syinthes. MSC where taken and concentrated with the Marrow-Stim technology. Up to now, 15 cases have been operated employing combined technique, among those 3 where bone cysts resistant to treatment, 2 acetabulum geodes, 10 pseudarthrosis (3 femur, 7 tibia) with re-synthesis in 6 cases. Infectious pseudarthrosis were excluded. All patients were controlled in our ambulatories until the end of the treatment.
**Results** Patients with bone cists were all healed within 2 months, pseudarthrosis of femur and tibia within 6 months. No adverse reactions were found, neither to the homologous material or the cellular manipulation. In 3/10 pseudarthrosis cases the bone callus, although clinical solid, showed some aspects of incomplete structuring. This report, pointed out in most of published cases, need to be controlled in the course of time, in order to confirm the consolidation reliability.
**Discussion** The sample presented is still limited and with a preliminary follow-up, but it allows verifying the efficacy of a combined treatment for bone losses and pseudarthrosis through modern technologies. It's confirmed that autoplastic bone is no longer necessary to reach satisfying results. Large randomized studies are needed in order to evaluate officacy of different products and to neutralize the statistical influenza or re-synthesis.
**Conclusions** Homoplastic bone, associated with DBX + MSC, has showed an effective potential for fast healing in different pathologies linked with the presence of a bone gap.

### Clinical results of peripheral nerve regeneration by collagen-based artificial nerve guides

A. Merolli*, L. Rocchi, R. De Vitis, L. Mingarelli, F. Catalano

Università Cattolica (Rome, IT)

**Introduction** Collagen-based artificial nerve-guides (Neuragen, Integra; NeuroMatrix, Stryker) were used in patients with below-the elbow nerve-gap-lesions up to 30 mm, as an alternative to autologous nerve graft or neurectomy.
**Materials and methods** In the first 15 operations we implanted 17 guides in 14 patients. We had a second-look in ten guides: five in digital nerves, two in median nerves, two in ulnar nerves and one in superficial radial nerve. The reasons for the second-look were to perform tenolysis in associated tendon injuries or revision of the site for other minor complaints.
**Results** In digital nerves, second-look was performed after a mean of 4 months (3–6). A nerve-regenerate was found inside all guides. Three out of five guides appeared only mildly degraded. Sensory recovery was effective in all patients and none experienced painful neuroma. In median nerves, second-look operations were performed after 9 months. In one patient, a nerve-regenerate 30 mm long was observed. A progressing sensory recovery was documented, however no motor recovery occurred. In a second case (a child of 5-years of age), a bulbous neuroma was found and just a tiny bundle of 25 mm was joining the original stumps. Sensory and motor recovery was nearly complete. Both guides appeared partially degraded. In ulnar nerves, second-look operations were performed after 9 months. In one case a nerve-regenerate of about 8 mm was observed, together with adequate sensory recovery and partial motor recovery, notably in a 70-year old patient. In the second case an empty guide was found; no signs of sensory or motor recovery were ever evidenced. Both guides appeared partially degraded. In radial superficial nerve, second-look operation was performed after 9 months. A nerve regenerate bundle joining a 25 mm gap was found embedded in the highly degraded remnants of the guide. An effective sensory recovery was observed and no painful neuroma was ever experienced.
**Discussion** The second-look operations gave us the peculiar opportunity to assess macroscopically what was occurring inside the implanted artificial nerve-guide and to correlate these findings with the clinical data available on functional recovery.
**Conclusions** An adequate sensory recovery occurred in all patients were a nerve-regenerate was found inside the guide. No painful neuroma was recorded in any patient.

### The principle of the biological chamber in the treatment of PSA and complex post-traumatic bone losses

G.M. Calori*, M. Bucci, P. Fadigati, E. Mazza, M. Colombo, C. Ripamonti

C.O.R., Istituto Ortopedico G. Pini (Milan, IT)

**Introduction** The treatment of nonunion (PSA) of long bones and bone defects remains a critical challenge in orthopaedics. Recently, it has been codified in the "Diamond Concept" which pointed out the different appliances, mechanical and biological underpinning the process of bone regeneration, distinguishing four basic elements: a stable biomechanical environment, cells, scaffolds and growth factors. This concept was further supplemented with a fifth element: the blood supply is also essential to the process of bone formation.
**Materials and methods** The following work aims to expose the innovative concept of "biological chamber" as part of tissue engineering and regeneration applied within bone in trauma. The aim of the study is to explain the evolution from the principle of "Diamond Concept" through the concept of "Pentagon" up to the "biological chamber" and give pointers on how to create such a delicate creature with biotechnology.
**Results** The chamber is a real biological reactor in which there are all the elements needed for tissue regeneration. It is created by performing a large en-bloc resection of the lesion with a good debridement. The environment created must be aseptic, viable, stable, mechanically sealed and selectively permeable. In this regard, there are many techniques to close the chamber: adequate mobilization of soft tissue coverage with free muscle flap, creating a Masquelet's membrane, application of biological agents such as homeostatic or collagen membranes, covering with bone whether it could be autologous, homologous or synthetic.
**Discussion** It has been demonstrated since the mid-90 s that some growth factors may act as potent stimulators of osteoblast proliferation in vitro and in vivo bone healing. It could be very useful in facilitating the healing process if applied properly in the lesion. Mesenchymal stem cells harvested from the iliac wing and concentrated by centrifugation or filtration appear to be also effective for bone regeneration, especially if associated with growth factors and a good osteoconductive scaffold.
**Conclusions** These biotechnologies are thus a powerful and valuable tools, several international studies underline this effectiveness and safety. Strict indications and a correct usage are, however, a priority in order to prevent waste or bad uses.

### *AT02—BIOTECHNOLOGY IN THE FIELD OF TRAUMA 2*

### Mesenchimal stem cells for treatment of cartilage defects in sheeps: biomechanical tests ex vivo

A. Manunta*, F. Pisanu, F. Marras, L. Saturno, A. Melis

AOU Sassari, Clinica Ortopedica (Sassari, IT)

**Introduction** Stem cells are self renewing, unspecialized cells that can give rise to multiple cell types of all tissue of the body. They can be derived from the embryo, foetus and adult. The aim of our study was to test mechanical properties of the stem cells from bone marrow or from umbilical cord, after seeded on fibrin glue and grow and form cartilage in chondral defects when used for repair the lesion.
**Materials and methods** Pluripotent cells derived from inner mass cell of embryos (ES) at the first stages of development (blastocysts) and mesenchymal stem cells (MSC) isolated from bone marrows aspirates and umbilical cord. The cells were isolated from feeder layer and seeded on fibrin glue before the transfer. We proceeded with aspiration of cells in fibrinogen and successively addition of trombin at time to transplantation. In 6 sheeps, in correspondence of medial femoral condyle on both knees, we performed a hole 5 mm diameter and 3 mm in depth with anatomic drill be careful to avoid bleeding, obtaining an chondral full thickness lesion. The creation of full-thickness defects was performed in such a manner as to render it impermeable to blood-borne cells and signalling substances emanating from the subchondral bone-tissue spaces. After this time, into the left knee 20 μl of fibrinogeno + 20 μl of of trombina were inserted whether on right knee 20 μl of fibrinogeno + 20 μl of trombina with 20 μl of pellet into the hole. The new tissue obtained was tested using the ICRS classification, and analysed biomechanically by the Artscan 200 series. We performed besides immunohistochemical evaluation of cartilage to check collagen type I.
**Results** Specific attention was directed toward the filling of the defect useful for determination of biomechanical behaviour in the reparative tissue but this positive labeling was not found in every specimens from defects that underwent to stem cells cartilage procedures. 12 months after transplantation, biomechanical evaluation showed that the reparative tissue was more hard in the group treated with mesenchimal cells than embrionic stem cell or control group.
**Conclusions** Although ES are promising for cartilage repair, being rapidly proliferating are serious limitations of their use in therapy, while numerous mesenchimal cells could improve the mechanical property of cartilage when implanted in a condral defect in which micoenviroment is suitable for the survival and differentiation of MSC.

### Biological resurfacing of unicompartmental patello-femoral, tibio-femoral and ankle OA by ACT hyalograft-C with SET TECHNIQUE

S. Zanasi*

Villa Erbosa Hospital, GSD e Domus Medica (Bologna, IT and San Marino, RSM)

**Introduction** In patients younger than 50 years, affected by unicompartmental osteoarthritis of the knee or ankle, we have obtained very promising results by biological coating of both kissed joint surfaces resorting to ACT.
**Materials and methods** From February 2004 to February 2009 we treated by ACT SET TECHNIQUE 28 patients (13 m/15f) affected by osteoarthritis Ahlback stage III/IV for medial (8) and lateral (2) unicompartmental femuro-tibial OA, patella-femoral joint OA (14), and 4 unshouldered kissed ankle large defects. The average age was 31 years (19–43 years). All patients were followed for a minimum follow-up of 24 months (24–84 months). The clinical evaluation was performed using the ICRS-IKDC protocol; the evaluation of the cartilage was performed by a 1.5T MRI imaging and processing was performed by MOCART scoring system. The EuroQol EQ-5D was used to assess the quality of life of patients.
**Results** The ICRS-IKDC scoring documented a statistically significant improvement in clinical objective and subjective data ($p = 0.0001$). The EuroQol EQ-5D index is significantly improved compared to baseline in all patients. NMR imaging at 24 ms f.up revealed a complete filling of the defect in 73%, a complete integration of the border area with the adjacent cartilage in 83%. It has not been demonstrated at f.up progression of preoperative CXR. The 5–24 ms second-look surgery showed good coverage (>90%) and the integration of the grafts. Histology documented hyaline fibrous tissue with a high content of collagen type II and proteoglycans.
**Conclusions** These results suggest that autologous chondrocyte implantation even in unicompartmental osteoarthritis is an effective treatment for a biological coating that provides at least a medium-term data, remission of pain and swelling, with good functional recovery and the ability to resume previous work and sporting activity.

### Bioplasty for vertebral fractures: a pre-clinical study on goats using autologous modified skin fibroblasts

E. Pola*, G. Logroscino, L.A. Nasto, G. Di Giacomo, D. Colangelo, B. Rossi, M. Genitiempo, C.A. Logroscino

Università Cattolica del Sacro Cuore, Policlinico "A. Gemelli" (Rome, IT)

**Introduction** Osteoporosis is the most common metabolic bone disease and kyphoplasty and vertebroplasty are widely accepted as mini-invasive treatment options for painful vertebral osteoporotic fractures in the elderly. The aim of the study is to describe a minimally invasive percutaneous intrasomatic ex vivo gene therapy approach using autologous modified skin fibroblasts to treat thoracolumbar fractures and anterior column bone defects in a goat model.
**Materials and methods** Ten wild-type female goats were used. Primary dermal fibroblast cultures were established using a 0.5-cm-diameter punch skin biopsy. Subconfluent dermal fibroblasts were transduced with adenoviral-carrier BMP-2 gene and absorbed on a scaffold of porous hydroxyapatite and collagen. After 48-h from viral infection, the mixture of scaffold + autologous dermal fibroblasts transduced with BMP-2 was injected in the 4th lumbar vertebra of each goat by means of a percutaneous kyphoplasty-like procedure. The mixture of scaffold + non-trasduced cells injected in the 3rd lumbar vertebra and the scaffold alone injected in the 6th lumbar vertebra of each goat were both considered as negative controls. All the defects were obtained by mean of a curette before the injections. Animals were sacrificed 1 and 3 months after the operation and all specimens were studied through CT scan and histological sections.
**Results** At 1 month after surgery, vertebrae treated with scaffold + BMP-2 transduced skin fibroblasts showed an advanced bone healing with complete filling of the vertebral body defect after 3 months. All the controls showed a delayed healing of the bone defect at the same time points.
**Discussion** The purpose of this experimental approach is to promote consolidation of vertebral fractures and anterior column bone defects through a biological way. Instead of standard vertebral augmentation techniques using PMMA, which merely fill vertebral defects through a substitution of trabecular bone, the main advantage of this mini-invasive procedure is to strengthen bone healing and to facilitate a more biological tissue remodeling.
**Conclusions** Our findings suggest the feasibility of using a minimally invasive percutaneous approach to deliver gene therapy engineered cells in the vertebral body. This procedure combines the advantages of mini-invasive surgical techniques with the biological effects of gene therapy on bone healing, representing a potentially new treatment for stress vertebral fractures.

### Surgical treatment of traumatic fractures of the thoracic-lumbar rachis: use of synthetic bone during somatic transpedicular reconstruction

G.A. La Maida*, A. Acerbi, L.S. Giarratana, G. Mineo, B. Misaggi

Istituto Ortopedico G. Pini (Milan, IT)

**Introduction** Since 2000 to nowadays we have performed more than 150 vertebral segmentary surgical stabilization procedures using a posterior access in Patients affected by thoracic-lumbar spinal fractures. This study describes the results obtained through the employment of this technique in a sample of 70 patients among this group (5 years minimum follow-up).
**Materials and methods** The sample considered in this work is composed by 38 men and 32 women with a mean age of 53 years (range 18–84). Fifteen patients underwent, in addition to a standard transpedicular stabilization treatment, a vertebral augmentation procedure through the employment of synthetic bone, in order to try to support the anterior spine using a unique surgical posterior access. Twenty patients did not undergo any arthrodesis procedure in addition to the instrumental stabilization, while 50 Patients underwent also arthrodesis using either autologous bone (13/70), either synthetic bone (13/70) or mixed autologous-syntethic bone (24/70).
**Results** Excellent results were obtained in almost all cases, with a particular relevance of traumatic fractures treated using short vertebral instrumentation and somatic reconstruction through the transpedicular employment of synthetic bone.
**Discussion** The choice of the approach that has to be used to treat burst fractures (A3) of the thoracic-lumbar spine is still under discussion. Somatic reconstruction through a posterior access gives the possibility to avoid a successive surgical treatment through an anterior-lateral access, effectively reducing the risk of possible complications.
**Conclusions** In summary, observing the satisfactory clinical and radiographic results, we can affirm that the use of a segmentary instrumentation through a transpedicular access allows the treatment of most of the traumatic and nontraumatic vertebral fractures. Besides, the combined employment of a procedure of vertebral augmentation using synthetic bone allows an optimal restoration of the anterior spine.

### PSA with critical bone loss in the forearm: role of biotechnology

G.M. Calori*, M. Bucci, P. Fadigati, E. Mazza, M. Colombo, C. Ripamonti

Istituto Ortopedico G. Pini (Milan, IT)

**Introduction** NU and bone defects of the forearm are critical complications in the trauma of the upper arm. These cases are characterized by severe loss of substance that is replaced by abnormal necrotic tissue which should be discarded. Breast reconstruction with vascularized fibular autograft is an available therapeutic choice but not free of complications. For several years we have followed this surgical protocol: radical resection of pathological tissue (en bloc), until you get to healthy bone and bleeding, opening the diaphyseal canal, osteoconductive material implant (scaffold enriched with autologous mesenchymal cells) and osteoinductive (growth factors), mechanical stabilization achieved with fixed-angle plate supported by allogenic fibular splint. The purpose of this procedure is to create a "biological chamber" that should be considered as a real biological reactor in which all necessary elements for bone regeneration are present. The purpose of this study is to validate the effectiveness of this proposed surgical technique in combination or not with the use of biotechnology in polytherapy and analyze the influence on the spectrum of stability of the allogenic graft implant.
**Materials and methods** We analyzed 33 diaphyseal PSA of the forearm, 15 patients out of these were treated without biotechnology (Group A) and 18 treated with biotechnology (group B), in 8 of the latter was not used while in the remaining peroneal splint 10 has been performed the allograft implantation. The results were analyzed by a clinical and radiographic evaluation.
**Results** The cure rates were 73.3% in group A for both clinical and radiographic point of views versus 83.3% in group B. 26.7% of group A required further surgery versus 16.7% of the second group.
**Discussion** All (3/18) the failures of group B were treated with allogeneic graft without biotechnology, and they all needed a re-operation due to mechanical failure of synthesis. This underscores the importance of the allogeneic implantation to restore the range of stability of the system.
**Conclusions** Based on our results we can conclude in favour of the use of biotechnology (rh-BMP-7 + xeno + allogeneic implant and MSC) in the treatment of bone defects of the PSA and critics bone loss of the forearm. The operation is also well tolerated by patients because, due to the good stability of the system, the limb may be released early.

### Severe bone defects treated with pluripotent mesenchymal autologous cells harvested from iliac crest bone marrow

L. Murena[1], E. Vulcano*[1], D.A. Falvo[1], A. Baj[2], A. Toniolo[2], P. Cherubino[1]

[1]Clinica Ortopedica Traumatologica (Varese, IT);
[2]Dipartimento di Microbiologia (Varese, IT)

**Introduction** At present time, several studies investigating the role of stem cells in the treatment of different pathologies have been reported in the literature. Nonetheless, the role of pluripotent mesenchymal autologous cells for treating bone defects is still not clear. The aim of the present study is to evaluate to usefulness of autologous bone marrow nucleated cell concentrate harvested from the iliac crest in the treatment of severe bone defects non-respondent to traditional therapies.
**Materials and methods** Between October 2008 and March 2010, 14 patients underwent surgery for severe bone defects. Mean age of patients was 58.4 years (range 18–84 years). All patients had been previously treated with traditional surgical procedures without success. Patients were affected by any of the following defects: periprosthetic osteolysis in acetabular cup mobilisation, femur non union, humerus non union, complex exposed leg fracture and proximal humerus cyst. Lower limb bone defects were greater than 5 $cm^3$, while upper limb defects were greater than 2 $cm^3$. Patients affected by non unions were included in the study only after 2 months from last surgery.
Mean bone defect size was 53 $cm^3$ (range 33–103 $cm^3$). Mean number of previous surgeries was 2.57 (range 0–15 surgical interventions). Bone defects were treated with bone allograft enriched with bone marrow nucleated cell concentrate harvested from the iliac crest. Clinical and X-ray follow-up was performed at 1, 3, 6 and 12 months after surgery.
**Results** To date, average follow-up is 8.4 months (range 3–17 months). In 12 of 14 patients bone graft integration with both clinical and x-ray healing of the bone defect was observed at mean 5 months (range 2–12 months). Two patients did not heal. No post-operative complications were observed in any patient.
**Conclusions** All presented cases demonstrated that allogenic bone graft enriched with bone marrow nucleated cell concentrate harvested from the iliac crest facilitates healing of large bone defects in a heterogenous group of patients. The 2 patients who did not heal after being submitted to the presented technique were complex cases ab initio, making it difficult to understand whether the procedure has in any way affected the progression of the bone defect. In all cases, the technique proved to be safe and easy to perform, without significantly increasing surgical time. Furthermore, if compared to other techniques and drugs available for the treatment of bone lesions, the presented technique has lower costs. In addition, with respect to autologous bone graft from iliac crest, the procedure described in this study is associated to a lower risk of complications at the donor site. The success of the above mentioned technique also derives from the contribution that the latter has given to the healing of severe bone defects that had not responded to multiple previous surgical interventions in almost all cases.

### Diaphyseal and metaphyseal nonunions treated with bone morphogenetic protein BMP-7 and autologous bone-marrow concentrate

N. Mondanelli*, P. De Biase, P. Cuomo, D.A. Campanacci, G. Beltrami, G. Scoccianti, M. Ippolito, D. Matera, L. Ciampalini, R. Capanna

A.O.U. Careggi (Florence, IT)

**Introduction** Although a better understanding of and improved surgical techniques for the treatment of fractures, progression to nonunion is a possible occurrence whose incidence is rather increasing due to the raise of high-energy trauma (motor-vehicle and working accidents, open fractures, bone loss and/or comminution, soft tissue impairment), resulting in reduced biological capabilities.
**Materials and methods** From May 2002 to December 2008, we treated 74 cases of aseptic stabilized nonunion in the meta-diaphyseal region of a long bones of the limbs. Eighteen cases involved the upper limb (1 clavicle, 12 humeri, 5 ulnae, 1 radius) and 56 cases involved the lower limb (27 femora, 29 tibiae). We modified the osteosynthesis in 14 and 53 cases in the upper and lower limb, respectively. In the upper limb we always used a locking plate system, while in the lower limb an intramedullary nail was used in 40 cases, plate and screws in 9 cases and a cast immobilization in one case.
**Results** In 6 cases, nonunion was treated with percutaneous infiltration of bone marrow concentrate (BMC), resulting in bone healing in 4 cases (67%) to an average interval of 6 months. In 17 cases, nonunion was treated with a composite graft made of homologous bone chips, BMC and platelet-rich plasma; 87.5% of these patients healed at an average time of 5 months. In the remaining 57 cases, we employed the recombinant human bone morphogenetic protein 7 (rhBMP-7; Osigraft, Stryker, Limerick, Ireland) alone (44 cases) or in combination with BMC and homologous bone graft (13 cases); 89.4% of these patients healed at an average time of 4 months. The use of Osigraft increased over the years and it replaced other growth factors, because of its high success rate and simple surgical technique.
**Discussion** The addition of other growth factors or autologous bone graft did not show superior results, and therefore our recommendation is to use autologous grafts only in massive defects (>4 cm), while in minor defects an allograft may be used to obtain mechanical stability and as a scaffold for osteogenic proteins, with similar results.
**Conclusions** The use of BMP-7 is a simple and effective method to treat simple and difficult nonunions, provided you obtain mechanical stability. On the other hand, Osigraft is very expensive but is likely to lower; in addition, studies have shown the total economic benefit of this therapeutic approach, and patient satisfaction is definitely superior to other conventional approaches.

### *AT03—INFECTIOUS DISEASES*

### Local antibiotic infiltration therapy in chronic osteoarticular infection of the hip: personal experience

M. Ciotti[1], L. Fisichella[2], A. Cambria[2], S. Mauro[2], C. Falcone*[1], M.A. Rosa[2]

[1]Istituto Codivilla-Putti (Cortina d'Ampezzo-Belluno, IT);
[2]Dipartimento Specialità Chirurgiche, Sezione Ortopedia e Traumatologia, Scuola di Specializzazione in Ortopedia e Traumatologia, Università degli Studi di Messina (Messina, IT)

**Introduction** The bone and joint infections are a series of complex debilitating diseases, with plurispecialistic expertise, then they need a multidisciplinary approach. We present the results about the technique of local antibiotics infiltration applied in a large series of patients in Codivilla Putti Institute at Cortina d'Ampezzo. This technique was successfully used in the treatment of osteoarticular infectious diseases such as spondylodiscitis, septic arthritis, and infected arthroplasty.
**Materials and methods** From January 2008 till December 2009, 112 patients (73 women and 39 men) with a mean age of 64 years (range 17–90 years) received three infiltrative cycles in the infection site with targeted antibiotics. The pathogens identified were: *S. epiderm.* (49 cases), *S. aureus* (34 cases), *Pseudomonas aeruginosa* (16 cases), and other pathogens in 13 cases.
**Results** By completing a special "form" of treatment evaluation the patients participate actively to this study. At the end of this study the following results were obtained: excellent in 35, good in 48, sufficient in 11, inadequate in 18 cases.

**Discussion** The aim of this study is to demonstrate, with a follow-up period of 2 years, the validity and effectiveness of the method used. In addition this kind of treatment can be used in the definitive and/or transition treatment of patients with septic disease. We extensively studied the patients with septic arthritis and periprosthetic infection, and evaluated the benefits of such a method using it either in patients with poor general health or in patients who did not accept the advised surgical treatment. This infiltration technique can also be used to prepare the infected site for a possible re-artroplasty. The degree of patient satisfaction, clinical data and achievements were encouraging.
**Conclusions** Two-year follow-up results are the expression of good clinical evaluations; however they require confirmation by laboratory studies, aimed to explore the biological effectiveness of this treatment.

### Use of instrumentation and vertebral body reconstruction in surgical treatment of cervical spondylodiscitis

V. Denaro*, A. Di Martino, A. Lanotte, S.A. Barnaba, P. Nicola

Cattedra di Ortopedia, Università Campus Bio-Medico di Roma (Rome, IT)

**Introduction** It is under discussion whether it is possible to use metallic instrumentation when dealing with bone defect reconstruction and stabilization in patients with diagnosis of spondylodiscitis. The aim of the following work is to report an analysis of our personal casuistics in the use of instrumentation and cage reconstruction in patients affected by spondylodiscitis localized at the cervical spine.
**Materials and methods** A retrospective analysis of 84 patients affected by spondylodiscitis who were surgically treated, with a follow-up of 2–12 years. All the patients underwent decompression and debridement, reconstruction of the loss of substance with metallic means of synthesis and bone transplant. The anterior stabilization was performed by the use of a titanium mesh cage, while the posterior fixation required the application of lateral mass screw based systems, or through the use of Songer wires to stabilize the C1–C2 segment. All patients underwent post-treatment specific antibiotic therapy.
**Results** At the cervical level, we treated 12 patients, localized respectively at the axial ($n = 3$) or subaxial level ($n = 9$). The most frequent pathogens were *Mycobacterium tuberculosis* ($n = 6$), *Staphylococcus aureus* ($n = 3$), *Staphylococcus epidermidis* ($n = 1$), Haemophilus ($n = 1$), *Brucella* ($n = 3$). The follow-up controls showed fusion at the CT scan and stabilization at the dynamic lateral X-rays. No or distant site recurrence was observed, nor the mobilization of the instrumentation.
**Discussion** A considerable increase in spinal infections has recently occurred due to new migratory fluxes, recent increase of immunodepressed patients, and the increase in surgical procedures with eventual iatrogenic infection. The treatment of spondylodiscitis comprehends the combined use of spinal bracing and a conservative approach based on the antibiotic therapy targeted over cultures. Surgery is indicated in those patients with antibiotic resistant bacteria, and in case of neurological deficits or significant deformity on the sagittal plane. Surgery consists of a wide decompression of the neural structures, the curettage of the infected material, and the reconstruction of the bony loss. Our experience supports the use of metal instrumentation and titanium based cages in patients with spondylodiscitis at the cervical spine.
**Conclusions** At the cervical spine the aggressive and premature surgery is of great importance for the prevention of heavy complications. The wide debridement and the bone loss reconstruction, together with a stable fusion allows to obtain good results in terms of local stability and neurological recovery. In our experience, the use of instrumentation, together with a prolonged antibiotic therapy, is associated with good long-term results without local relapse.

### Two-stage procedure with subsequent spacer exchange for septic revision

D. Bruni*, F. Iacono, M. Lo Presti, G. Raspugli, S. Zaffagnini, M. Marcacci

Istituto Ortopedico Rizzoli (Bologna, IT)

**Introduction** Once the diagnosis of periprosthetic knee infection is confirmed, the critical step in a two-stage exchange arthroplasty is to determine the timing of reimplantation.
**Materials and methods** We performed a retrospective clinical and radiographic evaluation of 87 patients treated with subsequent static antibiotic-loaded cement spacer exchange for a suspected PKI at a mean follow-up of 7 years.
**Results** We had a total amount of 10 recurrent infections after RTKR (11%). In the remaining 77 cases (89%) our procedure of two-stage RTKR with subsequent cement spacer exchange allowed us to eradicate the PKI and patients had no evidence of re-infection at a mean follow-up of 7 years. None of the patients had severe or fatal adverse events related to treatment and all patients were alive at observation point. Results from microbiological culture of pre-operative Joint Aspiration were negative in 68 cases (78%) and positive in 19 cases (22%). Results from microbiological culture of intraoperative biopsies obtained from samples of synovial fluid and periprosthetic tissue were negative in 37 cases (43%) and positive in 50 cases (58%). In our group, we found a significant correlation between CRP abnormal values and positive Leukoscan ($p = 0.0493$).
**Discussion** Intra-operative cultures from samples of synovial fluid and periprosthetic tissue is mandatory and can provide additional informations on the ideal specific antibiotic therapy choice for subsequent ALBC spacer exchange, but it actually has low sensibility and poor negative predictive value.
**Conclusions** We recommend the use of a pre-operative Leukoscan to determine the correct timing of reimplantation.

### Septic shoulder prosthesis: surgical treatment and results of a consecutive series of 20 cases

C.L. Romanò*[1], L. Gala[1], N. Logoluso[1], E. Meani[2]

[1]Centro di Chirurgia Ricostruttiva e delle Infezioni Osteo-Articolari, I.R.C.C.S. Ortopedico Galeazzi (Milan, IT);
[2]Centro delle Complicanze Ortopediche Settiche (C.O.S.), Istituto Ortopedico G. Pini (Milan, IT)

**Introduction** Although there are numerous works on the management of prosthetic hip and knee infection, yet little information is available as to regard how to treat infections of septic shoulder arthroplasty.
**Materials and methods** Between 1999 and 2009, 20 patients were diagnosed to be suffering from infection of the shoulder in two orthopedic centers that participated in this study. The infection was classified as delayed and late in 9 in 11 patients. All patients underwent surgical debridement of infected tissue and implantation of an antibiotic-loaded cement spacer. Culture test showed in 10 cases, the presence of staphylococci, two gram-negative bacteria and two Propionibacterium acnes, while in six other cases, the culture tests were negative, despite clinical signs of infection. All patients underwent surgical treatment and systemic antibiotic administration of two antibiotics for 4–6 weeks after surgery.

**Results** Among patients treated with spacer, 4 received a reverse prosthesis, while 16 retained the spacer to stay. Eradication of infection was achieved in 19 out of 20 patients (95%). Main complications included two humerus fractures and one spacer dislocation, treated conservatively. The subjective assessment of pain scale decreased from an average of 6.7 to a mean of 1.7, with five patients reporting pain of 0. The function of the shoulder was generally quite low, with a mean Constant shoulder score of 16 pre-operatively and 53 at the last follow-up. However, patient satisfaction was excellent or good in 16 patients and moderate in the remaining four.

**Discussion** The literature is particularly inadequate as regards the results of continuous series of patients with septic shoulder arthroplasty. Treatment by combined use of antibiotic spacer and prolonged antibiotic therapy was effective in our experience, the functional results are generally quite low, but approximately three-quarters of patients report a good level of satisfaction, while a high rate of eradication of infection and a good pain relief can be obtained in the majority of cases.

**Conclusions** Contrary to what is observed in the hip and knee, the antibiotic-loaded cement spacer can be maintained for long periods of time in the shoulder, with adequate clinical and radiographic follow-up. In selected cases a reverse prosthesis may be safely performed.

### Modular prosthesis with a silver coating for periarticular reconstruction in septic prosthetic and post-traumatic failures

R. Capanna*, N. Mondanelli, D.A. Campanacci, L. Ciampalini, G. Beltrami, P. De Biase, G. Scoccianti, P. Cuomo, M. Ippolito, D. Matera

A.O.U. Careggi (Florence, IT)

**Introduction** Infection in orthopedic surgery is a dreadful complication. Patients are often subjected to several surgeries with prolonged antibiotic treatment, and the risk of persistent infection and debilitating functional outcome is high. Often, a massive bone defect coexists, linked to the need to perform extensive debridement to remove necrotic or infected bone. The antimicrobial activity of silver ion has been known since ancient times (silver vases and cisterns for drinking water) and in recent years has been revived in everyday life (toothbrushes, underwear) as well as in medicine (wound dressings).

**Materials and methods** Recently, we developed an evolution of the modular prosthesis MegasystemC (Waldemar Link, Hamburg, Germany) with a silver coating (PorAg) and, at our Centre, from June 2010 to January 2011 were operated on 7 patients with a septic arthroplasty (3 cases, 1 hip and 2 knees) or a septic meta-epiphyseal post-traumatic deformity or nonunion (4 cases, 1 proximal and 3 distal femora). One patient with subtrochanteric nonunion was subjected to only 1 surgery before resection and modular silver-coating prosthesis, while in all other cases the number of previous surgeries ranged from 3 to 8. In 5 cases the infection resolved, while in 2 cases the infection was persistent (1 knee arthrodesis prosthesis as a results of septic knee megaprosthesis and the subtrochanteric nonunion) and it was decided to revise them one-stage.

**Results** In 5 cases the reconstruction was performed with a articulating prosthetic joint (2 proximal femur and 3 knee megaprosthesis of the distal femur) and in 2 cases with a knee arthrodesis prosthesis. Monitoring of inflammatory markers (ESR, C-reactive protein, fibrinogen) showed resolution of the infection in all cases. From the clinical point of view, all patients were satisfied with surgery. Radiographically, there are no signs of loosening or periprosthetic bone resorption.

**Conclusions** In conclusion, the preliminary results of such a limited group of patients are encouraging and demonstrate that the use of silver coating prosthesis may be indicated in the reconstructions of periarticular loss of substance in septic failures, making single-stage revision surgery safer.

### *AT04—TUMORS AND METABOLIC DISEASES*

### The evolution in the treatment of "solitary juvenile bone cysts" of the meta-epiphysis of the proximal humerus and femur: considerations on the use of growth factors from autologous bone marrow

F. De Meo*[1], G. Maccauro[2], F. Ciccolo[2], M.A. Rosa[1]

[1]Dipartimento di Specialità Chirurgiche, Sezione di Ortopedia e Traumatologia, Università degli Studi di Messina (Messina, IT); [2]Dipartimento di Scienze Ortopediche e Traumatologiche, Università Cattolica (Rome, IT)

**Introduction** Treatment of juvenile bone cysts has evolved over the past decades. It has undergone significant changes, making it less invasive for the patient. Over the last years the introduction of growth factors obtained by autologous bone marrow aspirate gave a further contribution to the treatment of these lesions. This practice may be burdened with considerable complications for patient age and proximity to the growth cartilage.

**Materials and methods** From 1980 to 2010, 102 patients (59 male and 43 female), aged between 8 and 25 years, who had a simple bone cyst in the proximal meta-epiphysis of the humerus and femur came to our observation. The treatment was represented in 90% of cases (91 cases) from 3 to 6 infiltrations of methylprednisolone (one after the other 4 consecutive months) with Jamshidi needle; in the remaining unhealed 10% (11 cases), cortisone infiltration was replaced by bone marrow aspirate.

**Results** 91 treated patients out of 102 underwent a complete healing after infiltration with cortisone; the remaining 11 did not respond to infiltrative cortisone treatment. These non-responder patients underwent bone marrow aspirate and infiltration of growth factors. This technique was repeated up to three times over 18 months, and in all cases there was a total remission of the lesion.

**Discussion** The literature reports variable rates of healing after pathologic fracture in patients with bone cysts, from 15% (Boseker 1968) to 80% (Neer 1973). In addition, treatment with curettage and bone grafting appears to be invasive and burdened by a recurrence (variable rates from 33% to 40% [Neer 1966, Campanacci 1986]). Even the subtotal resection, despite the aggressiveness of treatment, has a recurrence up to 8% (Fahey 1977). Campanacci in 1986 points out a lack of response in only 10% of cases after intracystic infiltration of methylprednisolone acetate. The treatment proposed by Scaglietti in 1979, is characterized by its a traumatic technique of infiltrations (by Jamshidi needle) and the very high percentage of complete and/or incomplete healing. Therapy with bone marrow infiltration can be a weapon to use exclusively with patients unresponding to cortisone infiltration.

**Conclusions** After reviewing the literature and analyzing our series, we conclude that the technique proposed by Scaglietti represents the gold standard. Finally bone marrow infiltration must be performed in selected cases, not-responder to cortisone infiltration, and especially with particular and dedicated techniques.

### Vascularized fibular reconstruction after bone tumor resections of the femur

D.A. Campanacci*[1], D. Matera[1], F. Frenos[1], G. Caff[1], G. Beltrami[1], G. Scoccianti[1], L. Del Croix[2], M. Innocenti[2], R. Capanna[1]

[1]S.O.D. di Ortopedia Oncologica e Ricostruttiva, Azienda Ospedaliera Universitaria Careggi (Florence, IT);
[2]S.O.D. di Microchirurgia, Azienda Ospedaliera Universitaria Careggi (Florence, IT)

**Introduction** Biological reconstruction of the femur with bone grafts is considered an alternative to prosthetic replacement after intercalary resections for bone tumors. The objective of the present study was to review our series of vascularized fibular grafts (VFG) reconstructions of the femur evaluating morbidity and long term functional outcome.
**Materials and methods** Thirthy-seven patients (20 M, 17 F) were treated at authors' Institution with vascularised fibular graft (VFG) after resection of a malignant bone tumor in 35 cases and an aggressive benign lesion in 2 cases. An intercalary reconstruction was performed in 34 and a knee arthrodesis in 3 cases. In intercalary reconstructions, VFG was associated with a massive allograft in 29 cases, with autologous bone chips in 2 cases (1 with cortical struts), with cortical struts in 1 case and VFG was used alone in 1 case. In 1 patient, the resected femur was autoclaved and reimplanted with VFG. In knee arthrodesis, VFG was used with allogrfat in 1 case and it was associated to a free autologous fibula and bone chips from the iliac crest in 2 cases.
**Results** Fourteen major complications were observed. A fracture occurred in 10 cases, healed spontaneously in 2 and after surgical revision in 7 cases. In one patient the VFG was removed and replaced by a new VFG + allograft. A pseudoarthrosis was observed in 3 patients, 2 healed after surgical revision. One patient developed an artero-venous fistula requiring surgical repair. No deep infection was observed. At donor site, two patients presented a valgus ankle deformity, resolved after tibiofibular synostosis in one case, and three patient developed a flexor allucis longus retraction. At a mean follow-up of 96 months (11–224), 28 patients were continuously disease free, 2 alive with disease, 6 died for the disease and 1 died for other causes. According with MSTS functional evaluation score, 20 patients were rated excellent, 11 good, 3 fair and 3 poor.
**Conclusions** Biological reconstruction with VFG resulted a reliable long lasting reconstructive option after bone tumor resection of the femur.

### Porous tantalum implants in primary and revision tumor surgery of the pelvis and lower extremity

N. Fabbri*, P. Ruggieri, A. Angelini, M. Mercuri

Istituto Ortopedico Rizzoli (Bologna, IT)

**Introduction** Porous tantalum was very successfully used during the last decade in joint revision surgery associated with severe bone defects. Purpose of this study is to analyse clinical results obtained using modular porous tantalum implants in primary reconstruction after tumor resection or in revision surgery of a pre-existing failed tumor reconstruction of the pelvis and lower extremity.
**Materials and methods** We present a retrospective study of 17 patients (7 male, 10 female) who underwent revision surgery for a failed tumor implant (15 cases) of hip-pelvis (9 cases), knee (5 cases) and ankle (1 case), and of 3 primary reconstruction of hip-pelvis after surgical management of bone malignancy (2 cases) or reconstruction of a massive non-oncologic pelvic defect (1 case); average age at the time of surgery was 35 years (22–55 years). Cause of failure in revision cases was aseptic loosening (9 cases) or deep infection (6 cases). Revision of infected cases was managed in 2 or more stages. All patients presented severe segmental bone defect as result of primary tumor management and/or recent cause of failure. Bone defect was managed in all cases with modular porous tantalum implants uncemented at the host bone interface and cemented in contact areas with "augments", always used, in association with morcellised bone grafts to fill residual cavitary defects a riempire i difetti cavitari residui and with a megaprosthesis in 9 cases (6 proximal femur, 2 distal femur, 1 proximal tibia). Minimum follow-up in all cases was 2 years, average follow-up was 4.8 years (2–8 years).
**Results** In 1 case there was recurrent infection requiring further surgical management. Porous tantalum implant made revision surgery easier and showed excellent features also when used for primary reconstruction, especially in the pelvis. In all cases the porous tantalum implant is well-fixed and functioning at last follow-up.
**Discussion** Porous tantalum has provided solid and reliable fixation in difficult situations from biomechanic standpoint, where it seems to be superior to alternative reconstructive techniques.
**Conclusions** Porous tantalum provided very satisfactory clinical performance at a medium follow-up approaching 5 years and is extremely promising in musculoskeletal oncology as articular and segmental reconstructive technique. Longer follow-up is necessary to identify later potential failures.

### Unicondylar allografts for reconstruction of the knee after condylar bone loss following tumor surgery or trauma

G. Scoccianti*, D.A. Campanacci, G. Beltrami, D. Matera, R. Capanna

SOD Ortopedia Oncologica, AOU Careggi (Florence, IT)

**Introduction** Wide bone loss affecting a femoral or tibial condyle with involvement of the joint surface is a challenging condition for the surgeon. To avoid a total joint resection and a megaprosthesis reconstruction in patients who are often young, the use of unicondylar allografts was proposed, but in Literature few series were reported. Aim of our study was to evaluate the results of knee reconstruction using unicondylar allografts in our experience with a follow-up from 4.5 to 8 years.
**Materials and methods** Six patients underwent a unicondylar resection on the femoral or tibial side of the knee joint and a reconstruction with a fresh-frozen condylar allograft. Three patients were affected by a primary tumor; three patients were affected by bone loss after knee trauma. The femur was involved in 4 cases and the tibia in 2 cases. Follow-up ranged from 57 to 99 months (mean 77.5). At follow-up all patients underwent a clinical and radiographic examination. Functional evaluation was performed according to MSTS-ISOLS Score and to Knee injury and Osteoarthritis Outcome Score (KOOS), a knee-specific evaluation system.
**Results** In all cases the osteotomy line healed uneventfully. None of the patients required further surgical procedures, with the exception of one patient who suffered a distal femur fracture 1 month after surgery and underwent a procedure of reduction and internal fixation. Patients affected by tumor were continuously disease free at the time of latest follow-up. MSTS-ISOLS score ranged from 18 to 27. Range of motion restoration was satisfying with complete extension and flexion $\geq 90°$ in all patients. Two patients used a brace to enhance stability. Radiographic evaluation showed an early occurrence of signs of degenerative osteoarthritis, but this did not show a clinical significance by now.
**Discussion** In our series clinical results at an average follow-up of more than 6 years are satisfying and show that a functional knee can be obtained avoiding wider procedures requiring a megaprosthesis. Longer follow-up and a multicenter study to enroll wider numbers of patients are needed to verify long-term results.
**Conclusions** When dealing with wide bone losses affecting a tibial or femoral condyle, the use of an unicondylar allograft at first surgery is an important option to postpone the implant of a prosthesis in young patients, and it can provide a better bone stock at the moment of a second procedure with joint replacement.

### Surgical treatment of metastatic renal carcinoma of the cervical spine

L. Babbi*[1], A. Gasbarrini[1], S. Bandiera[1], G. Barbanti Brodano[1], S. Terzi[1], S. Colangeli[1], L. Boriani[1], R. Ghermandi[1], M. Cappuccio[2], S. Boriani[1]

[1]Istituto Ortopedico Rizzoli (Bologna, IT);
[2]Ospedale Maggiore (Bologna, IT)

**Introduction** The skeleton is the third cancer metastasis most common site after lung and liver. Vertebral metastases for anatomical reasons are probably the most common bone site involved. The operability, prognosis, the ability to take control of neoplastic disease by adjuvant therapy (chemotherapy, radiotherapy, immunotherapy) were determined according to the flow chart Gasbarrini-Boriani and we used the best treatment option to follow. The approach to these patients is multidisciplinary. Surgical treatment of metastases of the cervical spine presents specific problems for anatomical reasons.
**Materials and methods** We analyzed 433 patients surgically treated for spinal metastases from 1996 to 2011. 108 cases of metastatic renal cell carcinoma were considered. 8 patients (7%) showed localization to the cervical spine. The average age of these patients was 57.4 years (range 39–78 months). In all patients, embolization was performed pre-surgery to reduce intraoperative blood loss. The surgical options were schematically divided into: decompression and stabilization of 3 cases (palliative treatment), 5 cases debulking (intralesional excision). The neurological status of patients was assessed before and after surgery according to the classification of Frankel modified.
**Results** The average follow-up period was 17 months (range 3–37): 1 NED (not evidence of disease), an AWD (alive with disease), 5 died, 1 patient lost to follow-up. In all cases post-operatively there was an improvement in Frankel neurological score. There were two complications: (1) a post-operative dysphagia spontaneously regressed and (2) after 2-year follow-up, an ulceration of the skin scar with the synthesis tool projection, in the latter case a reoperation was performed.
**Discussion** For anatomic reasons, cervical spine is the spine site where it is more difficult to obtain local control of metastatic lesion, especially in tumors that have a poor response to adjuvant therapies. Currently there are any specific work in the literature about the treatment of metastatic renal cell carcinoma of the cervical spine to compare our data.
**Conclusions** The aim of this study is to obtain a homogeneous series for histology and anatomic site of the metastatic lesion and to obtain an objective evaluation of medium to long term results.

### *AT05—SHOULDER AND ELBOW 1*

### Arm Squeeze Test: a new clinical test for differential diagnosis between cervical spine and shoulder disorders

S. Gumina, S. Carbone*, G. Ippolito, P. Albino, F. Postacchini

Università di Roma Sapienza (Rome, IT)

**Introduction** Objective of this study is to evaluate the diagnostic values of the Arm Squeeze Test. The test consists in squeezing the middle third of the upper arm (biceps area). Our hypothesis is that squeezing is responsible for pain only in patients with cervico-brachialgy.
**Materials and methods** 1567 patients were included in this study. Diagnosis was clinically and strumentally formulated before performing the test and patients were subdivided as it follows: 903 with rotator cuff tear, 155 with primary shoulder adhesive capsulitis, 101 with arthropathy of acromioclavicular (AC) joint, 55 with calcifying tendonitis, and 48 affected by gleno-humeral arthritis. The study sample included 305 patients with cervico-brachialgy involving one or both shoulders. 350 healthy were recruited as controls. The test was positive when the score on a VAS Scale was 3 points or higher on pressure on the middle third of the upper arm compared with to the AC joint and anterolateral subacromial area.
**Results** The test was positive in 295/305 (96.7%) of patients with cervical spine disorders, compared to 35/903 (3.87%), 3/155 (1.93%), 0/101 (0%), 1/55 (1.81%), 4/48 (8.33%), and of those with rotator cuff tear, adhesive capsulitis, AC arthropaty, calcifying tendonitis and glenohumeral, respectively. A positive result was obtained in 14/350 asymptomatic subjects (4%). If cervicobrachialgy was compared to the other five conditions, the test had a sensitivity from 0.97 to 0.99 and a specificity from 0.93 to 0.99. Respect to controls, the sensitivity was 0.98 and the specificity was 0.97.
**Discussion** In case of shoulder pain, differential diagnosis between cervical and shoulder pathology can be difficult.
**Conclusions** The Arm Squeeze Test may be useful to distinguish a cervico-brachialgy from shoulder disease in case of doubtful diagnosis.

### Mid-term clinical outcomes of shoulder resurfacing arthroplasty in correlation to aetiology

H. Bloch*[1]

Orthopaedic University Hospital (Rostock, DE)

**Introduction** The aim of this retrospective study was to evaluate clinical outcomes of a modular resurfacing shoulder arthroplasty in correlation with aetiology.
**Materials and method** 39 patients (average age $61.77 \pm 11.59$ years) with a modular resurfacing shoulder arthroplasty were evaluated with an average follow-up of $42.9 \pm 7.8$ months. Primary diagnoses were cuff tear arthropathy (46.1%), primary osteoarthritis (38.5%), secondary osteoarthritis (10.3%) and rheumatoid arthritis (5.1%). A concentric glenoid erosion was observed in 92.3%, an eccentric one in 7.7%. 61.5% had type-A1 morphology, 28.2% A2 and 10.3% B1. The rotator cuff was intact in 28.2%, attenuated in 18%, with minor tear in 43.5% and massive tear in 10.3%. In 6 cases of massive cuff rupture, a resurfacing CTA head was used. Constant Score (CS) and x-rays were evaluated in correlation to glenoid erosion and cuff status.

**Results** The average CS increased from 26.9 ± 9.9 preoperatively to 66.6 ± 19.6 at the last follow-up ($p < 0.001$). In case of cuff tear, the percentage CS increase was more relevant with the CTA head than with a cuff repair that had variable results. Pain relief was improved in case of intact or attenuated cuff ($p < 0.001$), but not in case of cuff tear or glenoid erosion. ROM improved significantly, with an average FF from 78.68° ± 25.14° preoperatively to 153° ± 25.59° postoperatively. Active ER improved from 18.68° ± 15.84° to 42.12° ± 15.26° and was influenced by glenoid erosion. Active IR improved from buttock level (60.61%) to waist (48.5%). One case of non-progressive radiolucent line and 2 cases of osteolysis underneath the cup with no instability were observed. 6 revisions occurred due to worsening of the cuff status.

**Conclusions** Mid-term satisfactory results can be obtained in case of a good rotator cuff status. The glenoid resurfacing is recommended in case of glenoid erosion with good cuff and the CTA head with cuff tear arthropathy.

### Analysis of agreement between computed tomography measurements of glenoid bone defect with and without comparison with the contralateral shoulder

M.F. Saccomanno*, L. Deriu, C. De Ieso, G. Logroscino, G. Milano, C. Fabbriciani

Istituto di Clinica Ortopedica, Università Cattolica del Sacro Cuore (Rome, IT)

**Introduction** Glenoid bone defect is frequently associated with anterior shoulder instability and is considered as one of the major cause of recurrence of instability. Computed tomography (CT) is the method of choice to assess and measure glenoid bone defect. However it is still not clear which method (2D or 3D) is more reliable. The purpose of the present study was to evaluate agreement between assessment of glenoid bone defect with and without comparative study with the contralateral shoulder, on two-dimensional (2D) and three-dimensional (3D) computed tomography (CT) scans.

**Materials and methods** One hundred patients affected by unilateral anterior shoulder instability underwent a CT of both shoulders. Images were processed with both 2D and 3D methods. Area of the missing glenoid was calculated with the circle method, and expressed as percentage of the entire circle fitting the inferior glenoid, with and without comparison with the contralateral healthy shoulder. Agreement between measurements obtained with and without comparative study was assessed according to the Bland–Altman method. Agreement between the two methods in detecting the presence of a bone defect and discriminating the type of the defect (fracture or erosion) were also evaluated by using percent agreement and K statistics.

**Results** Analysis of agreement between measurements (with and without comparative study) of the size of the bone defect showed a mean difference equal to −0.42% + 2.05%, and 0.02% + 1.37% for 2D and 3D CT scans, respectively. Percent agreement between the two measurements to detect the presence of bone defect was 93% ($k$ coefficient = 0.85; $p < 0.0001$), and 94% ($k$ coefficient = 0.87; $p < 0.0001$) for 2D and 3D CT scans, respectively. Percent agreement between the two measurements to discriminate the type of bone defect was 93% ($k$ coefficient = 0.88; $p < 0.0001$), and 94% ($k$ coefficient = 0.90; $p < 0.0001$) for 2D and 3D CT scans, respectively.

**Discussion** CT assessments of glenoid bone defect with and without comparative study with the contralateral shoulder showed a very good agreement in identifying the size, presence, and type of the defect in patients with anterior shoulder instability, on both 2D and 3D CT scans.

**Conclusions** Measurements of glenoid bone defect on 2D scans and 3D scans are interchangeable.

### Comparison of clinical outcomes of reverse shoulder arthroplasty with glenospheres of different designs, diameters and materials

A. Castagna*[1], H.R. Bloch[2], A. Bishof[3], P. Budassi[4], J.D. Agneskirchner[5], M. Borroni[1], C. Domenghini[2], M. Frattini[4], S. Zoni[2]

[1]IRCCS Istituto Clinico Humanitas Rozzano (Milan, IT);
[2]Ars Medica Clinic (Gravesano, CH);
[3]Orthopaedie am Rosenberg (Heiden, CH);
[4]Istituti Ortopedici (Cremona, IT);
[5]Sportsclinic (Hannover, DE)

**Introduction** The aim of this retrospective study was to compare ROM, pain, scapular notching and stability of reverse shoulder prosthesis performed with glenospheres of different diameters, designs and materials.

**Materials and methods** 133 patients (average age 69.2 years, 31% male, 69% female) with a reverse shoulder prosthesis (SMR Reverse, Limacorporate, Italy) were divided into 3 groups: 60 (45%) patients with a 36 mm standard CoCrMo glenosphere (Group A), 21 (16%) with a 36 mm eccentric CoCrMo glenosphere (Group B), and 52 (39%) with a 44 mm cross-linked UHMWPE glenosphere (Group C). The average follow up was 38.3 ± 17.4 months. Primary diagnosis were: cuff tear arthropathy (A: 85%, B: 76%, C: 75%), secondary osteoarthritis (A: 3%, B: 14%, C: 15%) and cuff tears in endoprosthesis (A: 8%, B: 0%, C:8%). Clinical assessment included Constant score (CS), pain and ROM; radiographic analysis included scapular notching, instability and loosening.

**Results** The average CS significantly increased from preoperative to all postoperative time-points for all 3 groups ($p < 0.001$). Group C showed an average percentage CS increase (CS: +50%) more relevant than the others (A: CS: +31%, B CS: +43%; $p < 0.001$) at the last follow-up. At 12 and 24 months, Group B and C presented a more significant pain relief than Group A ($p < 0.05$) and Group C reached a higher and stable increase (active FF, active ER and IR; $p < 0.05$). The incidence of scapular notching was significantly lower in Group C than in B ($p = 0.001$) and A ($p = 0.009$), both at 12 and 24 months. The same trend was confirmed at the last follow-up. No progressive radiolucent lines have been observed. Group A had 5 (8.3%) early complications and Group C had 4 (7.6%).

**Conclusions** The study demonstrates promising mid-term results with SMR Reverse. 44 mm X-UHMWPE and 36 mm eccentric CoCrMo glenospheres allow a significant improvement of clinical outcomes, thanks to the inversion of the materials and the eccentric design, with even faster and more stable functional recovery with 44 mm glenospheres.

### *AT06—SHOULDER AND ELBOW 2*

### Effectiveness of the hyaluronic acid in the different stages of the evolutive cuff pathology: a perspective study

A. Busilacchi*, S. Cecconi, D. Enea

Clinica Ortopedica, Università Politecnica delle Marche (Ancona, IT)

**Introduction** Hyaluronic acid (HA) is a widely used molecule in shoulder pathology, administered through the intra-articular/sub-acromial way. Indications for these injections are: rotator cuff tear, Duplay disease, frozen shoulder, osteoarthritis, etc. Although several studies were published, literature still lacks in details about the pertinence in the indications. Goal of the present study was to point out the correct indication for HA injective therapy through a perspective study: firstly defining the safety and efficacy of HA in the different stages of cuff tears, then evaluating the effectiveness at a long term follow up (90 days) as secondary endpoint.
**Materials and methods** During the period Jan 2007-Oct 2008, using strict recruitment criteria, 100 patients suffering of cuff pathology were enrolled. The population of study were divided into 4 groups according to Neer classification with a fourth added group (cuff-tear-arthropathy). Each patient underwent a cycle of 3 US-guided injections of HA (Sinovial-IBSA 0.8–16 mg/2 ml), every 15 days. To perform the injection, antero-lateral way was used. Follow up was planned every 15 days (t0, t15, t30, t45 e t90), using VAS, Oxford-Shoulder-Score (OSS) and Constant-Murley.
**Results** *Primary endpoint*. In stage I and II, at day 30 of FU, a significant reduction of VAS and increase of Constant-Murley and OSS was recorded. In 4 stage benefits were recorded for the first 45 days, while OSS and Constant did not show any improvement.
*Secondary endpoint* was satisfied for stage I, II and IV. All data are statistically significant (Split-Plot analysis/Scheffé Method). Adverse or side effects occurred in a lower percentage than reported in literature.
**Discussion** HA in cuff pathology represents a valid and safe alternative to other conservative treatments, in particular to corticosteroids. HA prescription should be made in appropriate case: the best results were reached in patient with bursitis (grade 1) or partial tear (grade 2). Not a big benefit is reported in patients with complete cuff tear (grade 3), while patients with osteoarthritis (grade 4) feel few and short-lasting benefits.
**Conclusions** These data allow to consider HA therapy particularly useful for bursitis or partial cuff tear, while in presence of cuff tear arthropathy it must be considered just to delay surgery as well as a temporary solution looking forward to arthroplasty. In complete cuff tear, HA is not effective and arthroscopic surgery still remains the best option.

### The safe zone for avoiding suprascapular nerve injury: an anatomical study on 500 dry scapulae

S. Gumina, P. Albino, M. Giaracuni*, S. Carbone, F. Postacchini

Università di Roma Sapienza (Rome, IT)

**Introduction** Suprascapular nerve injury may be a complication during shoulder arthroscopy. Our aim was to verify the reliability of the existing data, assess the differences between scapulae in the two genders and in the same subject, obtain a safe zone useful to avoid iatrogenic nerve lesions, and analyze the existing correlations between the scapular dimensions and the safe zone.
**Materials and methods** We examined five hundred dried scapulae, measuring six distances for each one, referring to the scapular body, glenoid and the course of the suprascapular nerve, also catalogued according to gender and side. Differences due to gender were assessed comparing mean ± SD of each distance in males and females; paired *t* test was used to compare distances deriving from each couple. Successively we calculated our safe zone and Pearson's correlation.
**Results** We found non-significant differences between the right and left distances deriving from each couple; differences due to gender were stated. We defined three kinds of safe zones referring to: 500 scapulae; males (139 scapulae) and females (147 scapulae). The correlation indexes calculated between the axis of the scapular body and glenoid and the posterosuperior distance (referring to the suprascapular nerve) were 0.624, 0.694, 0.675, 0.638; while those with the posterior distance were 0.230, 0.294, 0.232, 0.284.
**Discussion** Knowledge of the safe zone, for avoiding suprascapular nerve injury, is important; gender and specific scapular dimensions should be evaluated since they influence the dimensions of the safe zone.
**Conclusions** The linear predictors should be used to obtain specific values of the posterosuperior limit in each patient.

### Chronic A/C joint dislocation: surgical treatment with biological ligament versus artificial ligament

F. Fauci*, P. Paladini, F. Campi, G. Porcellini

U.O. Chirurgia Ortopedica della Spalla (Cattolica, IT)

**Introduction** A/C joint dislocation is a frequent trauma sequelae and there are more than sixty different surgical techniques to treat it varying from biologic to artificial ligament reconstruction.
**Materials and methods** In this prospective study we evaluated, at 4-year follow-up, clinical and radiographic outcomes of patients treated for reconstruction of Coraco-clavicular ligament with LARS-LAC artificial ligament or semitendinosus tendon from tissue bank. We enrolled forty consecutive patients, half treated with biologic ligament and half with artificial ligament. All the patients were evaluated at T0 (surgery-time), T1 (2-month follow-up), T2 (6 months follow-up), T3 (12-month follow-up), T4 (24-month follow-up). Used outcome scores was UCLA. Radiographic scans were: anterior-posterior of both shoulders, without weights, and axillary views. X-ray imaging was used to evaluate the following features: maintenance of A/C joint reduction, A/C joint ossifications, A/C joint arthritis, clavicle osteolysis.
**Results** *Group A*: biologic tendon as coraco-clavicular ligament.
T1 and T2 examinations: 95% of UCLA outcomes scored as good, 1 case of posterior subluxation of the clavicle, 12 cases of painful swelling of the scar, no cases of clavicle osteolysis on screw sites, no cases of heterotopic ossifications, no cases of A/C joint arthritis.
T3 and T4 examinations: 85% of UCLA outcomes scored as good, 3 cases of posterior subluxation of the clavicle.
*Group B*: artificial ligament as coraco-clavicular ligament.
T1 and T2 examinations: 16 cases of good maintenance of A/C joint reduction.
T3 and T4 examinations: 3 cases of recurrence of A/C joint dislocation.
**Discussion** Surgical reconstruction of coraco-clavicular ligaments with biologic ligament permits a good stabilization on longitudinal and transversal plane. This features is not permitted with artificial ligament. The A/C joint stabilization on longitudinal and transversal plane is fundamental to achieve a stable reduction of the clavicle.
**Conclusions** Surgical treatment of chronic A/C joint dislocation with biological ligament is a valid alternative to use of artificial ligament.

### Comparative study of compression versus locking plates in distal humerus fractures

G. Giannicola*, A. Greco, F.M. Sacchetti, G. Bullitta

Università di Roma Sapienza (Rome, IT)

**Introduction** Distal humerus fractures are severely disabling injuries and represent 0.5–2% of all fractures and 30% of elbow fractures. Treatment of these fractures is very challenging, especially in elderly patients. These injuries are classified according to Jupiter and the AO\ASIF classification system. The objective of this study was to compare results of distal humerus fractures treated with compression or locking plates of the Congruent Elbow Plate System (CEPS).
**Materials and methods** Between 2005 and 2009, 22 patients with articular fracture of the distal humerus were treated surgically with CEPS, all by the same surgeon. In the first 12 patients (group I), a compression system was used, while in the last 10 patients (group II) a locking plate system was used. In the first group, the 90° or 180° plate configuration was chosen on the basis of the fracture pattern and to obtain greater stability, while a 180° configuration was used in all patients of the second group. Post-operatively, all patients started independent active rehabilitation beginning from the second post-operative day and wore an unlocked hinged splint. All patients had indometacin prophylaxis against HO. The results were valued radiographically and clinically with the MEPS. The minimum follow-up was 1 year.
**Results** The average post-op MEPS was 84 in group I and 91 in group II. Three complications were observed in group 1: one non-union, one fixation failure and one reduction failure. The last two complications were found in two elderly patients. Extrinsic elbow stiffness was observed in three patients in group II; these complications were related to post-operative ulnar nerve neuropathy in one case and poor compliance in the other two cases.
**Discussion** Our study highlighted satisfactory results in the majority of cases of both groups, with an average MEPS of 86. The use of compression or locking plates (90° or 180° configuration) does not seem to have significantly influenced the clinical result. This is in accord with other studies and confirms that this type of osteosynthesis is just one of the factors which influence treatment results. Early treatment, correct surgical approach, perfect anatomical reduction, HO prophylaxis, early mobilization and patient compliance represent other very important prognostic factors. Nevertheless the different complications observed in the two groups suggest that the use of locking plates is indicated especially in comminuted fractures and in patients with poor bone stock.

### Evolution of radial head's fractures treatment

G. Caruso*, A. Aquino, D. Lazzara, F. Del Prete, L. Preziuso, A. Petrini

SODC Ortopedia e Traumatologia, ASF Firenze, Ospedale SGD (Florence, IT)

**Introduction** Radial head fractures account for approximately 20% of elbow injuries and are often treated conservatively. The traditional criteria for surgical indication considered: joint involvement, the patient's age and comminution. In recent years, treatment of injuries of the radial head (and any associated ligament injuries) has changed. Alternative techniques (radial head new prosthesis, fixation techniques and ligament reconstruction) are in fact spreading. Today, in the choice of therapy, in addition to traditional criteria, we consider the overall stability of the elbow. The non-operative treatment still maintains its validity for compound fractures with good elbow stability. For the classification of the injuries we use Mason's criteria (indication for osteosynthesis is given in some of type I and in all type II fractures, indication of prosthetic replacement for type III and in some of type IV fractures).
**Materials and methods** From January 2006 to December 2010 we treated 130 radial head's fractures. 27 of these (20.1%) were treated surgically (16 females, 11 males, age range 13–90 years). In one case the fracture was exposed, in 7 cases there was an acute dislocation of the elbow. In one case dislocation was chronic. In 7 cases an ulnar's fracture was associated. In the 27 operated cases, we performed 15 osteosynthesis, 6 prosthetic replacement, 3 total resection and 3 partial resection of the radial head. In 16 cases we associated a ligament reconstruction. In 2 cases of severe instability was also applied a temporary external fixator.
**Results** Patients were evaluated using the MEPI and DASH forms. We obtained excellent results in 15 cases, fair in 7, sufficient in 3, poor in 2 (an osteosynthesis complicated by algodystrophy and stiffness, who was re-operated after 6 months to remove the plate and to perform surgical mobilization and one case of partial resection who required a new surgical procedure of prosthetic replacement).
**Discussion** The treatment of fractures of the radial head has changed in recent years. Whenever possible, osteosynthesis is performed. When a safe osteosynthesis is not possible, prosthetic replacement is a valid alternative, even if, in selected cases, radial head's resection is still a possibility of treatment.
**Conclusions** With the news on the biomechanics of the elbow, we require greater attention to the reconstruction of anatomical structures. Therefore, today more than ever, it is essential to make a correct diagnosis, considering the radial head not solely but as a part of the whole elbow joint.

### *AT07—FOOT*

### Mini-fixators in the treatment of brachymetatarsia

S. Reverberi*

Casa di Cura Villa Verde (Reggio Emilia, IT)

**Introduction** Brachymetatarsia is a congenital deformity presenting as an unilateral or bilateral brief metatarsal bone; it generally concerns the fourth toe. Many surgical techniques can be used to treat this deformity. In this paper we are interested in Ilizarov technique, which uses an external mini-fixator. Ilizarov was the first surgeon to lengthen finger's phalanges without bone transplant; his method used an heavy hardware, barely tolerated by patients. With new external fixators and with a modified surgical technique operation is very simple, fast and better tolerated by the patients.
**Materials and methods** With an Ilizarov modified technique we treated for brachymetatarsia 11 metatarsal bones. IV ray was always interested. 10 patients were females, 1 patient was male. Average age was 25 years (range 16–57 years). Metatarsal bones were lengthened 1.9 cm on average. We used 5 external fixators:

mini-Ilizarov; mini-Hoffmann 1 and 2; mini-elongator of Stryker SPA and mini-fixator Mikai. Lengthening was completed at a speed of 0.5–1 mm/day.

**Results** Regenerated bone consolidation was obtained in all but one case (treated with bone graft); the use of external fixation time differed widely depending on the length to be achieved, usually 3–6 months. In 2 patients, the mini-stretch was increased to 10 months, one of these patients required the application of a bone graft. The most frequent complication, apart from the secretion by the small exit hole of the wires (which however disappeared after removal of the implant) was angular deviation during the stretch and a slight limitation of movement of the Fourth MTF. All patients were satisfied with the results and improved aesthetic appearance and functionality.

**Discussion** The final result strongly depends on surgical technique, severity of the deformity, surgeon's experience and type of mini-fixator used. Each mini-fixator has unique characteristics, with advantages and disadvantages.

**Conclusions** The use of the new mini-lengtheners made metatarsal lengthening surgery much easier and safe in experienced hands.

### Reproducibility and results of SERI technique for the treatment of allux valgus: our early experience

A. Giuliante*, S. Conte, M. Iachelli, A. Zeri

Ospedale G.B. Grassi (Rome, IT)

**Introduction** The evolution of new materials, instrumentation and peripherical anaesthesiologic procedure carried to minimally invasive surgical approaches, shorter hospitalization time and an improvement of the patient's compliance.

**Materials and methods** Between January 2009 and December 2010 we performed 65 surgical procedures for hallux valgus in 57 patients. Mean age was 64 years. All cases were treated with SERI technique alone or associated with other metatarsal procedures such as Akin osteotomy, Jacoby osteotomy and other technique in HV revision. In 98% of cases the treatment was performed on day surgery. Radiologic pre-operative assessment was HVA 34° ± 6° and IMA 14° ± 4°. Post-operative care included weekly assessment, walking with talus foot, antithrombotic prophylaxis for 30 days while antibiotic prophylaxis was performed with cefazolina e.v. for 24 h. Kirschner wire removal was done after 32 days along with an X-ray control. After K removal we used an overcorrection bandage for 10 days followed by a finger rehabilitation. Clinical results were evaluated using AOFAS score.

**Results** Mean HV correction was 18° ± 7. In two cases, following a superficial infection, the Kirschner wire removal was done previously on 18° and 22° post-operative day.

**Conclusions** The clinical results of this technique were very surprising. The day surgery management, the lower post-operative pain, the good clinical results and the better cosmetic outcome for the shorter approach were well accepted by the patients and lead us to pursue HV surgery management.

### Influence of immunology on transplanted cartilage

F. Vannini*, R. Buda, M. Cavallo, A. Ruffilli, S. Neri, S. Natali, S. Giannini

Istituto Ortopedico Rizzoli (Bologna, IT)

**Introduction** Fresh bipolar osteochondral allograft (FBOA) is a procedure whose validity is still controversial for the treatment of the post-traumatic arthritis of the ankle. The immunological reaction to the cartilage graft may play a key role in the failure. Aim of this study is to compare two groups of patients treated with FBOA with or without post-operative immunosuppressive therapy.

**Materials and methods** Two groups of 20 patients received FBOA. Only one group (group B) received immunosuppressive therapy. A clinical (AOFAS score) and radiographic evaluation (Rx, TC, RMN) were performed pre-operatively and at the last follow-up. A biopsy of the transplanted area was obtained at 1-year follow-up for histological, immunohistochemical (ICRS II score) and genetic examination.

**Results** In the group A the AOFAS score improved from 28.2 ± 10.9 (pre-operatively), to 69.9 ± 18.2 at 24-month follow-up ($p < 0.005$), while in the group B improved from 26.2 ± 6.8 (pre-operatively) to 71.4 ± 7.3 at the same follow-up ($p < 0.005$). The comparison between the clinical results of the two groups was not statistically significant. The group B showed a better morphology of the graft (average ICRS II score 68%) in comparison to the group A (40%) ($p < 0.05$). Genetic analysis showed the presence of both the donor and recipient DNA. We found a clear correlation in the two groups between Kendall $t$ and the ICRS score. All samples scored 100 belonged to the group B, while all the samples scored 0 belonged to the group A ($t = 0.506$, $p = 0.008$).

**Discussion** Although clinical results were found to the be similar in both groups, a better histological appearance of the graft is evident in the group B, in which osteochondral allograft was significantly better preserved.

**Conclusions** Genetic analysis showed the presence of cells with the phenotype of the recipient in the grafted areas, whose meaning is still to be further investigated.

### Foot rescue with the Ilizarov method in the late complications of compartment syndrome

A. Kirienko*[1], A. Peccati[2], N. Portinaro[2]

[1]Istituto Clinico Humanitas (Rozzano-Milan, IT);
[2]Università degli Studi di Milano (Milan, IT)

**Introduction** Compartment syndrome is one of the most known among the complications of lower limb lesions, often related to fractures and massive or crush injuries. Clinically, the foot has equinus and cavus deformities, with consequent varus deformity of the calcaneus and a claw toes deformity. The treatment aim is to get a plantigrade, functional and painless foot.

**Materials and methods** We treated 16 patients with the distraction method for foot deformities and late complications derived from neuro-vascular damage. The patients' mean age at the time of the lesion was 24 years, and the mean age at the time of surgery was 26 years. Ten patients had a pilon fracture, 2 had a knee luxation, 1 had a crush injury of the pilon, 1 had a sciatic nerve stretch due to traumatic hip luxation, 1 had a distal femur fracture, complicated by a traumatic lesion of the common femoral artery and the sciatic nerve. 6 patients had a skin ulcer at the base or the head of the 5th metatarsal bone. 8 patients had been proposed a limb amputation because of the pain or the presence of the ulcer or infection.

**Results** We performed a two-steps treatment, firstly applying the frame for the closed correction of the equinus deformity of the foot (after the Achilles tendon lengthening and the correction of the adduction and supination of the forefoot and the correction of the anterior or mixed cavus deformity of the foot). The mean distraction time was 52 days, followed by a mean 51 days waiting period. After that we performed foot stabilization by pan-talar arthrodesis (tibial-talar, sub-talar, talo-navicolar and calcaneo-cubiod joints) in 9 patients and triple arthrodesis in 5 patients; in 2 patients no stabilization was performed. The mean in-frame time to achieve stabilization was 63 days. All the deformities have been corrected with a good functional outcome; a partial recurrence of the deformity has been observed in 3 cases. Five out of the 6 patients affected by skin ulcer fully recovered.

**Conclusions** The closed mini-invasive technique of distraction with the Ilizarov circular external fixation, along with secondary foot and ankle stabilization, can dismiss leg amputation in patients affected by this complex clinical picture.

### Validation of the Ottawa ankle rules in a second level trauma center

M. Marinelli*, S. Cecconi, A. Busilacchi, L. De Palma

Cattedra di Ortopedia e Traumatologia, Università Politecnica delle Marche, Azienda Ospedaliero-Universitaria, Ospedali Riuniti di Ancona (Ancona, IT)

**Introduction** Trauma of the foot and ankle is commonly seen in patients in the Emergency Unit. Almost all these patients undergo X-rays even though the result is expected to be normal. In fact, only a small percentage of patients—approximately 15%—have clinically significant fractures. The Ottawa Ankle Rules (OARs) were designed to reduce the number of unnecessary radiographs in these patients. The objective of this study was to validate the OARs in an Italian Trauma Center.

**Materials and methods** This prospective survey was done among 248 patients with acute ankle injury from July 2006 to October 2006. Main outcome measures of this survey were: sensitivity, specificity, positive predictive value, negative predictive value, and likelihood ratios (positive and negative) of the OARs.

**Results** Sensitivity of the OARs for detecting 42 ankle fractures was 100% for each single zone and for both zones. Specificity of the OARs for detecting fractures was 46.5% for both zones, 43.5% for the malleolar zone, and 41% for the midfoot zone. Implementation of the OARs had the potential for reducing radiographs by 29%.

**Discussion** OARs are very highly sensitive tools for detecting ankle and mid-foot fractures. Implementation of these rules would lead to reduction in the number of radiographs, costs, radiation exposure and waiting times in Emergency Departments.

### *AT08—BASIC RESEARCH*

### G-CSF and osseointegration of bone substitutes: a possible opportunity?

A. Marmotti*, F. Castoldi, R. Rossi, S. Marenco, M. Bruzzone, F. Dettoni, D.E. Bonasia, P. Rossi, C. Tarella

Università di Torino (Turin, IT)

**Introduction** Granulocyte-colony-stimulating-factor has been used to improve repair processes in different clinical settings for its role in bone-marrow (CD34+ and CD34−) stem-cell mobilization, besides for its trophic effects mediated by G-CSF-Receptor. Evidences in literature suggest G-CSF may also play a role in skeletal-tissue repair processes. Osseointegration at bone-substitute interface might benefit from the mobilization of bone-marrow-derived-cells (BMC) by G-CSF. Aim of the study was to verify feasibility, safety and efficacy of preoperative-BMC-mobilization by G-CSF in patients undergoing high-tibial-valgus-osteotomy (HTVO) for genu varum.

**Materials and methods** Twenty-four patients were enrolled in a prospective phase II trial. The osteotomy gap was filled by hydroxyapatite-tricalciumphosphate-bone-substitute. Two consecutive cohorts of 12 patients were assigned to receive (GROUP-A) or not receive (GROUP-B) a daily dose of 10 μg/kg of G-CSF for 3 consecutive days, with an additional dose 4 h before surgery. BMC-mobilization was monitored by WBC-count and flow-cytometry-analysis of circulating CD34+ cells. All patients underwent: (1) clinical score (Lysholm and SF-36); (2) X-ray-evaluation preoperative and at 1, 2, 3, 6, 12 months after surgery to compare the percentage of integration at the interface between host bone and bonesubstitute; (3) CT-scan of the host bone-substitute interface at 2 months to estimate the osseointegration through a semiquantitative score and a measure of bone density.

**Results** All patients of both groups completed the study. The most common adverse events in Group-A were mild to moderate bone pain and muscle discomfort. There were no severe adverse events. Mean preoperative WBC and CD34+ values were 37.17 × 103/μl (20.84–51.11) and 36.42/μl (29.2–40.4) in Group-A and 6.54 × 103/μl (2.8–11.1) and 7.03/μl (4.76–11.06) in Group-B, respectively. Patients of Group-A displayed a slight increase in overall performance at 3 and 6 months compared to Group-B ($p < 0.05$). At semi-quantitative X-ray-evaluations, a higher rate of bone substitute osseointegration was observed in Group-A at 2, 3, 6 months post-surgery ($p < 0.05$) compared to Group-B. CT-scan: bone density at the host bone-substitute interface (Hounsfield unit) was lower in Group-A compared to Group-B, according with an advanced stage of bone remodelling ($p < 0.05$).

**Discussion** G-CSF-administration given to induce pre-operative-mobilization of bone-marrow-derived-cells is feasible and safe in patients undergoing orthopaedic surgery, it allows the peripheral blood circulation of high numbers of CD34+ cells and it may hasten bone-graft-substitute integration, as suggested by both clinical, radiographical and CT evaluations.

**Conclusions** The enhanced osseointegration might be the result of a direct activity of G-CSF on host bone or of a cellular effect mediated by of bone marrow-derived-progenitors mobilized by G-CSF or by the combination of all these factors. Further studies will be aimed to clarify the underlying mechanisms of G-CSF on bone activity.

### Histomorphometric and SEM analysis of the osteointegration process of a titanium implant versus a type I collagen titanium implant: in vivo study

S. Cecconi*, M. Torcianti, S. Manzotti

Clinica Ortopedica, Università Politecnica delle Marche (Ancona, IT)

**Introduction** Experimental research is trying to find what is the best surface to enhance the osteointegration of metal implant. First results were obtained by the introduction of nanostructured surfaces, in particular for titanium. Nowadays there is an increasing interest about the possibility of coating titanium implants with osteoconductive substances. The aim of the present study is the evaluation of the osteointegration process of a titanium implant versus a type I collagen titanium implant.

**Materials and methods** Both types of titanium implants were introduced into the femoral metaphysis of ten NZW adult rabbits. Six of them were sacrificed at 45 days and four at 90 days. During these periods a fluorochrome labelling for the histomorphometric analysis was used. On the samples obtained by abrasion with EXACT system the bone to implant contact (BIC) and the bone density (BD) of the titanium implants were valuated. On the same samples a SEM analysis was done.
**Results** The collagen I titanium implant showed an average BIC of 64.6 ± 19.3% versus 41.2 ± 22.4% of the untreated implant at 45 days. At 90 days it showed a BIC of 61.3 ± 2.5% versus 35.7 ± 20.1% of the untreated implant. In addition, the coated implants showed values of BD better than non-coated implant at 45 and 90 days, especially with regard to the bone to implant interface. These results were confirmed by SEM.
**Discussion** Results obtained by histomorphometric and SEM analysis confirmed, in the first instance, the biocompatibility of both types of implants. The statistically significant difference ($p < 0.001$) of BIC and BD values between the two types of implants, both at 45 and 90 days confirmed that the type I collagen coating allowed a greater osteointegration, due primarily to the osteoconductive ability of collagen itself.
**Conclusions** Our study and the literature suggest that the type I collagen coated titanium surface promotes osteointegration, increasing its stability, especially in the short term. These studies are focused on a possible use of type I collagen coating for dental implants, fixation devices or prosthetic titanium components.

### Role of estrogen receptor beta in the pathogenesis of lumbar disc herniation

S.A. Barnaba*, I. Sanzarello, L. Ruzzini, N. Papapietro, V. Denaro

Università Campus Bio-Medico di Roma (Rome, IT)

**Introduction** The aim of our study was to evaluate the expression of estrogen receptor beta on fragments of the intervertebral disc on a female population suffering from lumbar disc herniation. On the same sample, we searched the expression of matrix metalloproteinase 3 (MMP-3), focusing on a hypothetical correlation between estrogen receptor expression and disc degeneration degree (MMP-3).
**Materials and methods** Eleven samples from intrevertebral herniated disc were taken during lumbar spine surgery from female patients. Patients aged between 20 and 60 years (average 37). The location of disc disease related on 5 discs (L4-L5) and 6 discs (L5-S1). Two samples of healty intervertebral disc, from autopsy on female were included in the study and used as controls. Immunohistochemical reactions were performed with the Avidin–Biotin method.
**Results** The absolute number of cells in the samples of herniated disc had increased as compared to the healthy disc. Receptor beta expression was present in all samples tested with an average of positive cells of 45.9% on disease disc and of 14.3% in the control samples. MMP-3 was absent in the control samples while it was positive in all samples of herniated disc with an average of positive cells of 51.78%. Statistical analysis (Mann–Whitney test) showed a significance of both data ($p = 0.03$). We finally correlated with Spearman test the expression of estrogen receptor beta with that of MMP-3 ($p = 0.029$).
**Discussion** These data show a greater susceptibility of young-adult women of childbearing age to the formation of disc herniations. Degradative activity of the extra-cellular matrix, clearly shows disc degeneration and it is present and increased in patients with herniated disc. The estrogen receptor beta is widely increased in female population of childbearing age, compared to both female postmenopausal populations with lumbar disc herniations, and healthy controls.
**Conclusions** This study can be considered as an initial phase of the use of new immunoistochemical markers (beta-estrogen receptor and MMP-3) in the analysis of lumbar herniated disc pathogenesis, now in a female population, and in the future for male population too.

### Tenosynovitis and tendinosys: clinical data and anatomo-pathological considerations in the injuries of the long head of biceps

M.A. Rosa[1], G. Maccauro[2], S. Barreca[1], A. Foti[1], A. Laudati*[3]

[1]Sezione di Ortopedia e Traumatologia, Dipartimento di Specialità Chirurgiche, Università degli Studi di Messina (Messina, IT);
[2]Dipartimento di Scienze Ortopediche e Traumatologiche, Università Cattolica Roma (Rome, IT);
[3]UOS Chirurgia Artroscopica ASL RM E (Rome, IT)

**Introduction** The inflammatory and degenerative disease of the long head of biceps is usually characterized by an initial reaction with peritendinous synovial involvement. Clinically this pathology is characterized by the presence of an acute pain that usually decreases after a period of rest and it increases during activity. The main pain site is reported on the front of the shoulder, the irradiation is related to the anterior-medial region of the arm, sometimes spreading to the neck.
**Materials and methods** We report our experience using a histological study on 50 cases of staged withdrawal intra-articular portion tendon of the long head of humeral biceps obtained by open surgery or arthroscopy. The histological preparations were performed with hematoxylin-eosin and Masson trichrome. The average age of patients is 64.4 years. The most affected is the male gender.
**Results** The analysis of the research allows to detect the major histopathological features of evolution of the pathology of the humeral biceps. The initial stage of tenosynovitis is followed by an inflammatory reaction that progressively disrupts the fibers of the belly of the tendon: this makes a subversion with an alteration of the architecture of the tendon.
**Discussion** The pathogenesis is linked to a tenosynovitis with sclerotic thickening of the synovial sheath. Another important element is represented by the rupture of the rotator cuff as it determines the instability of the long head of biceps.
**Conclusions** The histopathologic changes take us to consider the need to adopt in shoulder surgery more stringent criteria for intervention to put an indication of tenotomy and tenodesis of the long head of humeral biceps. Nowadays shoulder surgeons send tendon samples for histological examination. The results of the histologic examination may be a protection for the patient as a possible demonstration of the degeneration of the tendon and it can be a valuable support in order to verify the adequacy of the decisions taken on the operating table.

### Comparison of bone metabolism and cellular activity in osteoporotic and young patients' fracture healing

U. Tarantino*, F.M. Liuni, J. Baldi, L. Saturnino, M. Feola, I. Cerocchi

Fondazione PTV, UOC Ortopedia e Traumatologia, Università degli Studi Tor Vergata (Rome, IT)

**Introduction** A successful repairing process depends on the complex balance between anabolic and catabolic stages of bone metabolism, determining healing speed and efficiency. Aim of this study is to compare the healing process in patients with fragility fracture compared to younger patients' fracture, through evaluation of cellular and inflammatory activity markers, and clinical-radiographic comparison.
**Materials and methods** The present study enrolled 56 patients of both genders: (1) *group A*—14 patients with femoral or tibial fractures treated with intramedullary nailing or external fixation (age range 26–48 years); (2) *group B*—14 patients with femoral fractures treated with intramedullary nailing (age range 55–89 years); (3) *group C*—14 healthy controls (age range 25–45 years); (4) *group D*—14 osteoporotic controls (age range 52–73 years). The inflammatory and metabolic activity was determined by specific serum markers: osteoprotegerin (OPG), osteocalcin (OCN), bone alkaline phosphatase (BALP), serum calcium, vitamin D3, parathyroid hormone (PTH), IL-6 and TNF-$\alpha$ The serum samples were collected within 24 h from fracture, within 24 h after surgery and at 10 weeks. Fractured patients were also evaluated by clinical questionnaire (Lower Extremity Measure) and radiographic scores (RUST score) after surgery and at 10 weeks.
**Results** In groups A and B, PTH increased pre-and postoperatively ($p < 0.001$). In groups B and D, OPG values were significantly higher than groups A and C ($p = 0.0008$). IL-6 showed an increase after surgery in groups A and B ($p < 0.001$), with higher values in group A compared to B ($p = 0.0063$). The radiographic comparison between fractured patients showed significant changes at 10 weeks postoperatively ($p = 0.0364$) with higher values in group A. The statistical analysis of results of the LEM questionnaire in fractured patients showed significant differences both after surgery and at 10 weeks ($p < 0.0001$), with higher scores in group A.
**Discussion** The initial increase in PTH levels in A and B groups could be caused by a response of the body to fracture event, expressing differentiation and proliferation of osteoblast and chondrocyte progenitor. The higher levels of OPG in osteoporotic patients showed a possible correlation of this marker with an higher bone turnover. Higher IL-6 in group A, could be a relevant factor in accelerating healing process of young fractured patients.
**Conclusions** The fracture healing process in osteoporotic patients is significantly compromised, with regard to inflammatory response, reduced osteoblast activity and clinical and radiographic evaluation.

### ORAL COMMUNICATIONS

### *C13—SPINE 1*

### Treatment of lumbar curves in adolescent females affected by idiopathic scoliosis with a progressive action short brace (PASB): assessment of results according to the SRS committee on bracing and nonoperative management standardization criteria

A.G. Aulisa*[1], L. Aulisa[2], M. Giordano[1], C. Perisano[2], A. Menghi[2], P. Savignoni[1], V. Guzzanti[1]

[1]Ospedale Pediatrico Bambino Gesù (Rome, IT);
[2]Università Cattolica, Policlinico A. Gemelli (Rome, IT)

**Introduction** The effectiveness of conservative treatment of scoliosis is controversial. Some studies suggest that brace is effective in stopping curve progression, whilst others did not report such an effect. The purpose of the present study was to effectiveness of PASB in the correction of lumbar curves, in agreement with the SRS Committee on Bracing and Nonoperative Management Standardisation Criteria.
**Materials and methods** Fourty adolescent females (mean age 12.95 ± 1.72 years) with lumbar curve and a pretreatment Risser score ranging from 0 to 2 have been enrolled. The minimum duration of follow-up was 24 months (mean: 41.75 ± 34.47 months). Anteroposterior radiographs were used to estimate the curve magnitude (CM) and the torsion of the apical vertebra (TA) at 5 time points: beginning of treatment (t1), 1 year after the beginning of treatment (t2), intermediate time between t1 and t4 (t3), end of weaning (t4), 2-year minimum follow-up from t4 (t5). Three situations were distinguished: curve correction, curve stabilisation and curve progression.
**Results** CM mean value was 26.43 ± 2.77 SD at t1 and 13.80 ± 7.94 SD at t5. TA was 10.83 ± 3.74 SD at t1 and 7.88 ± 4.24 at t5. The variation between measures of Cobb and Perdriolle degrees at t1, 2, 3, 4, 5 and between CM t5–t1 and TA t5–t1 were significantly different. Curve correction was accomplished in 82.5% of patients, whereas a curve stabilisation was obtained in 17.5% of patients.
**Conclusions** The PASB, due to its peculiar biomechanical action on vertebral modelling, is highly effective in correcting lumbar curves.

### Biomechanical aspects of idiopathic scoliosis evolution

A.G. Aulisa*[1], L. Aulisa[2], F. Falciglia[1], M. Galli[2], V. Guzzanti[1]

[1]U.O. di Ortopedia e Traumatologia, Ospedale Pediatrico Bambino Gesù (Rome, IT);
[2]Dipartimento di Ortopedia e Traumatologia, Policlinico A. Gemelli, Università Cattolica (Rome, IT)

**Introduction** In patients with idiopathic scoliosis, the interaction between biological and mechanical factors plays a central role in the evolution of deformities. According to the "vicious cycle model" of scoliosis evolution, the asymmetric load on the spine is the main factor driving the onset and development of deformities by altering the vertebral growth dynamics. Hence, once a critical asymmetric load has established, the progression of deformity is unavoidable, unless a compensatory force is applied to offset the biomechanical effects of growth. Here, we present a case series of adolescents with idiopathic scoliosis, in whom a normal vertebral morphology was achieved before the end of growth, who withdrew from the orthotic treatment during the growing age and maintained the correction over a 5-year follow-up.

**Materials and methods** Forty-six adolescents (40 girls and 6 boys) with idiopathic scoliosis treated with PASB or Lyon or Milwaukee brace, who achieved a complete curve and vertebral symmetry correction and withdrew from the treatment before the skeletal growth was complete. Participants presented with lumbar ($n = 17$), thoracolumbar ($n = 26$) or dorsal ($n = 3$) curve. Mean age at the beginning of treatment was 12.13 ± 2.16 years. All participants were prescribed with full-time bracing for an average of 53.35 ± 19.94 months. An early weaning was suggested, provided that a full-time bracing would be reinstituted if correction was lost. However, such a condition was not observed.

**Results** X-rays taken at the beginning of treatment showed a curve value of 23.93° ± 4.14° Cobb and Perdriolle value inferior to 15° Perdriolle. Radiologic examinations performed during the course of treatment evidenced a progressive reduction of vertebral rotation and lateral curvature, until the complete recovery of spinal geometry. At the end of treatment, all patients experienced a complete lateral curve correction. X-rays taken during the following 2 years showed a curve stabilization, with an average curve value of 4.80° ± 0.75° Cobb. Only 29 cases experienced a mild curve progression (7.62° ± 4.35°Cobb).

**Conclusions** The mechanical component is the major force involved in curve evolution. The restoration of a normal vertebral geometry via conservative treatment stops the scoliosis evolution and results in a permanent correction of the curve.

### Finite element modeling for biomechanical assessment of different lumbar fusions

R. Iundusi*[1], P.P. Valentini[2], J.F. Lin[3], F.M. Liuni[1], S. Auditori[2], F.L. Perrone[1], E. Pennestrì[2], U. Tarantino[1]

[1]Ortopedia e Traumatologia, Fondazione “Policlinico Tor Vergata” (Rome, IT);
[2]Dipartimento di Ingegneria Meccanica, Università degli Studi di Roma “Tor Vergata” (Rome, IT);
[3]Dipartimento di Chirurgia Vertebrale, Taipei Hospital (Taipei, Taiwan)

**Introduction** Lumbar interbody fusion is a commonly performed procedure for degenerative disc disorders and spinal instability. To obtain initial stability, six intervertebral movements need to be under control: flexion and extension, lateral bending, and axial rotation. Different fixation devices and techniques can provide sufficient initial stability though their biomechanical behavior is uncertain. In a preliminary clinical study it has been observed that a laterally inserted intravertebral plate with a cage can improve initial stability and enhance fusion. To compare the biomechanical properties of different lumbar fusion techniques on motion control we compared three models of lumbar interbody fusion: lateral cage alone, the same cage with an inter- and intravertebral plate, the cage with pedicle screws. Comparison of performances includes stress distributions and displacement control of these three configurations.

**Materials and methods** Three finite element models (FEM) of L3-L4, whose geometry was obtained from cadavers applying reverse engineering techniques, were implemented. The spatial models also include three fixation devices that were built by direct measurement on real components: stand-alone cage; cage and intravertebral plate; cage and pedicle screws. Six load conditions were analyzed.

**Results** The level of the mechanical stress inside the bones was similar for all the models. Concerning the displacements, the model with the pedicle screws showed a reduced level of mobility. The two models of stand-alone cage and intravertebral plate and cage behaved in a quite similar way. The presence of the plate had a very limited stiffening effect considering all the load conditions.

**Discussion** The solutions were compared taking into account the structural behaviour (stress and displacement) neglecting clinical consideration about insertion procedures. The results of the investigations showed that both the three solutions achieved the purpose of stabilizing the vertebral functional unit, i.e. constraining all the degrees of freedom between the vertebrae. On the other hand, finite element simulations underlined some important differences among the solutions.

**Conclusions** Biomechanical differences, when observed, did not produce abnormal intensifications or dangerous peaks. The presence of the intravertebral plate in the model 2 had a very limited stiffening effect considering all the load conditions providing a good movement control, especially in extension and axial rotation when compared to stand alone cage. The pedicle screw instrumentation showed a reduced level of mobility, especially when the vertebrae are loaded with a moment acting along transversal axis.

### Spinal infection multidisciplinary management project (SIMP): a new approach to spine infections

L. Boriani*[1], A. Gasbarrini[1], E. Zamparini[2], C. Nanni[2], G. Rorato[2], C. Salvadori[2], V. Allegri[2], S. Bandiera[1], G. Barbanti-Brodano[1], S. Terzi[1], S. Colangeli[1], A. Corghi[1], R. Ghermandi[1], L. Babbi[1], R. Ciminari[1], M.C. Malaguti[1], E. Rimondi[1], U. Albisinni[1], S. Fanti[2], P. Viale[2], S. Boriani[1]

[1]Istituto Ortopedico Rizzoli (Bologna, IT);
[2]Ospedale Universitario S. Orsola-Malpighi (Bologna, IT)

**Introduction** In 10 years, 130 patients affected by spine infections have been treated. The experience showed the absolute need of a multidisciplinary approach. A group of spine surgeons, interventional radiologists and anesthesiologists working in the Rizzoli Orthopaedic Institute and nuclear medicine and infective disease specialists, working in the Saint Orsola-Malpighi University Hospital in Bologna founded a work panel identified as SIMP.

**Materials and methods** Patients affected by spine infections were enrolled in a clinical case series prospective study following SIMP algorithm, whose Key point are: MRI with gadolinium in association to $F^{18}$-FDG PET/CT. CT-guided Trocar Biopsy. Antibiotic therapy characterized by first-line regimens as: the combination rifampicin + high-dose levofloxacin in the treatment of community-acquired infections or microbiologically documented infections by methicillin-sensitive *Staphylococcus* spp; the combination rifampicin + high doses teicoplanin in the treatment of post-surgical infections or microbiologically documented infections by MRSA. Surgical treatment was indicated only if the patient responds to eligibility criteria such as: wide abscess, progressive neurologic signs, instability/deformity, need for diagnosis, conservative treatment failure. Mininvasive stabilization technique was also taken into account in cases that need to be mobilized soon, with no abscess and with the chance to insert pedicular screws in healthy tissue. Medical therapy was stopped once the clinical pattern is solved and laboratory tests are in the standard range.

**Results** Preliminary results in 18 months are as follows: 32 patients treated, 12 studied with PET/CT before and after treatment. Nine patients underwent surgery (2 abscess drainage, 4 debridements and posterior stabilizations, 1 debridement and stabilization through double approach, 2 posterior approach mininvasive stabilizations).

The remaining 23 cases were treated conservatively, with drugs and no load bearing. Among the case series 24 patients healed, 8 patients are still in treatment.

**Discussion** Systemic antibiotic treatment is the main answer to spine infections. $F^{18}$ FDG PET/CT seems to be an effective method to help in diagnosis and confirm treatment efficacy. Minimally invasive surgery allows a rapid return to patient's daily life in selected cases. Conventional surgery shows its efficacy in improving the action of drugs through stability. Instrumentation does not worsen the risk of recurrence if associated with appropriate drug therapy and debridement.

**Conclusions** SIMP algorithm is a model of multidisciplinary management of complex diseases as spine infection are, in order to improve and favour relationships between specialties, targeted at helping patient and community health.

### Dynamic stabilization in the treatment of degenerative lumbar spine: mid-term results and complications

M. Bochicchio*, G. Bakaloudis, S. Astolfi

U.O.C di Chirurgia Vertebrale, San Carlo di Nancy-IDI (Rome, IT)

**Introduction** The purpose of this study was to evaluate the results of the dynamic and hybrid stabilization in the surgical treatment of degenerative lumbar spine.

**Materials and methods** From May 2009 to April 2010, 92 patients affected by degenerative disc disease underwent dynamic or hybrid stabilization (Flex-Plus). There were 51 women and 41 men; average age was 45.2 (27–74 years). 12 patients with degenerative disc disease underwent dynamic stabilization at 1 level, 12 patients at 2 levels, 32 patients affected by spinal stenosis underwent dynamic stabilization at 2 levels with decompression. Hybrid constructs (TLIF + DS) in 36 patients affected by multisegmental instability (4 patients affected by degenerative scoliosis). In 26 patients a revision procedure was performed. Follow-up was intended at time points being 3, 6 and 12 months after surgery. Clinical outcomes were evaluated using ODI, VAS, and SF-36 questionnaires. Radiologic evaluation included whole spine AP/lateral, lumbar neutral, flexion, and extension X-ray.

**Results** Clinical improvement was found to be good; VAS-Back value of 6.6 in the pre-surgery reduced at 3.2 post-surgery, VAS-Legs values were 7.1 pre and 1.4 post. The ODI average value at follow-up term was 11% comparing to the pre-surgery value of 52%. Improvement of SF-36 was fair: from 53.4 to 29.67 post.

**Discussion** The Flex-Plus system may obtain the best range of motion of the lumbar spine, prevent adjacent disc disease and restore the correct spine alignment. The hybrid construct of the Flex-Plus system consents to combine arthrodesis and dynamic stabilization.

**Conclusions** Although preliminary, our results are encouraging. The Flex-Plus system demonstrated very useful in the surgical treatment of lumbar degenerative disease not only in the pure dynamic construct but also in cases protection of the adjacent segment is also a concern.

### Venous thromboembolism after spinal surgery

C. Meloni*, A. Zachos, A. Ruggiu, F. Muresu, C. Doria, P. Tranquilli Leali

Clinica Ortopedica, Università di Sassari (Sassari, IT)

**Introduction** All the cascade mechanisms from the coagulation factor X leading to the synthesis of soluble fibrin are well known and essential in the process of haemostasis.

**Materials and methods** We carried out a prospective study to assess the incidence of deep vein thrombosis and pulmonary embolism in 72 patients undergoing spinal surgery from September 2007 to March 2008. In this study we recorded the changes of fibrin monomer complex (CMF) and D-dimer (DD) in the postoperative period for early diagnosis of venous thromboemblism frameworks. The following districts were recognised: cervical, thoracic and lumbar in 21, 16 and 35 cases respectively. The surgical approach was anterior in 3 cases, posterior in 68 cases and combined in 1 case. The average blood loss was 450 ml. In all cases a mechanical prophylaxis with elastic compression of PTEV air was performed. In no case anticoagulants prophylaxis was performed. The CMF and DD were evaluated at six different times. The Doppler ultrasound of the legs and perfusion lung scan were performed in all patients 7–10 days after surgery. Patients with suspected deep vein thrombosis and/or pulmonary embolism underwent high-definition pulmonary CT.

**Results** A PTEV was observed in 6 patients. Three patients had a picture of pulmonary embolism. Five patients had a framework of deep vein thrombosis. In 2 patients concomitantly both complications. In all 6 patients in the first post-operative Jonathan dosage of CMF appeared superior to 5.5 g/ml and was statistically significant for PTEV, while the dosage of DD showed a statistically significant increase in peak equal to 12.2 g/ml only on the seventh day.

**Discussion** It is now possible to identify coagulation factors which may have a predictive role in early diagnosis of venous thromboembolic disease (PTEV).

**Conclusions** The prevalence of PTEV after spinal surgery in our study was on average of 8.3% with other published data. The dosage of CMF compared to the DD allows the early detection of a pre-thrombotic state and therefore can be expressed as a predictor for PTEV.

### PEEK rod in lumbar fixation: preliminary experience

G. Bosco*, G.B. Scimeca, M. Cappuccio, S. Paderni, L. Mirabile, F. De Iure

UOSD Chirurgia Vertebrale, Ospedale Maggiore (Bologna, IT)

**Introduction** In past years polyetheretherketone (PEEK) has been used extensively as biomaterial in spinal surgery. Recently, rods made of PEEK have been developed for lumbar instrumentation. Theoretical advantages of PEEK rods are stress reduction at bone-screw interface and a better load sharing in the anterior column. Purpose of this work is the revision of our preliminary experience with PEEK rod implants for lumbar arthrodesis in degenerative diseases.

**Materials and methods** Between October 2008 and January 2011, 11 patients with symptomatic degenerative diseases were treated in our institution with lumbar posterior fixation with PEEK rod systems. There were seven males and four females, mean age was 64 years (min. 37–max. 82). Three of them had previous surgery (2 discectomies, 1 X-stop). All patients had posterior decompression. In seven patients, who had laminectomy and artrectomy, interbody fusion was performed. Mean follow-up was 12 months (min. 6–max. 26).

**Results** In the whole series partial or complete remission of preoperative pain was observed. One of them referred contralateral sciatic pain in early post-operative time, patient had foraminotomy with progressive pain resolution; this complication is not to refer to specific implant used. In the remaining cases no early or late complications were observed. All cases followed over than 12 months showed complete bone fusion at imaging evaluation.

**Discussion** Biomechanical studies demonstrate that PEEK rods give adequate rigidity and resistance to stress. Characteristic of PEEK as biomaterial is a elasticity module similar to bone. Semi-rigid instrumentation, although can't completely prevent its occurrence, could reduce the incidence of junctional disease. PEEK, more flexible than titanium, leads to lower loads to posterior structures with stress reduction at screw-bone interface and better load sharing on the anterior column, this promotes the interbody fusion in cases of arthrodesis. Polyetheretherketone radiolucency allows a better evaluation of fusion on conventional radiography and less artifacts with CT and MRI.
**Conclusions** Our experience has shown good results in cases treated. The semi-rigid systems can now be considered a viable option in the lumbar degenerative disease, although clinical evaluations are necessary in the longer term.

### *C14—SPINE 2*

### Percutaneous mini-invasive fixation for thoracic and lumbar fractures

M. Cappuccio*, G. Scimeca, S. Paderni, L. Mirabile, G. Bosco, E. Serchi, F. De Iure

Ortopedia e Traumatologia, Chirurgia del Rachide, Ospedale Maggiore (Bologna, IT)

**Introduction** Amielic thoracic and lumbar fractures can be treated either surgically or conservatively depending mainly on the type of fracture, but also on the needs and wills of the patient. Mini-invasive fixation is a valid option alternative to either conservative or vertebral arthrodesis, applicable to patients with politrauma who cannot stand a major surgical procedure. We present mid and long term results of patients affected by amielic vertebral thoracic and lumbar fractures treated with percutaneous vertebral fixation, and analyse the data in comparison with other procedures, conservative and open surgery.
**Materials and methods** Between May 2005 and May 2010, 133 vertebral fractures were treated (total 101 patients): 67 were male and 34 were female; mean age 47 year-old (min. 15–max. 82); 84 patients reported only spine fractures from trauma, 17 had politrauma with avarage Injury Severity Score (ISS) 25.2 (min. 17–max. 34). Thoracic-lumbar passage was the main location (T12-L1). All fractures were classified according the Magerls AO classification system: mostly type A (A1 e A3) fractures, type B and C were seldom treated with a percutaneous fixation. The main surgical procedure was a monosegmental percutaneous fixation (one level above and one below the fractured vertebral body); this procedure was performed in 79 cases. Plurisegmental fixation was achieved in 22 cases. Globally 462 pedicle screws were implanted.
**Results** Mean duration of surgical procedure was 116 min (range 35–240 min), strictly depending on the number of treated levels and consequently the number of implanted screws. For a monosegmental procedure (4 pedicle screws) the average duration of the procedure was 106 min, 144 min for 6 screws and 171 min for 8 screws. All patients affected by only spine trauma were ambulatory at POD 2 and discharged at POD 5. Politrauma patients could lie in bed in different positions the day after surgical fixation. Mean Follow-up was 29 months (range 6–64 months).
**Conclusions** Percutaneous vertebral fixation is a valid option in the treatment of type A amielic thoraco-lumbar fractures, in comparison to conservative treatment and open surgical fixation + arthodesis. Our data demonstrated good clinical outcome and complication rates similar to those of other surgical techniques.

### Spine surgery and polytrauma: damage control, orthopaedic procedure and role of minimal invasive fixation

M. Cappuccio*, S. Paderni, G. Scimeca, L. Mirabile, G. Bosco, F. De Iure

Ortopedia e Traumatologia, Chirurgia del Rachide, Ospedale Maggiore (Bologna, IT)

**Introduction** The concepts of Damage Control Orthopedics (DCO) in the management of polytrauma patients are well-known. The pivotal rules are aimed at an immediate stabilization of fractures, limiting blood loss to a minimum. Treatment strategies have been clarified in long bone fractures and in pelvic fractures, with only a few aspects remaining controversial such as intervention time. On the contrary, in polytrauma patients with spine fractures it is not yet clear the best treatment option.
**Materials and methods** From May 2005 to April 2008, 17 spine fractures were treated in 12 patients (5 female, 7 male). Their mean age was 45.8 years (range 24–65). The mechanism of trauma was a car accident in 79 patients, a fall from a height $> 5$ m in 3 (in 1 case as attempted suicide). The mean Injury Severity Score (ISS) was 25.2 (range 17–34).
**Results** All cases were followed-up after surgery by clinical and radiological examinations, the mean follow-up time being 31 months (range 24–52). All patients were immediately mobilized in different positions in intensive care Unit. No cases had sensitive or motor neurological troubles in the post-surgical period, and no infections were observed. All fractures were considered healed at the 6-month follow-up, and the complete reconstruction of vertebral found.
**Conclusions** The minimal invasive, percutaneous treatment of amyelic and stable fractures of the thoracic and lumbar spine represents a valid option, alternative to non-operative or open treatment using the traditional technique. Our experience showed excellent clinical-radiological results, without major complications. In PolyGram patients this technique should become the treatment of choice, because it conjugates the advantages of "damage control" (early stabilization and minimum blood loss) with those of "early total care" (definitive stabilization). Treatment is minimally invasive and well-tolerated by patients; it is followed by an early mobilization and easier nursing, and at the same time it proves to be the one leading to the final healing of fractures.

### Hybrid instrumental vertebral stabilization; evaluation of 40 cases treated from 2000 to 2011

S. Colangeli*, A. Gasbarrini, G. Barbanti Brodano, S. Bandiera, S. Terzi, L. Boriani, A. Corghi, R. Ghermandi, L. Babbi, S. Boriani

Istituto Ortopedico Rizzoli (Bologna, IT)

**Introduction** The strumented spinal fusion is currently the most appropriate treatment in lumbar degenerative spine disease in particular when the conservative treatment is not available. Recently a system that permits to avoid the rigid arthrodesis and the related-complications was created. It is acknowledged that a spine segment rigid fixation accelerates and increases the nearby segments degenerative processes. The use of dynamical systems was designed with the aim to achieve a "repair" or better protection of the treated segment. In the last 20 years hybrid systems have finally been designed in order to obtain the benefits of both potential surgical technique.

**Materials and methods** We analyzed 40 patients surgically treated by various kind of hybrid stabilization from 2000 to 2011 and the results of clinical terms: perception of pain by VAS and Oswestry Disability Index (ODI) before and after surgery with a follow-up period of 20 months (min. 5–max. 60) and instrumental evaluation by MRI of the intervertebral discs "protected" under the Pfirrmann classification.
**Results** Preliminary results show that the hybrid system obtained a good control of symptoms and limitated complications.
**Discussion** Spine surgery techniques to treat degenerative disease and spinal instability are basically two: the rigid fixation (traditional arthrodesis) and dynamic stabilization. The first is reserved to spinal macro instability treatment. In micro instability and where in movement preservation was necessary, elastic stabilization is performed. In recent years the possibility to use a hybrid technique with new instruments gives the surgeon the possibility to preserve or abolish the movement of each functional spinal unit according to the disease.
**Conclusions** Hybrid systems represent an alternative possibility to the traditional arthrodesis or dynamic stabilization for the treatment of degenerative spinal disease.

### Emisacrectomy: experience in 13 cases

G. Gargiulo*, N. Ruggieri, A. Solini

A.O.U. S. Giovanni Battista Molinette (Turin, IT)

**Introduction** Surgery is indicated in sacral tumours in a small number of radio or chemo-resistant tumours or in association to adjuvant therapy. When the first sacral vertebra is not involved, emisacrectomy could be the treatment of choice. We report our experience on 13 cases (9 chordoma, 1 ependymoma, 1 monostotic bladder metastasis, 2 colon metastasis) discussing surgical strategies, level of osteotomy, oncologic and functional results and observed complications.
**Materials and methods** Between 1998 and 2005, 13 patients (7 male, 6 female) aged 61 (57–73) were operated on complaining of back pain, sciatica in 2 cases, constipation in 7 cases. X-rays, CT and NRM planned the level of osteotomy, angiography was performed in bulky tumours, embolization in the first case operated where a postembolization syndrome occurred delaying surgery. Diagnosis was confirmed by CT pre-op guided biopsy. Resection was performed at S1–S2 level in 9 cases, S2–S3 in 2 cases trough a combined approach (anterior-posterior), S3–S4 in 2 cases through a posterior approach removing "en bloc" the mass from behind in all cases. Reconstruction was achieved with accurate and progressive muscular suture using a posterior mash in the first case operated. The first case developed the necrosis of the posterior wall of the rectum and required revision surgery. Three patients had posterior central wound dehiscence closed in 1 month. Hospital stay was 31 days (15–45). Sparing S1 all patients at discharge walked with crutches.
**Results** At 5-year follow-up (range 5–9), all patients were ambulant, using crutches in 3 cases, referring occasional back pain in 7 with no difference irrespective of the level of osteotomy. No fractures of the sacrum or lumbosacral instability or perineal visceral hernias was detected. Sphincter activity was normal when S3 was spared, 3 cases of chordoma required revision for local recurrence. Five patients died with median overall survival of 4 years (range 2–6), 2 chordoma for visceral metastasis, 2 for primitive tumours, 1 chordoma for other general causes.
**Discussion** It concerns the possibility of performing an intentional transgression to oncological principles for functional purpose in emisacrectomy. In our experience spine and ileolumbosacral junction remained stable, sparing S3 roots preserved sphincter function, osteomuscular edges must be wide in chordomas to avoid local recurrence, we do not recommend pre-operative embolization.
**Conclusions** Emisacrectomy is a demanding surgery, preserves pelvic stability and deambulation and sphincter activity according to the level of osteotomy.

### The lumbar-iliac stabilization in spino-pelvic dissociation

G. Bosco*[1], G.B. Scimeca[1], M. Cappuccio[1], S. Paderni[1], L. Mirabile[1], R. Pascarella[2], F. De Iure[1]

[1]UOSD Chirurgia Vertebrale, Ospedale Maggiore (Bologna, IT);
[2]UO Ortopedia e Traumatologia, Ospedale Maggiore (Bologna, IT)

**Introduction** The spino-pelvic dissociation is an infrequent condition associated with high-energy trauma. The complexity of possible patterns, sometimes associated with neurological damage, makes management of these patients very difficult even for expert surgeons. The objective of this study is the evaluation of results and complications observed in patients treated with lumbo-pelvic reduction and stabilization.
**Materials and methods** Between November 2002 and January 2010 at our Institution in five politrauma patients was performed lumbo-pelvic stabilization. All the cases were involved in high-energy trauma injuries. There were two male and three female, average age was 37 years (minimum 18 years–maximum 54 years). Two patients had peripheral neurologic deficit at presentation, for which extensive decompressive laminectomy was performed at the same lumbo-iliac stabilization. In three cases it was associated with a surgical time earlier for the synthesis and stabilization of pelvic ring injuries.
**Results** There were no major complications following surgery in any of the patients treated. In patients with mielic lesions specific rehabilitation protocol was performed with partial recovery of standing and walking with aids.
**Discussion** The rare incidence of clinical spinal pelvic dissociation and the paucity of cases described in literature, means that currently we cannot identify a gold standard for treatment of this condition. In our experience, the lumbo-pelvic stabilization has proved an effective method of restoring stability in the lower back and pelvis, allowing the quick mobilization and improved management of comorbidity in patients. A good neurological recovery was observed in the absence of root injury.
**Conclusions** The lumbo-iliac stabilization procedures are a viable option in the treatment of spino-pelvic dissociation, although it must be the recognition of more extensive clinical experience to better define the most appropriate therapeutic choices.

### *C15—SPINE 3*

### Posterolateral instrumented fusion (PLF) versus posterior lumbar interbody fusion (PLIF) in the treatment of low-grade adult isthmic spondylolisthesis

F. Lolli*, G. Barbanti Bròdano, M. Di Silvestre, K. Martikos, F. Vommaro, E. Maredi, T. Greggi

Istituto Ortopedico Rizzoli (Bologna, IT)

**Introduction** Several studies in the literature analyzed clinical and radiological outcomes of different fusion techniques in the treatment of adult low grade isthmic spondylolisthesis, including posterolateral fusion (PLF) and posterior lumbar interbody fusion (PLIF). However, considerable controversies regarding what is the "gold standard" approach still exist.

**Materials and methods** A retrospective analysis of all adult patients surgically treated at Our Institution between 2003 and 2005 for a low grade isthmic spondylolisthesis (Meyerding grade I-II) was conducted. Exclusion criteria were: etiology other than isthmic (degenerative, traumatic,…), age < 40 years, Meyerding grade > 2, previous spine surgery. 114 consecutive patients were included, and were divided in 2 groups, according to the surgical treatment they received: *PLIF Group* (posterior lumbar interbody fusion) and *PLF Group* (posterolateral fusion). Results were analyzed through the evaluation of the clinical (by filling ODI, RMDQ and VAS questionnaires) and radiological outcomes.

**Results** At a mean follow-up of 62 months, 71 patients, 28 of *PLIF Gro*up and 43 of *PLF Gro*up, were completely reviewed. The two groups were well matched according to patient age, spondylolisthesis grade and level, fusion extension. ODI, RMDQ and VAS questionnaires did not show statistically significative differences between the two groups in terms of clinical outcome. Fusion rate was 97% in *PLIF Group*, 95% in *PLF Group*. Major complications (requiring revision surgery) occurred in 5 out of 71 patients reviewed (7%), 1 in *PLIF Group* (3.6%) and 4 in *PLF Group* (9.3%). Pseudoarthrosis occurred in 1 case in *PLIF Group*, in 2 cases in *PLF Group*.

**Discussion** Our results did not show a real advantage resulting from posterior lumbar interbody fusion in terms of stability and fusion in the treatment of adult low grade isthmic spondylolisthesis. Both techniques ensured good clinical results, without statistically significative differences.

### Surgical treatment of spondylodiscitis with and without the use of spinal instrumentation

A. Ruggiu*, C. Meloni, A. Zachos, F. Muresu, C. Doria, P. Tranquilli Leali

Clinica Ortopedica, Università degli Studi di Sassari (Sassari, IT)

**Introduction** Retrospective analysis of the results of cases of spondylodiscitis treated surgically with and without the use of spinal instrumentation.

**Materials and methods** 29 patients with spondylodiscitis were subjected to surgical treatment over the past 20 years. The results of 14 patients (*group 1*) treated by debridement and instrumentation were compared with those of 15 patients (*group 2*) treated by debridement alone. A comparison of clinical and sagittal angle was performed. The average age was 57 years and the follow-up was 8.4 years. In 17 cases the causative agent was *Staphylococcus piogenes* while in the remaining 12 cases it was a specific form of tuberculosis. Most patients had pain with spinal neurological signs of commitment. In most cases the infection covered the district thoracolumbar. In about one half of the patients of *group 1* a double anterior and posterior surgical approach was performed while all patients in *group 2* were operated only through the anterior surgical approach.

**Results** The clinical results in both groups overlapped with a value of VAS (Visual Analogic Scale) pre-operative equal to 9.54 and 9.57 respectively in group 1and group 2 while a postoperative value of 2.33 in group 1 and 2.4 in group 2 was achieved. This figure, however, appears to deteriorate in the clinical follow-up time at greater distances especially in group 2 and this is probably related to the worsening of the deformity that is greater in group 2 (mean sagittal angle 7.7°) than in group 1 (mean sagittal angle 1.8°).

**Discussion** The treatment of spondylodiscitis is mostly conservative with the use of orthoses and targeted antibiotic therapy. Surgical treatment is indicated in cases in which neurological signs of commitment, severe pain, abscesses ossifluenti, instability and kyphosis with destruction of limiting somatic spine. The use of spinal instrumentation does not increase the incidence of recurrence of infection but rather stabilizes the affected segment and maintain proper sagittal plane.

**Conclusions** The surgical tool of spondylodiscitis is recommended in locations thoraco-lumbar kyphotic deformity and in multiple locations.

### The fusion instrumented with percutaneous systems: longitude, sextant and interbody cage for the treatment of the lumbar degenerative spondylolisthesis

F. Muresu*, A. Zachos, A. Ruggiu, C. Doria, C. Meloni, P. Tranquilli Leali

Clinica Ortopedica, Università degli Studi di Sassari (Sassari, IT)

**Introduction** Degenerative disc disease is due to the alteration of biomechanical and biochemical properties of the intervertebral disc that occurs with age and is facilitated by repetitive microtrauma. The disc degeneration process cannot be attributed simply to particular lifestyles or work activities, it is thought that in the pathogenesis of degeneration, the genetic predisposition plays a fundamental role, and this is demonstrated by the fact that there are frequent cases of very young subjects with diffuse disc degeneration, cervical and lumbar.

**Materials and methods** The aim of this study was to evaluate the long-term results of lumbar interbody fusion technique combined with posterior percutaneous vertebral stabilization. The interbody arthrodesis in degenerative intervertebral disc demonstrates still valid despite the advent of biological prosthetic procedures. From 2006 to present degenerative spondylolisthesis were treated by 95 circumferential arthrodesis (sextant and longitude) with interbody cage away by PLIF, tlif, axialif. The symptoms presented were back pain, neurogenic claudication, mono/pluriradicolapatie paintings and mixed. All patients had undergone conservative treatment for a period not less than 6 months. All operations were performed by the same surgeon, with an average time of 100 min, contained blood loss (340 cc). Patients had a follow-up period of 12 months (range 3–24) and controls were evaluated at 1, 3, 6, 12, 24 months.

**Results** The amount of pain as assessed by VAS and ODI showed a significant decrease. There was no implant failure. We have not reported perioperative complications.

**Discussion** Due to the high popularity of low back pain various therapeutic approaches were developed, from those represented by the conservative medical and physical therapy to surgery. Among the latter, the fusion has been considered for many years the treatment of choice for discogenic low back pain since the genesis of the pain was attributed to a change in the kinetics of the affected segment, consequently, the abolition of the movement resulted in a resolution of symptoms.

**Conclusions** The use of minimally invasive surgery has proved a valuable tool allowing the surgeon to achieve optimal fusion with fewer complications compared to traditional "open".

### Posterior transforaminal instrumented interbody fusion (TLIF): comparing open and minimally invasive surgery

G. Barbanti Bròdano*[1], K. Martikos[2], F. Lolli[2], A. Gasbarrini[1], S. Bandiera[1], S. Colangeli[1], L. Boriani[1], A. Corghi[1], T. Greggi[2], S. Boriani[1]

[1]Chirurgia Vertebrale Oncologica e Degenerativa, IOR (Bologna, IT); [2]Chirurgia delle Deformità del Rachide, IOR (Bologna, IT)

**Introduction** Interbody instrumentation in the degenerative lumbar pathology is an efficient treatment in terms of bone fusion and clinical outcome, reaching satisfying results in more than 90% of cases. Various studies have highlighted the advantages of minimally invasive and percutaneous techniques because of the lesser surgical damage upon spinal soft tissues, but their efficacy in comparison to the open surgical procedures is still controversial.
**Materials and methods** We performed a retrospective analysis of prospectively collected data of 30 consecutive patients (*Group 1*), affected by lumbar disc disease and treated by posterior percutaneous pedicle screw instrumentation and transforaminal instrumented interbody fusion (TLIF). A comparison was made with another group of 30 consecutive patients, presenting the same pathology, who received the same type of instrumentation with an open surgical procedure (*Group 2*). All patients were treated during the time period between 2006 and 2009, and there were no statistically significant differences in terms of demographic characteristics. Mean age of all patients was 52 years (min. 36–max. 61). Clinical evaluation was performed using VAS, ODI and SF-36 questionnaires at 2 and 6 months, and at 1 year. Radiographic evaluation was performed upon standard X-rays of the lumbar spine at the same time intervals. Differences were evaluated by using the Student *t*-test.
**Results** We obtained a statistically significant improvement of patients' clinical status, both post-operatively and at final follow up, in *Group 1* (preoperatory vs. postoperatory data respectively): lumbar VAS 7.1 versus 3.2, leg VAS 6.8 versus 2.6, ODI 68% versus 32%, SF-36 tot. 32% versus 83%. Also in *Group 2* respective differences reached a similar statistically significant difference, while we did not register significant differences between the two groups. Mean hospital stay after operation was of 4 days in *Group 1* (min. 3–max. 7) and of 6 days in *Group 2* (min. 5–max. 11) ($p < 0.05$). Mean intraoperatory blood loss was 150 ml in *Group 1* and 650 ml in *Group 2* ($p < 0.01$). Intraoperatory complications rate was 2.1% in *Group 1* and 2.4% in *Group 2* ($p > 0.5$). At final follow-up radiographic evaluation showed satisfying fusion rates in both groups.
**Conclusions** Minimally invasive interbody transforaminal fusion is a secure and efficient surgical procedure and, when successfully performed, can reach desired results in similar rates to the respective traditional open surgical procedures. Limited surgical damage upon muscles and soft tissues, restrained blood loss and shorter hospitalization represent the major advantages of minimally invasive techniques.

### Percutaneous fixation versus open arthrodesis in the treatment of thoraco-lumbar passage: short-term results and comparison

M. Cappuccio*, G. Scimeca, S. Paderni, L. Mirabile, G. Bosco, E. Serchi, F. De Iure

Ortopedia e Traumatologia, Chirurgia del Rachide, Ospedale Maggiore (Bologna, IT)

**Introduction** Amielic type A thoracic and lumbar fractures can be treated surgically or conservatively depending mainly on the type of fracture, but also to the needs and wills of the patient. In recent years mini-invasive surgical techniques were born aiming shorter surgical procedures and faster recovery and ambulation. The objective of this study is direct comparison in the immediate post-operative management of patients affected by amielic T12 or L1 fractures between percutaneous fixation (group A) and open arthrodesis (group B).
**Materials and methods** Between January 2008 and September 2010, 50 patients were treated for amielic T12 or L2 fracture. Politrauma, pelvic fractures, long-bone fractures, more than one vertebral fracture were excluded from this analysis. Percutaneous mini-invasive fixation was performed in 29 cases, open surgery with arthrodesis was performed in 21 patients. Duration of surgery, intraoperative blood loss, radioexposure, post-operative hospitalization and the accuracy of pedicle screw positioning were analysed.
**Results** *Group A* patients were more radioexposed than *group B* patients but with a shorter hospitalization. Duration of surgical procedure, intraoperative blood loss and accuracy of screw positioning were not statistically different between the two groups. Autologous blood reinfusion or blood transfusion were not necessary in any group.
**Conclusions** Our data does not promote one technique or the other. In particular no differences in intraoperative blood loss were found.

### *C16—SPINE 4*

### Occipitocervical stabilization, a retrospective study of 21 cases

G. Gargiulo*, N. Ruggieri, A. Solini

SCDO Ortopedia e Traumatologia, AOU San Giovanni Battista Molinette (Turin, IT)

**Introduction** Occipitocervical instability is associated to diagnostic and therapeutic problems with high incidence of mortality and morbidity because of neurological deterioration requiring craniovertebral junction reduction, stabilization and fusion with direct or indirect neurological decompression. Authors present their experience discussing technical aspects and clinical results according to the device used and the pathologies treated.
**Materials and methods** During the period 1995–2008, 21 patients were operated because of occipitocervical instability (13 female, 6 male), aged 53 years (14–65) affected by different pathologies (rheumatoid arthritis 7, congenital instability 5, failed surgery 4, metastasis 4, fracture outcomes 1). All patients complained of occipitocervical pain with neurological impairment in 17 (Nurik 1 in 6, 2 in 5, 3 in 2, 4 in 1, 5 in 3). In 7 cases reduction was achieved in Halo-jacket. Ostheosynthesis was performed with sublaminar wires in 7 (Hartshill adapted rectangle in 1, Ranford loop in 6), with screws and hooks in 14 (11 CCD, 3 Summit) with fusion in all case but two (metastasis), extending distally to C4 in 2, in C5 in 9, to C6 in 6, to C7 in 4. In 7 indirect neurological decompression was performed in 10 direct. In 3 cases using sub laminar wires a dural tear without clinical consequences was observed. All patients have weared a collar for 30 days.
**Results** At a medium follow-up of 7 years (2–10) 15 patients were asymptomatic, 6 had occasional pain, discrete cervical mobility in cases extended to C6, neurological symptoms showed an overall improvement (Nurik 0 in 8, 1 in 3, 2 in 1, 3 in 2, 4 in 2, 5 in 1), fusion was evident in 13 cases. One patient was operated again because of skull screw mobilization and subsequent rupture of the bar.
**Discussion** In our experience ostheosynthesis with screws and hooks proved to be the safest technique. Fusion was not detected in imaging in all cases even with a good clinical outcome. Limiting the extension has been a satisfactory articulation of the cervical spine. Neurological outcome depended on the amount of initial neurological damage.
**Conclusions** Occipitocervical fusion is a demanding surgery which involves the sacrifice of the junction motion. An accurate technique early performed allows to obtain a satisfactory segmental realignment and a good clinical outcome with less risk.

### Comparison between vertebroplasty and kyphoplasty in the surgical treatment of thoracic-lumbar vertebral fractures due to osteoporosis

G.A. La Maida*, L.S. Giarratana, A. Acerbi, B. Misaggi

Istituto Ortopedico G. Pini (Milan, IT)

**Introduction** Vertebroplasty (VP) and kyphoplasty (KP) are minimally invasive procedures used for the treatment of thoracic-lumbar pain due to vertebral compression fractures. These vertebral augmentation methods are mainly indicated in osteoporotic pathological fractures, even if they can be sometimes employed to treat traumatic fractures and vertebral osteolytic metastasis. The main difference between the two techniques is that kyphoplasty allows the correction of the kyphotic deformity, giving advantages in terms of restoration of the sagittal vertebral balance.
**Materials and methods** Among a sample of 32 patients that underwent a surgical treatment for a vertebral osteoporotic fracture, 16 were retrospectively re-evaluated (1 year minimum follow-up); the full number of vertebrae was 37, all treated with vertebroplasty or kyphoplasty. Kyphoplasty was employed for the treatment of 26 levels (70.3%), while vertebroplasty was employed in the remaining 11 levels (29.7%). The aim of this study is the comparison between the clinical and radiographic results of the two groups, KP and VP.
**Results** All patients showed a clear improvement of pain, evaluated through the Visual Analogue Scale (VAS), already during the first post-operative day, without any difference between the two groups. The pain relief did not show any variation during the successive clinical check-ups. Radiographic results were statistically evaluated through a Student *t* Test that showed a statistically significant difference between KP and VP concerning the vertebral body height restoration and the correction of the kyphotic spine deformity. In particular, the mean post-operative vertebral body height obtained through the employment of KP was almost twofold compared to VP (2.04 mm in VP vs. 4.20 in KP); besides, KP showed an evident correction of the mean spinal kyphosis (1.69° in VP vs. 3.68° in KP).
**Discussion** Kyphoplasty gives better results in terms of vertebral body height restoration and correction of the kyphotic spinal deformity; these data, combined with a lower risk of extra-vertebral cement leakage during kyphoplasty, make this technique more effective than vertebroplasty, in particular in the case of multiple fractures of the thoracic-lumbar passage.
**Conclusions** In summary, it is possible to affirm that both surgical methods, VP and KP, can treat in a similar way the focal back pain due to vertebral osteoporotic fractures, with a clear difference in favour of KP, which offers clear advantages in terms of vertebral body height restoration and correction of the spinal deformity.

### The lumbar spinal fusion: what access?

A. Ruggiu*, F. Muresu, A. Zachos, C. Meloni, C. Doria, P. Tranquilli Leali

Clinica Ortopedica, Università degli Studi di Sassari (Sassari, IT)

**Introduction** For the surgical treatment of low-back pain the techniques of intersomatic instrumented arthrodesis have been suggested as an alternative to posterolateral fusion. The interbody arthrodesis has some biomechanical advantages and results in higher success rates of arthrodesis compared with posterolateral techniques but are associated with a higher incidence of complications, particularly in combined approaches (360°). The use of combined approaches (front and rear) for lumbar arthrodesis is not recommended as routine in all cases but should be used only if there is deficiency of the anterior column deformity.
**Materials and methods** The pre-operative study uses a series of imaging techniques: Standard X-rays (AP + LL); dynamics and oblique right and left X-rays, elettroneurofisiological study, MRI, CT for the study of neural foramen. The adoption of evaluation forms (SF-36, Oswestry, Spine Tango) is necessary for the objective analysis of outcomes. Currently, there are a variety of techniques for interbody arthrodesis: ALIF (anterior lumbar interbody fusion); PLIF (posterior lumbar interbody fusion), TLIF (transforaminal lumbar interbody fusion); Xlif (extreme lateral interbody fusion); AxiaLIF (axial lumbar interbody fusion); Olif (oblique lumbar interbody fusion). The technique differs according to surgical access. Such access can be: front, rear, transforaminal, or extreme lateral trans-sacral, but in any case you will need to carefully clean the disc, cruentation vertebral plates, the positioning of any bone graft + cage. In selected cases it is possible to associate a summary trans-pedicular posterior mini-invasive.
**Results** The inspection took place in the short, medium and long-term clinical and radiographic follow-up (standard X-rays) at 1, 6, 12 and 36 months with completion of evaluation forms (SF-36, Oswestry and Spine Tango); CT 12 months for evaluation process arthrodesis.
**Discussion** The procedures for spinal surgery may provide an anterior approach, posterior, transforaminal, or extreme lateral trans-sacral.
**Conclusions** The use of minimally invasive surgery while requiring a good knowledge of anatomy and proper surgical spinal vision imaging has proved a valuable tool allowing the surgeon to achieve optimal fusion with fewer complications than traditional techniques “open”.

### Circumferential lumbar fusion with percutaneous minimally invasive approach using rods in titanium and peek: preliminary clinical and radiological results

A. Zachos*, C. Meloni, A. Ruggiu, F. Muresu, C. Doria, P. Tranquilli Leali

Clinica Ortopedica, Unversità degli Studi di Sassari (Sassari, IT)

**Introduction** Arthrodesis circumferential lumbar fusion is the combination of a front and a rear. The anterior arthrodesis is achieved by means of an interbody cage placed through a minimally invasive approach. The posterior arthrodesis is achieved instead using pedicle screws and rods inserted percutaneously. In spinal surgery have been introduced new instruments with different biomechanical properties, the peek is the most promising.
**Materials and methods** The aim of our study was to evaluate clinical and radiological fusion obtained using different types of rods, PEEK and titanium, while in all patients, the interbody fusion cage that has been used was in peek. From November 2007 to December 2010, twenty-four patients underwent to circumferential lumbar fusion. The mean age was 45.5 years. The levels involved were L3–L4 in 4 patients, L4–L5 in nine patients and L5–S1 in 11 patients. In all cases had been diagnosed spondylolisthesis. In 10 patients have been used rods in peek and in the remaining 14 patients titanium rods. For the evaluation of low back pain were used scales (VAS) and Oswestry (ODI). The merger was evaluated by postoperative plain radiographs at 1, 3 and 6 months, while the CT was performed at 3 and 6 months after surgery.

**Results** The mean follow-up was 14 months. The clinical results were satisfactory in all patients without significant differences between the two groups. It was observed an improvement in VAS and ODI. X-rays and CT scans performed at 3 months after surgery show better signs of fusion in the group of patients treated with rods peek compared to those treated with titanium rods.
**Discussion** Preliminary results suggest that lumbar segments stabilized with peek rods have a load response similar to that with less physiological stress on adjacent levels.
**Conclusions** The lower hardness of the rods in peek than titanium allows a better clearance of the anterior column and promotes faster interbody fusion compared with installations that include the use of rods in titanium.

### Our experience in surgery of cervical disc prosthesis: preliminary results in the medium term

C. Meloni*, C. Doria, A. Ruggiu, A. Zachos, F. Muresu, P. Tranquilli Leali

Clinica Ortopedica, Università di Sassari (Sassari, IT)

**Introduction** The degenerative cervical intervertebral disc consists of the gradual disappearance of the mechanical properties: the absorption of static and dynamic loads and intervertebral motion. In the presence of clinical symptoms and neurological spinal does not resolve with conservative treatment, the therapeutic response is surgical and has been for many years the discectomy followed by the fusion, however, such treatment can lead over time to a functional overload of the adjacent segments with the ability to manifest, in time, disc disease overload. The prosthetic intervertebral disc replacement allowed us to address this possibility. Prostheses preserve motion between vertebral bodies, but do not absorb the static—dynamic, which affect the cervical spine.
**Materials and methods** Our Centre started the experience of cervical prosthetic surgery in 2008 using the prosthesis Discocerv-Scienti'X in 17 cases. The preoperative diagnosis was a herniated disc in 9 cases and 6 cases symptomatic degenerative disc disease. The average age of patients was 48 years (range 43–59) levels were treated C4–C5 in 6 cases, C5–C6 in 8 cases and C6–C7 in 3 All patients were assessed pre-operatively using clinical scales at points (ODI and VAS) and by standard X-rays in projections, dynamic CT and MRI. All patients underwent periodic clinical and radiographic evaluation every 6 months to 2 years after surgery.
**Results** The mean follow-up was 14 months (range 60–30). All patients reported a marked improvement in clinical symptoms confirmed by a significant reduction in VAS and ODI values than pre-operative evaluation. In no case we found radiographic signs of loosening of the prosthetic implant and/or overload conditions in the adjacent intervertebral discs.
**Discussion** In our experience, albeit limited in our series, we confirmed the validity of this method in the right indications.
**Conclusions** The presence of vertebral instability, and a framework of advanced arthritis and uncoartrosi interapophyseal represent absolute contraindications to implant prosthetic cervical disc, such surgery is not difficult but requires some learning curve and must represent the weapon in luggage cultural spinal surgeon.

### Combined posterior and anterior arthrodesis by PLIF in degenerative lumbar spine

C. Faldini*, F. Acri, M.T. Miscione, R. Chehrassan, P. Capra, G. Tedesco, C. Galante, S. Giannini

Clinica Ortopedica, Istituto Ortopedico Rizzoli, Università di Bologna (Bologna, IT)

**Introduction** The lombosciatalgia of degenerative dorsal column is a common pathologic problem that is frequently seen in clinics. It is manifested during elderly and it is usually due to an asymmetrical reduction in intervertebral spaces in association with lateral deviation of spine and segmental or central foraminal stenosis. Sometimes appear with vertebral instability that could lead to worse clinical symptoms. The aim of this study is to show the clinical results of 30 patients whom were operated for lombosciatalgia following degenerative spine with combined posterior and anterior arthrodesis by PLIF.
**Materials and methods** 30 patients affected by lombosciatalgia with degenerative spine were operated from 1997 to 2010. The surgical interventions were planned by considering these parameters: extension of deformity, presence of central foraminal stenosis, pain and peripheral neurological symptoms and instability. The deformities were involved in 1 level in 4 cases, 2 levels in 11 cases, 3 or more levels in 15 cases. Surgical treatment consisted of hemilaminectomy and for amination with stabilization and restoration of intervertebral spaces by PLIF. Preoperative pain according to VAS was 7.7 + 3.
**Results** At the last control (4.2 + 3 years), pain according to VAS was 2.9 + 2; 18 cases presented with excellent results, 7 with good and 5 with moderate.
**Discussion** The degenerative spine presents with a series of deformities that appears gradually and progressively and for this reason it is well tolerate. The patients affected by lombosciatalgia following degenerative spine however usually show local pain limited to one or few intervertebral spaces. Extensive stabilization is not indicated based on the age of patients and particularly based on the necessity to treat the main pathology. Therefore relief of pain will be achieved by surgical treatment of these spaces, that are usually instable, with combined anterior and posterior arthrodesis.
**Conclusions** The surgical treatment of lombosciatalgia of degenerative spine with combined posterior and anterior arthrodesis by PLIF was effective and when every aspect of the pathology evaluate accurately and is decided to treat surgically, this technique could be the most appropriate option for each type of deformity.

### *C17—HIP 1*

### Revision strategies following breakage of ceramic components in the THR

P. Dalla Pria*[1], D. Masoni[1], P. Esopi[2], L. Zagra[3]

[1]Samo S.P.A. (Bologna, IT);
[2]U.O.C. di Ortopedia e Traumatologia (Dolo-Venezia, IT);
[3]Istituto Ortopedico Galeazzi IRCCS (Milan, IT)

**Introduction** Ceramics is widely used for heads and liners because of its wear resistance and high biocompatibility. Nowadays CoC large heads couplings are available and the dislocation rate has been reduced. However, potential risks of edge loading can produce squeaking and liner breakage. When ceramic debris are spread in the joint different revision strategies can be considered.
**Materials and methods** The microseparation of the femoral head and the fracture of the liner is explained. In case of ceramic breakage different revision strategies are compared: metal on polyethylene, ceramic on polyethylene, ceramic on ceramic. In vitro tests were performed to simulate the effect of residual ceramic debris (third body wear) using Biolox Delta® heads and UHMWPE liners. After 5 millions of cycles the weight loss of Biolox Delta® heads was measured and the UHMWPE liners surface was observed.

**Results** The heads weight loss was 2–4 mg with a minimal damage of the surface. On the surface of the UHMWPE liners many ceramic fragments were found. The fragments caused no macroscopic damage of the joint surface.
**Discussion** The experimental evidence demonstrated that ceramic debris is not risky for the UHMWPE liners.
**Conclusions** In case of revision due to breakage of ceramic components the use of a new UHMWPE liner and a new Biolox Delta® head seems to be an adequate solution. In the clinical experience no cases of failures caused by either a new ceramic on UHMWPE or ceramic on ceramic couplings are reported. On the opposite, severe metallosis can occur if metal heads are used.

### MIS versus traditional approaches for total hip replacement: a review

D. Lazzara*, A. Petrini

SC Ortopedia e Traumatologia, Ospedale San Giovanni di Dio (Florence, IT)

**Introduction** The various approaches proposed for the mini-invasive total hip arthroplasty can be roughly divided into a small incision approaches (usually "evolution" of the traditional one) or two incisions. In this meta-analysis, we aimed to compare clinical and radiological results (both short and long term) and complication rates in case series of total hip arthroplasty (THA) implanted by using traditional access or performed by minimally invasive approach, analyzing the latest, authoritative literature on the subject. Reviews concerning economic, as well as clinical factors were also taken into consideration for the two types of interventions.
**Materials and methods** We performed a systematic review of all articles published on the topic (Medline, CINAHL, Amed, EMBASE, PubMed) until January 2011. A comparison of clinical and radiological results of THA applied by minimally invasive techniques with those of traditionally implanted THA, paying particular attention to the differentiation of results in relation to the length of follow-up. The cost and type of complications reported by various authors were also carefully evaluated.
**Results** The results reported in the literature tend to favour the minimally invasive technique for what concerns the speed of functional recovery, blood loss, cost (for a little better) than to the traditional, although, in general, the results become fully comparable after 1 year of follow-up, with the same incidence of complications such as aseptic failure and revision of components, postoperative periprosthetic fractures, appearance of heterotopic ossification, dislocations.
**Conclusions** Based on extensive review of recent literature on the comparison between traditional THA and minimally invasive implanted THA, it can be concluded that in most series, the results after the first year after surgery are comparable. The minimally invasive technique offers obvious advantages in the perioperative period and immediately post-operatively.

### Restoration of femoral offset after total hip artrhoplasty

T. Binda*, F. D'Angelo, M. Rossi, P. Cherubino

Clinica Ortopedica, Università dell'Insubria (Varese, IT)

**Introduction** The restoration of the normal and bio-mechanic features of a hip is essential for the successful outcome of a prosthesis. An important factor is the restoration of femoral offset that influences the lever arm of the abductors, muscle strength and the dynamics of step. The aim of this study was to evaluate the femoral offset restoration after total hip replacement.
**Materials and methods** During the period between January 2008 and December 2009, 261 patients underwent surgery for total hip replacement. The diagnosis was in 229 cases (88%) of primary coxarthrosis, 21 (8%) avascular necrosis of the femoral head, 8 post-traumatic coxarthrosis and 3 secondary to other diseases. An uncemented prosthesis was used In 166 patients (64%) and a cemented stem in 95 (36%). The clinical evaluation was performed by Harris Hip Scoring system. The radiographic evaluation was performed in order to study: neck-shaft angle, femoral offset, center of rotation, the lever arm of the abductors, and tilt version of the cup.
**Results** At a mean follow-up of 16 months (range 9–24) the average score of the board of Harris was 92.7 (range 69–100) in uncemented implants while it was 88.8 (range 71–100) in cemented prosthesis. In the group of cementless stem the radiographic evaluation showed in 12% of cases (20 patients) a similar offset between the pre-and post-operative X-rays, in 48% (80 cases) it was reduced (range 2–19 mm) and in 40% (66 patients) it was increased (range 1–12 mm). In the group of cemented stem the radiographic evaluation showed in 9% (8 patients) a similar value between the pre-and post-operative X-rays, in 18% (17 patients) was reduced (range 3–26 mm) in 73% (70 patients) it was increased (range 1–19 mm).
**Discussion** The limits of this study are related to the probable under-estimation of the offset value (about 3–5 mm) measured on plain radiographs in AP due to the impossibility of assessing the femoral anteversion. Finally, the short follow-up makes difficult to understand if an inadequate offset's restoration may affect the longevity of the implant. The average value of the offset reached by our study is in line with the average values calculated in the literature, this value justifies the good clinical results.

### Hip arthroplasty for failed treatment of proximal femoral fractures

A. Carcangiu*, D. Perugia, A. Speranza, R. Alonzo, S. De Sanctis

II Facoltà di Medicina e Chirurgia, Azienda Ospedaliera S. Andrea, Università di Roma "Sapienza" (Rome, IT)

**Introduction** Failed treatment of an intertrochanteric fracture typically leads to profound functional disability and pain. Salvage treatment with hip arthroplasty is one of surgical options. The purpose of this study was to evaluate the results and complications of hip arthroplasty performed as a salvage procedure after the failed treatment of an intertrochanteric hip fracture.
**Materials and methods** Twenty-one patients (sixteen women and five men) with a mean age of 75.8 years (range 61–85 years) were treated in our hospital with hip arthroplasty for failed treatment of interthrochanteric hip fracture. In 19 of 21 patients we performed a total hip arthroplasty. In 2 out of 21 cases we used a bipolar hemiarthroplasty.
**Results** In all cases we obtained good clinical results, with a relatively low number of complications. A statistically significant improvement was found comparing pre and postoperative condition ($p < 0.05$).
**Discussion** The most appropriate treatment for these fractures remains controversial. Infact both reduction and internal fixation and replacement arthroplasty have been advocated as the primary treatment for these fractures.
**Conclusions** Our experience confirms that total hip arthroplasty is a satisfactory salvage procedure after the failed treatment of an intertrochanteric fracture in elderly patients with few serious orthopaedic complications and acceptable clinical outcomes.

### The use of Allofit cup in total hip replacement

F.R. Evola*, L. Costarella, G. Sessa

Clinica Ortopedica dell'Università di Catania (Catania, IT)

**Introduction** The Allofit is a hemispherical cementless cup of pure titanium, which demonstrates excellent primary stability and allows all existing tribological couplings. The aim of this study was to evaluate the cups Allofit implanted at the Orthopedic Clinic of Catania from June 2003 to December 2010.

**Materials and methods** 325 operations were performed using the allofit cup: in 223 using the multi-hole cups and in 102 the non-holes cups. The initial diagnosis was coxarthrosis in 192 cases, the medial fracture of the neck in 48, dysplasia of the hip in 38, aseptic necrosis in 23, coxa protruse in the 9, the outcome of lateral fractures in 9, and arthritis in 6. Patients were 182 women and 108 men, the mean age was 55 years (range 34–76). The affected limb was left in 121 cases, the right in 134 and bilaterally in 35. The implants were followed both clinically, using the Merlè-D'Aubigne-Postel scale (0–18 points), and through the conventional radiology with serial control at 3, 6 and12 months and every year.

**Results** The follow-up was 4.2 years (range 3 months–7.5 years). The clinical and radiographic examinations performed at regular intervals have shown satisfactory results. Clinical results show a mean score that increases from 7 to 15 points. Radiographically there were no signs of loosening or migration of implants or periprosthetic osteolysis, with only 10 cases with non-progressive radiolucent lines <2 mm at 1° and 2° acetabular zone of De Lye.

**Discussion** The use of this cup promotes a limited removal of bone with preservation of the sub-chondral bone, the transmission of the physiological loading forces, the use of large diameter heads, a high bone-implant contact and greater stability to the forces of rotation, thanks to its surface structure Ridgelock®, consisting of micro wedges to "hook". These advantages reflected on improved biological fixation of implant to the bone, thus avoiding the development of events of stress shielding or concentration of load forces.

**Conclusions** The Allofit cup in hip replacement proved to be as a reliable and safe material, with high modularity and versatility. Despite the availability of modern and effective materials facilitates the activities of the orthopaedic surgeon, only the reconstruction of the biomechanics associated with a correct surgical technique are essential principles in order to achieve greater longevity of the prosthetic implant.

### *C18—HIP 2*

### Hip resurfacing prostheses: results over the medium term

F. Volpato*[1], G. Bove[2], G. Bove[2], R. Venanzi[1]

[1]Istituto Neurotraumatologico Italiano (Grottaferrata-Rome, IT);
[2]Università Sapienza Roma (Rome, IT)

**Introduction** A hip resurfacing prosthesis is now considered a valid alternative to traditional hip replacement implants, especially for patients under the age of 60. One of the most important benefits of this prosthesis is the conservation of the femoral neck (bone stock): the quality of the bone in this area is therefore the object of our study, because the risk of fracture may lead to the failure of the operation.

**Materials and methods** Our study was carried out on 40 patients whose operations have been performed since April 2008. The implanted prosthesis in all cases was MITCH RESURFACING HIP STRYKER. The operations were performed by the same surgeon using the same point of insertion (Bauer). The bone mineral density (BMD) was measured before and after the operations using the DEXA (Hologic QDR 4,500 W) method, subdividing the femoral neck area into four regions of interest (ROI). Post-operatively patients were examined after 6, 12, 18 weeks, 6 months, and 1 year.

**Results** The analysis of the densitometric data, when compared with the pre-operative data which showed no risk of fracture, indicated a reduction in bone density in the first 3 months after the operation. Thereafter followed a progressive increase in bone density, a stabilization was achieved at the 1 year mark. These data coincides with the conclusions of Cooke, N et al. 2009, Kishida et al. 2004, and Harty et al. 2004. After 12–18 weeks BMD begins to increase again thanks to the normal revival of the modelling-remodelling function, an indicator of complete vascularisation.

**Conclusions** In order to reduce the risk of fracture in the resurfacing prostheses, it is important to evaluate the bone density of the femoral neck. Patients who should not undergo this operation are those at risk of fracture of the femoral neck (including women over 55, men over 60), as well as those with osteoarthritis. The reduction in BMD in the first 12–18 weeks after the operation was consistently noted in all of the patients and does not constitute grounds for increased post-operative risk given its temporary nature. It is, however, advisable to undergo either adequate antiresorption or osteoinductive therapy and to take further precautions so as to prevent the risk of fracture.

**Suggested reading**

1. Cooke N, Rodgers L, Raewlings D, McCaskie A, Holland J (2009) Bone density of the femoral neck following Birmingham hip resurfacing. A 2 year prospective study in 27 hips. Acta Orthopaedica 80:660–665.

### Short stem and circumferential support: essential conditions for the validity of a mechanical and biological hip prosthesis

A. Foti*[1], S. Barreca[1], G. Maccauro[2], G. Epasto[3], M. Ferlazzo[4], M.A. Rosa[1]

[1]Sezione di Ortopedia e Traumatologia, Dipartimento Specialità Chirurgiche, Università degli Studi di Messina (Messina, IT);
[2]Dipartimento Scienze Ortopediche e Traumatologiche, Università Cattolica Roma (Rome, IT);
[3]Dipartimento di Chimica Industriale e Ing. dei Materiali, Università degli Studi di Messina (Messina, IT);
[4]Casa di Cure Ortopediche e Traumatologiche (Messina, IT)

**Introduction** In Hip Replacement Surgery small femoral stem prostheses generally classified as "short stems" are actually inspired to different biomechanical principles. The short stem prosthesis was designed and built with the intention to obtain a physiologic distribution of forces acting on the hip and at the same time to achieve a minimally invasiveness on bone stock and in soft tissues.

**Materials and methods** The study refers to 74 ultra-short implants, characterized by the absence of the diaphyseal shaft and the presence of a lateral shoulder and a better preservation of the femoral neck. The 74 implants refer to 67 patients including 31 men and 36 women, the implant was bilateral in 7 patients, 4 men and 3 women. The surgical approach was in 61 cases anterolateral, in 4 cases postero-lateral and in 9 cases anterior. The concerning period of the study is between June 2006 and January 2010, the remote control is from a minimum of 1 month to a maximum of 50 months. The age is between 31 and 82 years.

**Results** We evaluated the results obtained in 74 implants through X-rays examinations and 'Harris Hip Score' executed before, after the operation and every 6 months for the 1st year and every 1 year for the following 3 years.

**Discussion** The use of stems of small sizes, with reduced contact with the femoral bone, reopens the debate on the biomechanics of the hip. In the use of short stems, a non-compliance and therefore a distribution of biomechanical forces, that are not adequately supported by the shape of the short stem, causes a significant risk of adverse periprosthetic bone remodeling and mechanical failure early. The stem, built according to the dynamic biomechanical models that provide for the distribution of the load in compression on the medial and lateral proximal femur, according to the authors, is the fundamental condition for the best results.

**Conclusions** It is estimated the positioning of the stems, considering the complications of varus and valgus, but in the current state of the follow-up they were clinically well tolerated.

### Femoral neck preservation in THA

G. Zanotti*, M. Paganelli

U.O. Ortopedia e Traumatologia, Azienda USL di Ravenna (Lugo di Romagna, IT)

Increasing functional requests in even younger THA patients led to a continuing evolution towards less invasive surgical approaches, new surface finishing, new tribological coupling and new prosthetic design. The concept of tissue sparing surgery by preservation of the femoral neck using a very short calcar loading stem is to preserve bone stock and allow a high activity level. This stem design provides stability by fixation through the lateral cortex of the neck; preservation of the bone stock by retaining the femoral neck and most of the metaphyseal cancellous bone; primary fixation of the stem by the neck cortex and impacted metaphyseal bone; a physiologic load transfer along the trabecular systems, distributing the stress towards the medial and lateral diaphysis; and elasticity of the bone-prosthesis system by containing most of the stem within the metaphyseal cancellous bone to create a module (bone-prosthesis) with variable and integrated elasticity. Purpose of this paper is to present our experience in use of Parva femoral neck preserving stem with pourous titanium surface finishing, metaphyseal fixation and modular neck. A unique feature of this stem is the ability to address leght and lateral offset with modular necks, that means enhanced restoration of different morphologies of the femur, in a wide range of hip anatomic patterns. This leads to better biomechanical joint reconstruction and less invasive surgical approach, reduction in blood loss and higher functional outcomes.

**Suggested readings**

1. Pipino F, Calderale PM (1987) Biodynamic total hip prosthesis. Ital J Orthop Traumatol 13:289–297.
2. Pipino F, Molfetta L (1993) Femoral neck preservation in total hip re-placement. Ital J Orthop Traumatol 19:5–12.
3. Pipino F, Molfetta L, Grandizio M (2000) Preservation of the femoral neck in hip arthroplasty: results of a 13–17 years follow-up. J Orthopaed Traumatol 1:31–39.
4. Falez F, Casella F, Panegrossi G, Favetti F, Barresi C (2008) Perspectives on metaphyseal conservative stems. J Orthopaed Traumatol (2008) 9:49–54; doi:10.1007/s10195-008-0105-4.
5. Cameron H, McTighe T, Woodgate I et al (2010) A new approach to neck sparing THA stem. J Bone & Joint Surg—British Volume 92-B(Suppl 1):105.

### Multiple revisions in total hip replacement

F.R. Evola*, L. Costarella, V. Pavone, G. Sessa

Clinica Ortopedica dell'Università di Catania (Catania, IT)

**Introduction** The revision of a total hip replacement is often more difficult and challenging, with often less brilliant and unexpected results. Subsequent revisions to the primary are defined multiple, and are characterized by an increase of mechanical and biological problems that arise after repeated surgical procedures. The purpose of this paper is to review the main causes of multiple revision and to present therapeutic options of the international literature, and the case study of the Orthopaedic Clinic of Catania.

**Materials and methods** From January 1996 to December 2010, 121 hip revisions were performed. The multiple revisions were 12, whose 10 secondary and 2 tertiary. The mean age of patients was 76 years (range 72–88). The cause that led to the revision of the plant was aseptic loosening. The cup was revised in seven patients, the stem in one, both in four cases. The affected limb was the right one on 5 cases and the left one on 7 cases. Acetabular and femoral bone defect was evaluated with GIR classification of bone defects. The implants were followed both clinically, using the Merlè-D'Aubigne-Postel scale (0–18 points), and through the conventional radiology with serial control at 3, 6 and12 months and every year.

**Results** The mean follow-up was 6 years (range 2–8 years). The clinical and radiographic examinations performed at regular intervals showed satisfactory results. Clinical results showed a mean score that increased from 4 to 12 points. Radiographically there were no signs of loosening or migration of implants. The grafts used showed no signs of resorption.

**Discussion** The number of revisions performed in a patient contributes significantly in reducing the amount of available bone for osseointegration and to degenerate gradually peri-articular soft tissues, which play a crucial role in maintaining the stability of the system over time. The result of multiple revisions is closely related to the cause of the first revision, the interval of time between revision surgery and the compromise of joint kinetics.

**Conclusions** The availability of several methods of treatment in prosthetic surgery facilitates the work of the orthopedic surgeon, especially in those cases more complex and difficult to resolve. The best treatment is always an early diagnosis, because it avoids the progression of bone loss and allows a reconstruction of biomechanics nearer to physiological ones.

### The neck-preserving hip arthroplasty (CFP) through anterior approach: our experience

P. Di Benedetto*, V. Cainero, R. Gisonni, A. Beltrame, F. Mancuso, A. Causero

Clinica Ortopedica, Azienda Ospedaliero-Universitaria di Udine (Udine, IT)

**Background** Hip arthroplasty is evolving to minimally invasive surgery. CFP arthroplasty is a bone-preserving alternative for hip replacement in young patients. In addition, the anterior approach allows tissue sparing, utilising intermuscolar and internervous planes.

**Materials and methods** From September 2007 to September 2009, 46 patients affected by primary arthritic hip underwent CFP replacement through anterior approach. The clinical and radiographic follow-up was at 6–12, 24 months post surgery.
**Results** Pain control and hip function were improved. At 1-year of follow-up no radiological loosing was evident.
**Conclusions** CFP arthroplasty through anterior approach is a minimally invasive procedure thanks to tissue-sparing. CFP has a short femoral neck preserving stem with very proximal metaphyseal anchoring that saves the bone stock if a future revision is needed. The anterior approach improves post-operative rehabilitation.

### Stress shielding: analysis of prosthetic materials and their interactions in hip replacements (10 years follow-up)

F. Pipino*, M. Cimmino

Policlinico di Monza (Monza, IT)

**Introduction** Stress shielding is undoubtedly amongst the most important causes that limit the life of replacements. The latter are highly influenced by the materials used and the interaction between femoral and acetabular components.
**Materials and methods** The materials examined are titanium, ceramic (aluminium based) and polyetilen (PE). The combinations are ceramic-PE and ceramic–ceramic. Combination metal-PE and metal–metal will not be considered in the report. The analysis examines 62 replacements 10 years after their implantation. The evaluation focuses on two phenomena, osteolysis and remodelling, and it predicts their duration and evolution. The replacements taken into consideration are cementless and of two prosthesys kinds: Antega and the CFP (neck preservation). Concerning Antega, we compared the combination ceramic–ceramic and ceramic-PE. The combination for the CFP replacements analysed, instead, was only based on ceramic-PE.
In all replacements examined the diameter of the head was 28 mm—stem and shell in titanium with double coating (pure titanium/hydrossiapatite). In all cases the results were outstanding: no symptom even after 10 years. Further surgical intervention was not needed. The criteria were employed for the evaluation: (1) SIBOT-HARRIS score; (2) RX in AP + axial and, (3) in some cases DEXA.
**Results** The results are reported in details with an accurate analysis of osteolysis and remodelling. As a whole, for many of them a further excellent duration, that depends on the interaction bone-replacement, be easily predicted. For other cases, the evolution suggests the possibility of an aseptic osteolysis, not always correlated to good clinical conditions.
**Discussion** The distribution of the solicitations reshapes the periprosthetic bone and influences significantly the duration and results of the replacement. In the worst scenarios, the most important and frequent problem is due to stress shielding, namely the slipping of loads around the replacement that triggers on one side atrophy and on the other hypertrophy and sclerosis.
**Conclusions** In conclusion, we confronted the outcomes of replacements in regard to the combination hard-soft (ce-pe) and hard–hard (ce–ce) in order to evaluate stress-shielding and the remodelling. The best results of the the analysis in the long-term, more than 10 years, are obtained with the combination ceramic-polytetilene, namely the gold standard. There are no significant differences as far as the stress shielding is concerned, whilst the peri-prosthetic remodelling is better with the combination ceramic-pe.

### *C19—HIP 3*

### Osteonecrosis of the femoral head, decompression and graft with a new bone substitute: pro-dense

R. Civinini, F. Matassi, L. Nistri*, S. Puccini, B. Redl, M. Innocenti

CTO Firenze (Florence, IT)

**Introduction** The most used surgical procedure in the early stages of the osteonecrosis is represented by the decompression of the femoral head with bone graft or metallic devices to prevent or delay the collapse of the femoral head. The aim of our study is to establish the preliminary results with the use of a composite injectable bone substitute, made by a 75% of calcium sulphate and a 25% of calcium phosphate (Pro-Dense®), as a mechanical supplementation associated with decompression procedure in treating early stages of the osteonecrosis of the femoral head.
**Materials and methods** 18 surgical procedures of decompression associated with use of Pro-Dense© to treat osteonecrosis of the femoral head were performed, between February 2009 and February 2010, at the Author's institution. According to Steinberg's classification, 5 patients were classified as grade I, 9 as grade II and 4 as grade III. We performed a clinical evaluation of the patients using the Harris Hip Score and a x-ray evaluation at 1, 2, 3, 6 months and at 1 year from the surgical procedure. Magnetic resonance was performed after 1 year from the procedure.
**Results** One patient with an osteonecrosis classified as Steinberg's grade III had permanence of pain and worsening functional limitation. At the X-ray evaluation we noticed collapse of the femoral head and after 8 months from the decompression we performed a total hip arthroplasty with resolution of the pain. All the other patients showed no collapse of the femoral head at the X-ray evaluation. Sphericity of the femoral head was maintained with no appearance of deformity or suchondral collapse. Moreover, the synthetic bone was remodeled with new deposition of trabecular bone. At final follow-up the mean Harris Hip Score increased from 39 points pre-operatively to 82 points post-operatively.
**Discussion** Decompression of the femoral head associated with various procedure of filling the bone defect is an universally recognized procedure as effective in pain reduction and progression delay of the osteonecrosis of the femoral head. Our preliminary analysis performed in 18 patients shows the efficacy of our procedure in pain relief and X-ray progression delay of the disease. Our best indications to perform this procedure are the patients with symptomatic osteonecrosis of the femoral head in a pre-collapse stage. However this procedure can be widened, in particular situations, to patients with more advanced stage of the osteonecrosis with good results even at long term.

### Treatment of hip osteonecrosis with regenerative medicine and minimally invasive surgical technique

T. Frisoni*, D.M. Donati, L. Cevolani, S. Giannini

Istituto Ortopedico Rizzoli (Bologna, IT)

**Introduction** Avascular osteonecrosis of the femoral head is a disease that predominantly affects subjects between 20 and 40 years. When it is not a consequence of trauma, it is associated with steroid use, alcoholism, storage diseases, coagulopathies, hematologic and autoimmune diseases, but a considerable fraction of osteonecrosis was idiopathic. If left untreated, it leads to the collapse of the femoral head with severe functional limitation and early progression to osteoarthritis. There are several possible approaches with mixed results.

**Materials and methods** Since September 2008 we have treated 27 osteonecrosis in 26 patients (18 male, 8 female) mean age 35 years (min. 17–max. 55) with core decompression of the necrotic area by transtrochanteric approach in minimally invasive surgery and application of concentrated autologous bone marrow, autologous platelet gel and demineralized bone matrix. In 7 patients the necrosis was idiopathic, in 16 high-dose steroid use related, post traumatic in three patients, alcohol abuse in one patient. We used the Ficat classification to stage the degree of necrosis: in 16 cases it was found to be stage II (9 cases, IIa; 7 cases, IIb), while in the remaining 11 cases necrosis was stage III-IV (8 cases III, 3 cases IV). The outcome was assessed using Harris Hip Score (HHS), X-rays and MRI in 45 days, 3, 6, 12 and 24 months and the treatment was considered failed if a prosthetic replacement was necessary.

**Results** The average follow-up was 6 months (min. 45 days–max. 24 months). The HHS has shown an increase (from 58 to 89.5); the patients with Ficat stage II of the necrosis have shown a better clinical response (from 61 to 96.5) than patients with stage III–IV (54–82.5). In two cases the treatment failed.

**Discussion** Local conditions that lead to osteonecrosis require a treatment that stimulates tissue regeneration while preserving the integrity of anatomical structures. The rationale of our method is to improve the local regenerative microenvironment, providing the stimulus (decompression and growth factors) and osteoblastic precursors, with a minimally invasive technique which does not affect the joint vascularization, leading to clinical and radiographic good results.

**Conclusions** Core decompression of the necrotic area associated with application of concentrated autologous bone marrow, autologous platelet gel and demineralized bone matrix is a good alternative to other salvage therapies to prevent femoral head prosthetic replacement. The clinical and radiographic results are satisfactory and promising, although they must be considered preliminary.

### Treatment of fractures of the lateral neck femur in elderly: our first experience with a new anatomical nail

F. Pallotta[1], M. Cianfanelli*[1], F.R. Rossetti[1], U. Bianco[1], F Lamponi[2]

[1]Ospedale S. Camillo (Rome, IT);
[2]Azienda Ospedaliera (Rimini, IT)

**Introduction** There is a continuous progress in the study and development of new materials in the treatment of the lateral femural neck fractures, both stable and unstable. A new generation titanium anodized, anatomical intramedullary nail has recently been developed: the cervical-medullary Zimmer Natural Nail (Zimmer, Warsaw, Indiana, US). It presents a lateral offset of only 4° to facilitate trochanteric access. It is present in two versions, short and long, different diameters and various degrees of neck-shaft angle are available to better match the individual anatomy of the patient. The diaphysal part has spiral grooves. The system includes a screw cephalic LAG to 10.5 mm diameter with threaded end in TMT (Trabecular Metal Technology).

**Materials and methods** From August 2010 to date we have been treating 154 subjects over 65 years (min. 65–max. 96), of which 58 men and 96 women suffering from fracture of the lateral femur neck. All patients were classified according to the AO/OTA classification. The time elapsed between the arrival at the hospital and surgery was 80% of cases of 24–72 h. The average time of surgery was 45 min (20–70 min). Physical rehabilitation was started immediately post-operatively and partial load was granted, when the conditions of the patient allowed during the first 3 day.

**Results** The intraoperative complications were 2 cases of error in position of the screw cephalic and 1 case of shaft fracture in a patient with a very narrow shaft. The follow-up was a 1, 3 and 6 months and it was possible to control only 40% of patients and this is due to the increased mortality linked to old age and to the difficulty to introduce controls subsequent outpatient due to health issues and social these patients. All fractures healed on average 45 days. The late complications were 1 case of cut-out (due to an incorrect positioning of the cephalic screws), 2 cases of wound infection healed without additional surgery.

**Conclusions** In conclusion, although the casuistics is still small, we discuss the best treatment of lateral fractures of the femur neck, stable and unstable, in elderly. ZNN nailing allows to perform a surgical access of only 3–4 cm with little blood loss and, thanks to the anatomical shape, it is able to guarantee good stability, allow immediate mobilization and rehabilitation start for a rapid functional recovery.

### Femoral osteotomy combined with a PTA computer navigation

M. Citi*

ICOT (Latina, IT)

Anatomical pathological changes of the hip joint are multiple. There are significant variations between the articular orientation and the relationships of the anatomical landmarks associated with leg length discrepancy in post traumatic and dysplastic diseases. The goal of the hip replacement is to reconstruct the joint to obtain hip stability with optimal orientation of the components because the long term results are linked with the biomechanical conditions. The aim is to restore the normal articular relationships in post-traumatic hip and in dysplasia even with asymmetric hip using the classical surgical technique with femoral osteotomy.

We use and describe, in pathological anomalies of the hip, the computer assisted surgical navigation procedure, step by step, to obtain the optimal and secured stable positioning of the conventional acetabular cup and the standard stem. This procedure reduces the mismatch of the component orientation in the positioning of the arthroplasty and control the initial or secondary leg length discrepancy. The ultimate goal is to obtain the functional range of motion without internal rotation of the foot during the gait and a reconstructed well orientated abduction mechanism to avoid limp.

### Minimally invasive anterior approach for hip prosthesis in the treatment of fractures of the femur neck

M. Trono*, M. Gigli, G. Lucidi

Ospedale Infermi Rimini (Rimini, IT)

**Introduction** Minimally invasive anterior approach is a real step forward in the mini-invasive story of the hip prosthesis. The preservation of muscle–tendon insertions guarantees a better and faster recovery with a lower rate of complications. We applied this new technique for prosthetic treatment of fractures of the femur neck.
**Materials and methods** From March 2010 to January 2011 we applied minimally invasive anterior approach to 48 fractures of the femoral neck (28 bipolar prostheses, 20 hip arthroplasties).
**Results** From the analysis of the results we observed less post-operative pain and blood loss, reduced need for transfusions, reduced hospitalization, reduced use of crutches, compared to the traditional approaches used in our Division (posterolateral and direct lateral approach). As complications we observed a higher rate of fractures of the greater trochanter, especially at the beginning of the experience.
**Discussion** The anterior approach to the hip is, in our opinion, the most innovative mini-invasive approach. The access does not sacrifice any muscular insertion, based on the principle of muscle-splitting. In fact, it crosses the gap between the group of sartorius/rectus femoris and tensor fascia lata. The exposure is adequate, although one should practice some technical tips (capsulotomy rear, change of position of the lower limbs) to expose the femoral canal in the right direction and to prevent complications. In our experience we do not use bed traction, the operative field is single, we use only uncemented anatomical stems. The complications observed at the beginning of the experience are thereafter considerably reduced.
**Conclusions** The results of this work show that the benefits of minimally invasive anterior approach may also useful in the treatment of femoral neck fractures, as well as in degenerative disease. We believe that the faster functional recovery and reduced risk of complications is useful not only in the adult patient, but also in elderly where these features facilitate the recovery and survival in trauma.

### *C20—HIP 4*

### Massive acetabular transplantation in hip revisions with severe bone defects: 21-year experience

V. Bellotti*, R. Ledesma, D. Marinó, M. Ribas, J. M. Vilarrubias

Instituto Universitario Dexeus (Barcellona, Spain)

**Introduction** The reconstruction of complex acetabular defects in the revision surgery is a matter of discussion without a general consent. We describe the experience of our Department in the last 21 years with the use of crioconserved acetabular graft in type Gross III and IV acetabular defects.
**Materials and methods** The study group is represented by 44 reviewed cases on a total of 67 massive transplantations performed during prosthetic revision hip surgery, with a mean follow-up of 14.2 years (range 10–21). According to Gross Classification, 26 cases were Type III, while 18 Type IV. Patients were evaluated through the Merle D'Aubigne score; The radiographic evaluation of the transplantations and acetabular cups was performed according to the Engh Criteria (JBJS 1994).
**Results** In 42 transplantations (95.4%) the radiological homogenization of the trabecular pattern was observed. In 43 cases (97.2%) a cementless cup was implanted. We observed 3 cases (6.8%) of infection, and 8 cases (18.1%) of aseptic loosening of the acetabular component, that required a new revision. According to the Kaplan Meyer's curve, the global survival was of 80.5%, with a 76.4% rate in cases of Gross III. In case of pelvic discontinuity (type Gross IV), the rate results are significantly higher (85.7%, $p = 0.018$). An improvement of the Merle D'Aubigne Score was observed both in the walking function and in the pain parameters.
**Discussion** Previous reports in literature describe a high incidence of massive acetabular graft failure at mid term. Our data show that massive acetabualr allograft transplantation represents a surgical technique able to restore bone stock and satisfactory results at mid-/long-term follow-up. A correct graft selection and a customized preparation as a correct fixation technique seem to be key factors for the results.
**Conclusions** In our experience the massive acetabular transplantation represents a surgical technique able to give satisfactory clinical and structural results in hip revisions with complex acetabular defects at mid- and long-term follow-up.

### Hip arthroscopy learning curve

V. Bellotti*, J.I. Erquicia, M. Tey

Instituto Universitario Dexeus (Barcellona, Spain)

**Introduction** Hip arthroscopy is a procedure that is living a new expansion, but few authors have discussed the importance of the learning curve [1]. The purpose of our perspective study was to correlate the learning curve with the complications in hip arthroscopic surgery.
**Materials and methods** In the period between January 2006 and March 2009, 97 hip arthroscopies were performed by a single senior surgeon. The series have been separated into two groups: the *group A*, composed by the first 30 procedures, and the *group B*, composed by the following 67. The demographic and clinical pre-intervention data (surgical indication, surgical technique, surgical time) such as intra- and post-operating complications have been picked in a perspective way. Patients were evaluated through the WOMAC score. According to the complications described in literature, the attention is set on the observation of the following: pudendal, sciatic and femoral nerve apraxia, cutaneous ulcers, dislocation or sub dislocation of the hip, fracture of the femoral neck, abdominal compartment syndrome, pulmonary thrombus-embolism.
**Results** *Group A*: 5 complications (16.6%) were observed, all represented by transitory pudendal apraxia. *Group B*: 2 complications (2.9%) were observed: a condral lesion of 4 mm and a labral due to a trans-labrum portal.
**Discussion** Complications decreased with the number of performed interventions as the data present in literature; the most frequent complications were not severe, and mostly predictable. Unexpected events can increase the global incidence of complications, deviating the incidence from the literature data.
**Conclusions** Hip arthroscopy is a difficult technique, with long surgical time and a long learning curve. In our opinion a devoted instrumentation and a tight following of the procedure guidelines are fundamental to correctly perform a hip arthroscopy and decrease the number of complications.

**Reference**

1. Lubowitz JH, Poehling GG (2009) Who Among Us Should Perform Arthroscopic Surgery of the Hip? Arthroscopy 25:335–336.

### Hip prosthesis of acetabular fractures with double mobility cup in elderly patients

C. Battiato*, A. Ciuffoletti, A. Mulap Muteb

ASL 13, ASUR Marche (Ascoli Piceno, IT)

**Introduction** Acetabular fractures in elderly people pose a treating problem, secondary to gravity and to the postoperative period, that will change according to the surgical technique chosen. The need of reducing the time of entrapment and accelerate recovery of the load induced us to apply the principles proposed by Dott. Helfet in the AHSS. We expose the results obtained in 5 patients affected by acetabular fracture, treated with reduction, internal fixation and application of a hip prosthesis, with a double mobility cup.
**Materials and methods** Five patients, affected by acetabular fracture, characterized by comminution, impacted fragments or important osteoarthritis, were operated of internal fixation of the fracture and application of a hip prosthesis with double mobility cup. In all patients, operation was performed through a Kocher–Langenbeck approach; once fixation of the main fragments had been obtained we proceeded on positioning the double mobility cup and the femoral stem.
**Results** These results were clinically evaluated using the Harris hip score and X-rays.
**Discussion** In consideration of the advanced biological or chronological age of the patients, we aimed a better functional recovery, immediate, rather than a better preservation of the anatomy of the region. Although reduction and internal fixation of articular fractures is the main objective for surgeons, advanced age of our patients induced us to a more pragmatic attitude. Some of the treated fractures would have needed a double surgical approach for an optimal reduction or a delayed weight bearing in case the surgical option chosen was only osteosynthesis. The risk of avascular necrosis of the femoral head is elevated in these patients and often these hips are painful and affected by osteoarthritis before the trauma. Helfet proposed a single step treatment of these fractures to accelerate timing of recovery and we followed this principle, gaining good functional results. The cup applied was a double motility one I order to reduce postoperative luxations but also to reduce mechanical stresses exercised by the prosthesis on the fixation devices.
**Conclusions** The treatment of complex, articular, acetabular fractures in elderly people can be managed with internal fixation and application of a hip prosthesis in one step. This decision lengthens surgical time but allows the patient to a more rapid functional recovery.

### GIR grade I and II acetabular revisions with Porous or Trabecular Titanium acetabular cups, with or without acetabular screws

G. Sacchetti*[1], F. Munari[1], C. Tsatsis[1], S. Bondioli[1], F. Pinto[1], F. Catani[1]

(Modena, IT)

**Introduction** In grade I e II acetabular revisions according GIR classification (Italian Society for Revision Arthroplasty), with mostly cavitary and single wall defects, today's tendency is to use hemispherical cups with or without screws for acetabular fixation. Trabecular or Porous Titanium or Trabecular Metal (Tantalum) surface coating may improve primary fixation and secondary bone ingrowth. In our experience the use of acetabular cup with these new coatings can allow acetabular revisions with cups usually implanted in primary hip prostheses.
**Materials and methods** We started using the Delta TT Trabecular Titanium Acetabular Component and the Zimmer Trabecular Metal Modular Acetabular System Since their introduction in the market in 2006, and the Fixa Ti-Por Adler acetabular cup, with or without acetabular screws, since 2009 for the acetabular revisions classified as GIR I and II defects. From 2006 to 2010, we performed 48 acetabular revisions with these cups (38 Delta TT, 8 Fixa Ti-Por and 2 Zimmer Trabecular Metal) in 155 acetabular revisions isolated or associated with stem revision.
**Results** In 39 cases, 2 or 3 acetabular screws were used, in the other cases it has been possible to carry out a press-fit cementless implantation. In all cases we have associated a banked femoral head bank allograft to overwhelm the cavity defects. We did not have any complications related to implant of the cup, especially dislocations, because these cups allow us to use large diameter ceramic femoral heads (36 and 40 mm). Tree Delta TT acetabular cups were removed for post-operative infection and after appropriate monitoring reviewed with Delta Revision with iliac screws.
**Conclusions** In our experience, use of primary hip implant acetabular cups with porous coating of titanium or tantalum, with or without acetabular screws, is a correct indication for acetabular revision with cavitary or single wall defects, thanks to the possibility to use hard-wear bearing surfaces and large diameter femoral heads and to the preservation of bone stock for any future revisions.

### Post-operative mortality related to surgical delay for hip fracture surgery

M. Marinelli*, M.G. Bugatti, A. Catalani, L. De Palma

Cattedra di Ortopedia e Traumatologia, Università Politecnica delle Marche (Ancona, IT)

**Introduction** The aim of our study is to estimate the effect of delay in surgery for hip fracture on 30-day mortality using a risk adjustment strategy to control the effect of demographic and clinical confounders.
**Materials and methods** This observational study was carried out on all patients admitted with a hip fracture and discharged between January 1995 and December 2008 from a teaching hospital. Gender, age, time to surgery, mortality and medical comorbidities were derived from hospital discharge records (SDO), while American Society of Anaesthesiologists (ASA) score was retrieved from clinical records. Backward stepwise logistic regression was used to identify potential confounders in the relationship between time to surgery and mortality. A final multivariate logistic regression analysis was carried out controlling the effect of confounders.
**Results** In the 1199 patients who underwent surgery (mean age, 65 years), the mean time to surgery was 5.07 days. In 207 (17.7%) patients the mean time to surgery was less than 48 h, while in 962 patients (82.3) it was more than 48 h. The 30-day mortality in patients with a pre-operative time of 1.57 days was 9.27%; the 30-day mortality in patients with a pre-operative time of 5.80 days was 10.44%.
**Discussion** Patients with a hip fracture should undergo surgery within 2 days from admission in order to reduce 30-day mortality.

### *C21—TRAUMATOLOGY 5*

### External fixation in complex leg reconstruction with bone, muscle and skin loss

G. Corina*[1], A. Marsilio[1], N. Tartaglia[2]

[1]U.O.C. Ortopedia, P.O. "Vito Fazzi" (Lecce, IT);
[2]U.O.C. Ortopedia, P.O. "San Paolo" (Bari, IT)

**Introduction** From January 2005 to December 2010 we have treated 16 patients affected by severe open leg fractures or post-traumatic conditions with big bone, muscle and skin loss.
**Materials and methods** Six cases were type III B and 10 were type III C fractures according to Gustilo-Anderson classification (11 males and 5 females with medium age of 47 years). All patients were treated with surgical toilette of necrotic tissue and bone. Authors used the resection-lengthening technique using Ilizarov circular frame, Sheffield frame and monolateral rail fixator. Proximal osteotomy has been made and bone has been elongated 1 mm by day. Medium follow-up has been 24 months and medium healing time with external fixator has been 7 months.

**Results** Functional results have been excellent in 6 cases and good in the remaining ones. No infections of fracture site have been observed while 4 superficial infections of wires and screws have occurred (all cases healed with oral antibiotics). No post-traumatic deformity, non union, chronic osteomyelitis, was observed. In 6 cases post-traumatic joint stiffness occurred.

**Discussion** We observed the progressive bone loss restore was associated to skin and soft tissue restore avoiding any additional plastic surgery free flaps.

**Conclusions** We describe techniques, results and complications.

### New treatment for heel articular fractures with external fixator: our experience

G. Corina*[1], A. Marsilio[1], N. Tartaglia[2]

[1]U.O.C. Ortopedia, P.O. "Vito Fazzi" (Lecce, IT);
[2]U.O.C. Ortopedia, P.O. "San Paolo" (Bari, IT)

**Introduction** Treatment of fractures of the heel is still controversial therefore several different techniques are used to treat complex fractures. It is widely described in literature that the gold-standard is open reduction and internal fixation (O.R.I.F.). Less invasive techniques are gaining increasingly attention because they minimize soft tissue complications due to open surgery as deep infections and wound healing delays. We provide notes on surgical technique, clinical cases, results and complications.

**Materials and methods** Since May 2009 to December 2010 in a multicentre study we treated 27 complex calcaneum articular fractures (in 25 patients) with Orthofix monolateral external fixator. The fractures were classified according to the Sanders fracture classification system. The Maryland Foot Score method was used to functionally evaluate the patients and X-rays and CT scans were performed at different stages of the treatment.

**Results** Over a minimum follow-up of 2 years we had evidence of excellent functional results in most cases and patients were fully satisfied. All patients healed within 3 months.

**Conclusions** We believe that external fixation is a good option to treat heel fractures minimizing complications and gaining great functional results. We describe surgical techniques, results and complications.

### Percutaneous calcaneoplasty in intrarticular calcaneal fractures: results at 2-year follow-up

F. Biggi, C. D'Antimo*, S. Di Fabio, C. Salfi, S. Trevisani

Ospedale San Martino (Belluno, IT)

**Introduction** The ideal choice of treatment for displaced intraarticular calcaneal fractures remained controversy. Open reduction and internal fixation is still the most popular surgical procedure; however, minor and major wound complications remain a major concern because of the thin and vulnerable skin over the lateral calcaneal wall, which is exposed during surgery. The aim of this study was to evaluate the results of a new surgical mini-invasive procedure: closed reduction technique combined with balloon-assisted fracture augmentation with cement (percutaneous calcaneoplasty).

**Materials and methods** We retrospectively reviewed 11 patients (7 female and 4 male) sustained displaced calcaneal fractures (2 bilateral cases) treated with percutaneous calcaneoplasty at our institution in the period from January 2008 to January 2010. Cement was used in 10 fractures and calcium phosphate in the others 3 cases because of the age of patients (less than 50 years). CT scan with three-dimensional (3D) reconstruction was performed preoperatively and 1 month after surgical operation. The average follow-up was 24 months. Time of bone healing, level of pain, satisfaction rate and peri- and post-operative complications were evaluated.

**Results** All cases progressed to bone union in an average time of 3 months. Patients were able to walk with partial weight-bearing after 7 days. Full weight-bearing was performed 1 months postoperatively. No wound complications and no cross reactions occurred. One patient complained residual minor pain in calcaneal area.

**Discussion** The percutaneous calcaneoplasty represents a viable alternative for the treatment of intraarticular, dislocated calcaneal fractures. The minimally invasive approach led to early full weight bearing, good functional patient outcomes and a low complication rate.

### Calcaneal fractures treated with external mini-fixator

V. Caiaffa*, D. Speciale, M. Sperti, L. De Paola

S.C. Ortopedia e Traumatologia, SS. Annunziata (Taranto, IT)

**Introduction** The management of intra-articular calcaneal fractures remains a challenge. "Open" and minimally invasive technique are described in various studies. Open surgery is now the "gold standard" for displaced intra-articular calcaneal fractures, because of anatomical reconstruction, but major complications related to it are often catastrophic; orthopedic surgeons are studying minimally invasive technique, even if reduction of fragments is incomplete and difficult to obtain. In patient with soft tissue damage and general and local contraindication particularly favorable results were obtained.

**Materials and methods** Between 2009 and 2011, 15 consecutive closed articular displaced calcaneal fractures in 12 patients (3 bilateral) were treated with percutaneous reduction and external fixation. Mean age was 53 years (min. 24–max. 70). The operation was performed under image intensifier control and external fixation obtained using a device from the Orthofix Mini-Fixator series (Orthofix SpA). Soft tissue damage in politrauma patients was our indication for 2 patients. In the other cases we planned the operation, choosing this technique with the aim of early mobilization, closed and indirect reduction, ease implant removal.

**Results** Reduction and alignment was satisfactory in all cases; all the fracture healed in 12 weeks (3 excellent, 6 good, 1 fair, 1 poor, 4 just in treatment, sec. Maryland Foot Score). Post-operatively weight-bearing was not allowed for 10 weeks. Passive and active mobilisation of the ankle began immediately. The fixator was removed after 12 weeks. The only one complication was a superficial infection of pins, in a patient.

**Discussion** The main goal of treatment for displaced fractures of the calcaneum should be the restoration of the three-dimensional structure. The use of an external fixator appears to achieve this aim, without anatomic reconstruction of subtalar joints. It is useful not only for extreme cases but it could be a surgical choice "ab initio". Percutaneous reduction and external fixation provide an incomplete reduction, stable fixation, and give the possibility of early mobilization.

**Conclusions** The external fixation of displaced intra-articular calcaneal fractures is a valid alternative treatment compared with open reduction and internal fixation, even if reduction of fragments is incomplete and difficult to obtain. In patient with soft tissue damage and general and local contraindication particularly favorable results were obtained.

### *C22—TRAUMATOLOGY 6*

### Surgical treatment of comminuted fracture of radial capitellum by capitellectomy

M.T. Miscione*[1], F. Acri[1], M. Nanni[2], D. Leonetti[2], C. Galante[1], M. Bonomo[1], C. Faldini[1], S. Giannini[1]

[1]Clinica Ortopedica, Istituto Ortopedico Rizzoli, Università di Bologna (Bologna, IT);
[2]Unità Operativa di Ortopedia e Traumatologia, Ospedale Maggiore "C.A. Pizzardi" (Bologna, IT)

**Introduction** The fracture of radial capitellum can be treated by ORIF, capitellectomy and prothesis. There are some complications following of capitellectomy: secondary elbow arthritis, proximal migration of radius with consequent wrist pain, valgus instability and cubital valgus, strength lost for holding the objects and radio-ulnar sinostosis. The aim of this study is to evaluate the clinical and radiographic results of 12 patients affected by isolated radial capitellum fracture that had been treated surgically by capitellectomy.
**Materials and methods** Twelve patients with isolated radial capitellum fracture underwent capitellectomy intervention. In the standard antero-posterior and lateral view radiography of elbow 7 fractures were of type II according to Mason classification and 5 fractures were of type III. We excluded fractures of type I and IV Mason classification from our study. Clinically, the residual pain of elbow and wrist, valgus deviation, elbow range of motion in flexion–extension and pronation-supination were evaluated, while the radius was graphically evaluated for secondary arthritis according to Broberg & Morrey classification (grade 0–grade 3), proximal migration of radius with distal radio-ulnar joint subluxation, para-articular calcification, radio-ulnar sinostosis and the formation of neocapitellum.
**Results** Obtained clinical results were excellent in 8 patients and good in 4. Valgus deviation was seen in 2 patients. A patient reported mild limitation in the extension of elbow and 2 patients were reporting painless moderate limitation in pronation-supination movement. In radiographic control there were signs of arthrosis of mild grade in 3 patients and moderate grade in one patient. In 2 patients proximal migration of radius with consequent articular subluxation was seen. In one patient there was a periarticular calcification. There was no evidence of radio-ulnar sinostosis or neocapitellum formation signs. The mean follow-up was 13 years.
**Discussion** The comminuted radial capitellum fracture can be treated by ORIF, prosthesis and capitellectomy. The reduction, to reduce the risk of post-operative complications, has to be anatomic but does not always reach to satisfactory results. Use of prosthesis is another complicated technique and its use is not devoid of complications related to prothesis failure. The capitellectomy is based on a rapid intervention and the low learning curve.
**Conclusions** In case of comminuted fracture of radius capitellum when anatomic reduction is not possible, the capitellectomy could be a valid surgical option with satisfactory long-term radio-clinical results.

### Treatment of politraumas: first revision of 2000–2010 cases

G.D. Marrara*, F. Lamponi

Ospedale S. Camillo Forlanini (Rome, IT)

**Introduction** Orthopaedics and Traumatology Unit of San Camillo Forlanini Hospital of Rome treated 23,000 patients in the years 2000–2010. About 500 were politraumas.
**Materials and methods** 23,000 patients treated, divided into orthopaedics and prosthetic; monodistrectual trauma and geriatrics; politrauma emergency treatment, stabilization in intensive care unit, final treatment in 1 or more steps classification of politrauma involving fracture of 1 or more limbs, associated or not with pelvic and spine fractures.
**Results** Number of treatments, hospitalization, clinical results, patient satisfaction rates, surgical techniques are described.
**Discussion** Hospital organization casuistics related to evaluation of politraumas treatment.
**Conclusions** Organization and efficiency of the different care units, and cooperation among them, are fundamental for excellent results.

### MIO with plate

A. Belluati*, E. Mariani, E. Lupetti, G. Maldini, A. Colombelli

Ospedale Santa Maria delle Croci (Ravenna, IT)

The study analyses the clinical results obtained in the treatment of the proximal humerus fractures with LCP plates and MIO technique. The objective of this study is to determine the ideal treatment looking on the fracture's personality and the soft tissues injuries. We evaluated 86 patients, treated in our Clinic from 2007 to 2010 with a mean follow-up of 10.3 months. In conclusion, we suggest a MIPO technique in the A type fractures; in the B type fractures it depends on the surgeon's experience, whereas in B3 type the ORIF approach is the gold standard; in the C type fracture, in order to reach an anatomical reduction, we suggest ORIF technique.

### Use of the demineralized bone matrix (DBM), platelet rich fibrin (PRF) and bone marrow concentrated (BMC) increase healing in patients with non-union: preliminary results

L. Cevolani*, D.M. Donati, T. Frisoni, M. Di Liddo, B. Dozza, E. Lucarelli, S. Giannini

Istituto Ortopedico Rizzoli (Bologna, IT)

**Introduction** The non-union looks radiographically as a fracture callus not very evident or absent 6 months after osteosynthesis. The incidence varies from 5 to 10% of fractures of long bones. However, patients undergo a long period of immobilization and this fact causes the increase the social cost of the disease. The technique we suggest aims to reduce the period of immobilization and as a consequence the management costs of the disease.
**Materials and methods** Our technique includes the infiltration of the nonunion focus with platelet rich fibrin (PRF), bone marrow concentrated (BMC) and demineralized bone matrix (DBM). Outpatients and radiographic checks were carried out 3, 6 and 12 months after surgery, and then once a year.
**Results** From November 2008 to March 2010 we treated 9 patients (average age, 36 years; range 19–53). The sites affected are tibia (4), femur (4) and radius (1); two patients had an open fracture. The ostesynthesis was performed with intramedullary nails (3), external fixators (3) and plate (1). All the patients had pain and could bear partial load. We performed our treatment after an average period of 8 months (range 4–15) from the fracture. The average follow up was 8 months (range 3–18). After 3 months from surgery, seven patients bore full load, did not feel pain and X-rays showed an increase of osteogenesis which permitted the removal of the external fixator in 3 patients. Two cases failed; in one of them the patient underwent another infiltration and in the other one we replaced the fixation.

**Discussion** There are many therapeutic strategies in case of non-unions and they include the replacement or dynamization of fixation, the osteosynthesis with or without a splint versus bone, the external fixation and the use of adjuvant (electrical stimulation, magnetic fields, bone grafting, use of BMP's). These procedures require that the patient reduce physical daily activity. The technique we suggest promotes faster healing, helps the patient to be able to bear load soon and thus it contributes to the reduction of the management costs of the disease.

**Conclusions** The application of demineralized bone matrix, platelet rich fibrin and autologous bone marrow concentrate lets a reduction of healing times in patients with non-union. It also gives the possibility not to wait for long after ostesynthesis so as to be able to perform the surgery that is minimally invasive and can be performed through the 1 day Surgery Programme.

### *C23—TRAUMATOLOGY 7*

### Achilles tendon rupture: percutaneous repairing

N. Galvano*, G. D'Anna, M. D'Arienzo

Clinica Ortopedica e Traumatologica-Policlinico Universitario (Palermo, IT)

**Introduction** Achilles tendon ruptures are a common problem observed in a large segment of the population; the majority of cases are observed between 30 and 50 years, particularly in sports, and with a clear preference for the male sex. Elderly patients are commonly affected due to vascular, metabolic and hormonal disorders or because they use drugs that can induce a spontaneous rupture. Bilateral simultaneous at rupture is extremely rare.

**Materials and methods** Since January 2007 to December 2009 we treated 7 patients (5 m, 2f; mean age, 34 years) who were submitted to percutaneous repair technique. We repaired the tendon using a cannulated suture needles with MAXON zero absorbable type threads performed in regional anesthesia. After the surgery an immobilization with dynamic bendage is applied for 30 days. The follow-up was done at three, six and 12 months. We reached Optimal Functional outcomes in 95% of cases. Pain was evaluated with the visual analog scale (VAS) and a test to check the ROM of the ankle was made in 3 and 6 weeks after the operation.

**Results** The results of the technique were compared with the following parameters: return to work and daily activities, complications in the short and long term. Our outcomes, compared to data linked with open repair technique, confirm that we obtained a reduction of postoperative pain and a functional recovery with no aesthetic damages.

**Discussion** Since January 2007 to December 2009 we treated 7 patients with Achilles tendon rupture, performing a closed percutaneous tenorrhaphy with absorbable thread. Surgery was indicated to avoid a not correct healing of the tendon with an elongation and a loss of efficiency of the sural triceps.

**Conclusions** The subcutaneous Achilles tendon rupture is not a very common lesion but highly disabling. An early diagnosis is possible by ultrasound and the appropriate treatment is a closed tenorrhaphy. According to our experience, we can conclude that the percutaneous suture technique with absorbable thread is as effective as the open repair.

### The treatment of fractures in patients with a previous osteosynthesis

P. La Floresta*, S. Miceli, F. Fascione, A.A. Cavero, A. Gennarelli, M. Ialonardi

U.O.C. Ortopedia e Traumatologia, Ospedale Antonio Cardarelli (Campobasso, IT)

**Introduction** Fractures in patients who had a previous osteosynthesis is an emergent pathology and although they were infrequent until few years ago. They are now appearing with increasing frequency because of the increasing average age of patients, their higher level of activity and the better functional results of a new osteosynthesis.

**Materials and methods** From January 2000 to December 2010 in the U.O.C of Orthopaedics and Traumatology of our Hospital we performed 13649 surgeries, 5869 (43%) were trauma interventions. We had to deal with fractures on implants in 28 cases and in 57 cases with fractures on prothesis. All the fractures on synthetic implants were surgically treated, by expanding or modifying the intramedullary synthesis when possible (19 cases), or using a plate with screws implant in 9 cases.

**Results** Results of the II osteosynthesis surgery were excellent in 6 cases; good in 8 cases; tolerable in 9 cases; bad in 5 cases. Results of new osteosynthesis surgery were influenced not only by the quality of the implant and the obtained stability, but also by the age of patients. Five out of the 6 excellent results were obtained in patients younger than 60 years, the 5 bad results were all obtained in patients older than 80.

**Discussion** The appearance of "peri-sinthetic" fractures was attributed to the stress concentration on the extremes of the implant caused by the different structural rigidity between the normal bone and the bone-synthesis part. The difference in structural rigidity is higher in osteoporotic bone where the concentration of stress in the marginal junction of the bone-synthesis conglomerate, contribute to increase the risk of fracture in proximity of the implant apex. In order to obtain a good functional result, it is essential to resume the synthetic implant so to have a good stability and to allow an early articular mobilization.

**Conclusions** The stability of the implant and the bone quality are decisive in the choice between the extension of the intramedullar or marginal osteosynthesis, or its integration. When possible the substitution of intramedullary "short" implant with a bigger one is the best choice, for the simplicity of the surgery and stability of the implant and therefore the rapidity of functional recovery.

### Open pelvic injury: first and delayed treatment

C. Battiato*

ASUR 13 (Ascoli Piceno, IT)

**Introduction** Open pelvic injuries are a multidisciplinar emergency. There are very difficult choices that one must take in the first emergency fase but also challenging decisions to do in the second later fase. The outcome is directely determined from these right or wrong choices. We try to analyze the current protocols and to describe a correct treatment algorithm.

**Materials and methods** After a literature review we analyze the possible treatment options that may be taken in two different situations: one in spoke model hospital and the other in hub model hospital. In the first model there are some key points that everywhere and by everyone must be correctly performed so that only stable patients must be transferred in the second level hospital for ultraspecialized second treatment.
**Results** The outcome must be improved both in surviving level and in functional results. We try to determine what must be done and where in a sort of list of problems and relative solutions.
**Discussion** We are dealing with a very severe pathology and dangerous lesions. The results are directly connected to the right and fast treatment. We believe that every hub center hospital needs a trained trauma leader and a good trauma team. In the first level spoke hospital is essential to apply the protocols in order to achieve good results.
**Conclusions** Pelvic open injuries are lesions very difficult to treat and every orthopaedic surgeon even if working in a first level hospital must know the basic treatment of these lesions.

### The first clinical Italian experience with a new electromagnetic targeting device for intramedullary nails

G. Antonini*, F. Valli, C. Crippa, R. Giancola

Ospedale San Carlo Borromeo (Milan, IT)

**Introduction** Intramedullary nailing is the standard treatment for diaphyseal lower limb trauma. The distal locking is even today a difficult part of the procedure, resulting in radiation exposure, loss of surgical time and iatrogenic complication. The literature about the new devices is controversial.
**Materials and methods** We analyzed prospectively 25 consecutive patients affected by femoral or tibial fractures and treated between September 2010 and February 2011. All fractures were treated with TriGen nail (Smith&Nephew) and the distal locking procedures were guided by a new distal targeting device named Sureshot (Smith&Nephew). The surgeon had a continuous visual real-time feedback of drill position by an intramedullary electromagnetic probe inside the nail and a monitor. We evalutated: effectiveness of the system, duration of procedure, time to position the screw, irradiation exposure, complications.
**Results** All but one of the 56 screws were perfectly inserted. Mean preparation time of the device was 6 min, mean time for single screw targeting was 6 min, with 2–14 fluoroscopic images per screw and radiation exposure always less than 1 s. The only failure was due to a malposition of the probe in the nail.
**Discussion** In this prospective study we did not have a control group treated with the standard free-hand technique, and the number of cases is not huge. Therefore according to the results this device is effective, very easy to learn, with lower surgical time compared to the literature. These data can counterbalance the disadvantage of the device cost.
**Conclusions** Sureshot system can be considered a reliable, effective and reproducible device reducing surgical time and radiation exposure.

### Tibial pilon fractures treated with hybrid external fixation

N. Tartaglia*[1], G. Corina[2], A. Marsilio[2], T. Gismondi[1]

[1]U.O.C. Ortopedia, P.O. “San Paolo” (Bari, IT);
[2]U.O.C. Ortopedia, P.O. “Vito Fazzi” (Lecce, IT)

**Introduction** Complex tibial pilon fractures are closed or open comminuted fractures often associated to soft tissue injury. These fractures can be treated with hybrid external fixation obtaining good and stable educations using ligamentotaxis principles and tensioned olive wires. Where possible this technique can be associated to a minimal internal synthesis and to the fibula fixation. However it is a complex technique requiring a long learning curve and outpatient clinic dedicated to external fixation.
**Materials and methods** From September 2004, at the “San Paolo” Hospital in Bari and “Vito Fazzi” Hospital in Lecce 80 tibial pilon fractures have been treated and evaluated in a retrospective study.
**Results** Most of patients showed an almost complete restore of joint range of motion after external fixator removal. Late consolidations, non-union post-traumatic arthritis and post traumatic deformities showed a percentage similar to the one described in literature with internal fixation techniques.
**Discussion** Sometimes closed reduction and mini-invasive surgical approaches cannot restore an exact joint surface congruency however it allows to reach good functional results minimizing all complications due to surgical invasive approaches (infections, skin necrosis, and late consolidations) and respecting the fragile biology of this peculiar fracture site. Patients can already start functional rehabilitation immediately after surgery and they can weigh bear earlier than patients with other internal fixation techniques.
**Conclusions** We think that hybrid external fixation is a valid surgical option offering a stable reduction with a mini-invasive approach allowing an early joint function restore minimizing complications.

### *C24—TRAUMATOLOGY 8*

### Neuropsychiatric disorders in hip fracture

G. Iolascon*, F. Gimigliano, G. Di Pietro, S. Gatto, R. Gimigliano

Seconda Università degli Studi di Napoli (Naples, IT)

**Introduction** Hip fractures are associated with a high rate of mortality and permanent disabilities, especially in the elderly. Hip fracture has an estimated mortality of 5% in the acute phase and 15–25% within 1 year. Motor disability after hip fracture is permanent in 20% of cases and only 30–40% of patients regain a level of autonomy compatible with pre-injury activities of daily living. Elderly patients with fractures are commonly affected by co-morbidities requiring specific assessment before and after surgery. Neuropsychiatric disorders such as delirium and dementia increase mortality within 6 months after hip fracture, Depression can have a negative effect in terms of functional outcome and survival even greater distance of time. Delirium is a common post-operative complication in elderly patients treated for hip fractures. Among the various forms of delirium, the hyperactive, characterized by psychomotor agitation and behavioral disturbances, is the most common subtype. Early diagnosis and an adequate treatment of these conditions must be performed in order to achieve an optimal outcome. We propose a diagnostic and therapeutic algorithm for early detection and intervention of neuropsychiatric complications, after surgery for hip fracture, in orthopedic and rehabilitative settings.
**Materials and methods** *Preliminary assessment*: (1) cognitive impairment (Short portable mental Status Questionnaire); (2) depression (Geriatric Depression Scale 4 item); (3) delirium (Diagnostic Criteria for delirium DSM IV).
*Signs/symptoms of delirium, prodromic phase (6–48 h)*: (1) anxiety; (2) frequent request for assistance; (3) disorientation; (4) reduced attention; (5) psychomotor restlessness.

*Signs/symptoms of depression, prodromic phase (within the 2nd week)*: (1) anxiety; (2) pain; (3) lack of interest; (4) lack of initiative; (5) lack of motivation.
*Assessment of delirium in a rehabilitative or orthopaedic setting*: (1) CAM; (2) DRS-R-98; (3) OBS.
*Further assessment of depression in a rehabilitative or orthopaedic setting*: (1) HDRS; (2) GDS.
*Intervention program*: (1) monitoring and control of vital signs (Sat O2, PA, hb, TC); (2) monitoring and regulation of electrolyte balance and nutrition adequate pain treatment (paracetamol, opioids); (3) pharmacological treatment; (4) psychological support.

### Tibial plateau fractures: hybrid external fixation treatment

N. Tartaglia*[1], G. Corina[2], A. Marsilio[2], T. Gismondi[1]

[1]U.O.C. Ortopedia, P.O. “San Paolo” (Bari, IT);
[2]U.O.C. Ortopedia, P.O. “Vito Fazzi” (Lecce, IT)

**Introduction** Complex tibial plateau fractures can be mainly treated with two different surgical options: internal fixation (screws or plating and screws) and external fixation (monolateral, circular, hybrid). The choice of the hybrid external fixation in the closed tibial plateau fractures offers more stable reductions associated to a less invasive surgery, immediate joint mobilization already in the postoperative period, early weigh bearing, almost complete joint function restore till the external fixator removal, external fixator removed under a light narcosis avoiding other surgeries.
**Materials and methods** We present 40 closed tibial plateau fractures treated with hybrid Orthofix external fixator.
**Results** All fractures healed within 3 months with a good joint function restore and a good patient compliance followed every week in external fixation outpatient clinic. No joint stiffness has been observed. Two superficial infections were treated with oral antibiotics and wound dressing.
**Discussion** Tibial plateau fractures need surgical techniques allowing a joint reconstruction to grant a good joint function restore and minimizing invasivity to respect knee joint biology and to avoid post-traumatic stiffness.
**Conclusions** We believe hybrid external fixation is a good surgical option treating this kind of fractures.

### Minimally invasive plate osteosynthesis of the fibula fractures tipe A/B

C. Chemello*, C. Iacobellis, R. Aldegheri

Clinica Ortopedica (Padua, IT)

**Introduction** The treatment of the fracture with a MIPO technique has been validated for fractures of the proximal third of the humerus, femur, distal third of the tibia, while only one author reports his results with this technique in 20 patients (F. Hess) [1]. In this paper we report our experience with a group of 30 patients.
**Materials and methods** Thirty patients with fracture of the fibula Type 44 A/B/C were treated with using an angular stable screw-plate system using a minimaly invasive technique. Postoperatively a cast was placed for 2 weeks, therefore they were reviewed at 1 month and was granted the load. At 3 months patients were reviewed to confirm complete healing. Clinical and radiographic evaluation was performed with the card N-G-S (AO).
**Results** We got a positive result on 29 patients and only one failure. All fractures consolidated, we had only 1 case of superficial peroneal nerve stupor and 1 case of rigidity.
**Discussion** One of the most frequent problems in the treatment of fractures of the fibula, treated with traditional plate, is the risk of wound dehiscence. With this technique we obtained the complete disappearance of this problem. We did not had any problem of consolidation of the fractures. Furthermore, after 15 days could start an early mobilization and physical therapy, we had no cases of rigidity. Our only complication was a nerve injury which resolved spontaneously. However, that risk is easily eliminated by using plates not over 8 holes.
**Conclusions** For us, this treatment should be chosen for the trans-sindesmosiche unbloody manner reducible fractures. The surgical technique requires a proper learning curve but in our experience it is easy to learn.
**Reference**
1. Hess F, Sommer C (2011) Minimally invasive plate osteosynthesis of the distal fibula with the locking compression plate: first experience of 20 cases. J Orthop Trauma 25(2):110–115.

### Use of cement-tricalcium phosphate with combined techniques in the treatment of fractures of the tibial-plateau

M.P. Palomba*, M.I. Iacopinelli, M.M. Manca

ASL 12 Versilia (Lido di Camaiore-Lucca, IT)

**Introduction** Fractures of the tibial plateau represents a challenge for the orthopaedic surgeon because, there are many problems that these fractures should be evaluated and resolved. Among the early timing of the treatment that now seems to have a rational, common to all surgeons, to continue in surgical planning and in selecting the best combination. Patients with these fractures are not always young. The bone-stock of these patients and increasing the energy in these traumatic fractures leads to severe bone and cartilage damage in terms of comminution and displacement of the fragments. Always problems related to fracture have focused on the need for anatomical reduction of fragments and in restoring articular congruity with restoration of the axes and rotations. The autologous bone graft is certainly the best solution but in biological terms as stated in the not so young patients can cause problems such as donor site morbidity and surgical time dilation. Hence the decision to use a bone substitute that can bridge the gap and has high mechanical strength.
**Materials and methods** In complex fractures type C from 2009 we used calcium phosphate cement in slow synthesis and external fixation and in open surgery. In many cases, the reduction of the fragments was carried out by indirect methods and synthesis of the defect after filling or after filling and stabilizing. This was possible thanks to the version run “drillable” of the compound that can be punched out after a few minutes. The calcium phosphate cement has always shown an osseointegration without local or general reaction and the effective strength.
**Results** Patients were monitored, having been increasingly loaded by the 45th day onwards, no metaphyseal collapse was recorded. We observed good recovery of the ROM. In four cases there was a post-traumatic osteoarthritis with valgus deformity. In these cases, patients underwent surgery for total hip arthroplasty.

**Discussion** Surgical techniques, here described, combine direct and indirect reduction and synthesis based on the peculiarities of the fracture and the condition of soft tissues with external or internal fixation with plates.
**Conclusions** Calcium phosphate cement is effective in filling and maintaining the bone defect in tibial plateau fractures demonstrating a high mechanical strength proving particularly useful in minimally invasive techniques and combined.

### Leg fractures: indications and limits of intramedullary nailing with extended knee

A. Ciuffoletti*, C. Battiato, M. Rizzo

ASL 13, ASUR Marche (Ascoli Piceno, IT)

**Introduction** Use of intramedullary nailing in tibial fractures is a consolidated and routinary technique, nowadays applied even to open fractures, if surgery is performed within 6 h from the traumatic event. Authors propose an outcome evaluation when using a lateral parapatellar approach.
**Materials and methods** Sixteen patients were treated for a tibial fracture. Nails used belonged to different companies but all have similar technical characteristics. The surgical approach was a lateral and parapatellar, with the knee in extension. A lateral release was performed aiming to the proximal part of the tibial shaft, the medullary canal is 'opened' in front of the anterior tibial spine. The operation was achieved reaming and blocking the nail on a standard surgical bed, without traction.
**Results** Fractures healing was obtained in all cases in absence of stiffness, secondary to the surgical approach.
**Discussion** Advantages of this technique are many: better evaluation of rotational defects, chance of treating definetly open fractures initially stabilized with an external bridging fixation, chance of operate patients affected by stiff knees, faster patient positioning avoiding the traction bed, obtain easier fracture reduction when located in the proximal third of the tibia. Limits are: cartilage lesions, condilar or patellar, intrarticular postoperative bleeding, lateral patellar pain.
**Conclusions** The technique is simple when consolidated in surgeon experience. Learning curve is mined by complications.

### Treatment of complex tibial plateau fractures with Ilizarov external fixator

F. Santolini, D. Chiapale, M. Stella*

Ospedale San Martino (Genoa, IT)

**Introduction** Tibial plateau fractures represent a difficult problem to solve for trauma surgeon in relationship to conditions of soft tissues. Golden standard of treatment of tibial plateau fractures is anatomical reduction of particular surface and plate osteosynthesis. Often soft tissues conditions direct the choice of treatment.
**Materials and methods** We present our experience in treatment of complex tibial plateau fractures using Ilizarov circular frame in association with minimally invasive percutaneous or through a mini-open approach screw-fixation of the joint surface. We develop biomechanical principles of circular external fixation in tibial plateau fractures with regard to surgical indications and technical notes.
**Discussion** Circular external fixation in association with mini-open or minimally invasive screw-fixation of the articular surface has proved a viable treatment option for these complex fractures in which soft tissues conditions most of all influence the choice of treatment.

### Fractures of the patella today: use of absorbable cerclage

N. Galvano*, R. Adelfio, M. D'Arienzo

Clinica Ortopedica e Traumatologica, Policlinico Universitario-Palermo

**Introduction** Fractures of the patella constitute 1% of all skeletal injuries. The complications of a patellar fracture can be significantly relevant because the motion and the muscle tone are involved and sometimes may develop into osteoarthritis. They occur more frequently in patients between 40 and 50 years of age with a slight predominance in males. About the mechanism of injury, the fractures of the patella can be caused from direct trauma (a direct blow on the knee), indirect trauma (in the act of falling) o combination of direct and direct trauma.
**Materials and methods** The study conducted at the University Hospital of Palermo Orthopedic and Trauma Clinic included the observation of patients with fractures of the patella from 2007 to 2010. In 22 patients (15 men and 7 women), mean age 50 years, surgical treatment with fibrous cerclage open using MAXON and Fiberwire suture have been performed. Patellar fractures were classified according to both their morphology and the number of fragments.
**Results** Patients were checked at 15 days from the date of surgery and then monthly checks until the third month after surgery. Not being established to standardize the results, the authors have adopted a rating scale based on pain perceived by the patients (VAS-score) and functional recovery of the joint (Bostmann et al. scale) considered the most comprehensive. All patients had a recovery of motion of the knee in a short time without any complication in both the short and long postoperative period.
**Discussion** The adoption of this surgical technique offers the undoubted advantage of not having to expose patients to reoperation for hardware removal. The sutures that were used in the study in question, being absorbable not have the disadvantage of removing them.
**Conclusions** Based on the results of the study, the use of the fibrous cerclage is to be preferred to the metal one. Although the results overlap in terms of functional recovery, the fibrous cerclage prevents any pressure sores and does not require reoperation for hardware removal.

### Less invasive cerclage

C. Battiato*

ASUR 13 "Mazzoni" (Ascoli Piceno, IT)

**Introduction** The reduction of cortical bones mechanical resistance resulting from under pressure contact of any peripheral system device is well known to the orthopaedic surgeons and it is ascertained in daily trauma practice. Some influential authors have deepened the biological aspects and consequently have inferred the possible clinical complications. The orthopaedic industry, on the other hand, has promptly made available a new family of peripheral low contact synthesis devices (LC-dcp, PC-FIX, LISS, etc.) capable to restrict the cortical-plate contact to specific limited areas, thus eliminating the adverse effects of a reduced periosteal blood supply. It has always been our belief that the use of cerclage systems, useful both in trauma and in elective hip revision arthroplasty, can be an important aid in daily surgical practice. This is also shown by some authors who have remarked its application in the endomedullary nailing of fractures. We have therefore decided to introduce a new cerclage system, consisting of a wire and some spacers. This cerclage system, thanks to the spacers, reduces the perimetral extension of the applied pressure to the cortical bone and consequently prevents periosteal blood supply damage. We review the first cases treated with the above mentioned system and open the discussion on this topic.

**Materials and methods** 15 patients presenting with oblique fractures were treated with conventional synthesis devices (plates and nails) to which we added a cerclage system device. The average age of patients was 75 years (from 29 to 86). Patients underwent clinical and radiographic check-ups for 12 months.

**Results** A full recovery was achieved in all cases. Radiographic absorption around the cerclage or inhibition fracture union were not observed in any patient.

**Discussion** It has always been our belief that the use of cerclage systems, useful both in trauma and in elective hip revision arthroplasty, can be an important aid in daily surgical practice. This is also shown by some authors who have remarked its application in the endomedullary nailing of fractures. The mini-invasivity of percutaneous surgical techniques and the cerclage device reduced contact with the bone allow to reintroduce this auxiliary synthesis device in osteosynthesis procedures.

**Conclusions** This cerclage system, thanks to the spacers, reduces the perimetral extension of the applied pressure to the cortical bone and consequently prevent periosteal blood supply damage.

### *C25—INFECTIOUS DISEASES*

### Pyogenic and tuberculous spondylodiscitis: a comparative study on 103 patients

E. Pola*, L.A. Nasto, B. Rossi, G. Di Giacomo, D. Colangelo, M. Fantoni, C.A. Logroscino

Università Cattolica del Sacro Cuore, Policlinico “A. Gemelli” (Rome, IT)

**Introduction** Although rare, spondylodiscitis account for about 2–7% of all osteomyelitis. This study aims to evaluate potential predisposing factors and comorbidities of 103 patients affected from pyogenic and tuberculous spondylodiscitis.

**Materials and methods** We analyzed 103 patients with spondylodiscitis treated from 2008 to 2010 by a team of orthopaedic surgeons and infectivologists. In all cases diagnosis included a combination of clinical findings, imaging investigations and bacteriologic and histological tests. All patients were examined in terms of comorbidities, diagnostic approaches and isolation of etiological agents. We compared the predisposing factors and associated illness, clinical, radiologic and laboratory features, assessing a statistical significance for each variable.

**Results** Sixty-four male and 39 female were included in our series; the mean age was 64 years. The causative organism was isolated in 86% of cases and confirmed by culture from spinal biopsy specimens (42%), blood culture (34%) or wound buffer (10%). Staphylococcus was significantly the most frequent causative agent: *S. aureus* was found in 20% of patients, *S. epidermidis* in 15%, S. Hominis in 10%. Mycobacterium tuberculosis was isolated in 18% of cases. Fifty-six percent of patients had undergone previous invasive procedures, and spondylodiscitis was a complication of spine surgery in 21%. Interestingly, most of the post-operative infections of the spine were consequent to cardio-vascular or urologic approaches. Analysis of comorbidities showed that immunodeficiency due to chronic glucocorticoids therapy was associated in 18% of the cases, intravenous therapy or central venous access devices in 28%, intravenous drug abuse in 6% of cases. Among patients with debilitating chronic diseases, 12% were affected by HIV or viral hepatitis, diabetes mellitus in 15%. About 55% of patients referred a previous admission to hospital within 6 months from the time of infectious diagnosis. All the risk factors and comorbidities over-mentioned were statistically significant ($p < 0.001$) comparing pyogenic and tubercular spondylodiscitis.

**Discussion** Incidence of spondylodiscitis is rising in the last decades as a result of the increase of intravenous and immunosoppressive drug use, mini-invasive surgical procedures, and drift of immigrants from developing to industrialized countries. Also our data suggest that previous spinal or non-spinal surgical approaches, immunodeficiency, intravenous therapy or central venous access are the most frequent causes of pyogenic spondylodiscitis. Instead, the rising of tubercular spondylodiscitis seems to be directly related to the increasing of immigrants.

**Conclusions** Identification of statistically significant risk factors and predisposing conditions could contribute to early diagnosis and improvement of prognosis by means of specific therapeutic approaches.

### Two-stage uncemented hip prosthesis for the treatment of septic hip arthitis

C.L. Romanò*[1], D. Romanò[1], L. Monti[1], E. Meani[2]

[1]Istituto Ortopedico I.R.C.C.S. Galeazzi (Milan, IT);
[2]Istituto Ortopedico G. Pini (Milan, IT)

**Introduction** Septic arthritis of the hip is a rare but potentially devastating condition. Many risk factors have been described, including diabetes, rheumatoid arthritis, steroid therapy, etc. Girldestone described in 1943 the resection arthroplasty for the treatment of septic coxarthritis. Although this procedure is generally able to effectively control the infection, it is associated with a variable degree of residual pain, functional impairment and limb shortening. Very few studies have provided data regarding the possibility of alternative treatments on continuous series at long-term follow-up.

**Materials and methods** 19 patients (20 hips) underwent total hip implants in two stages, according to a same protocol, in the years 2000 to 2008. In all cases the first stage included the resection of the femoral head, a thorough debridement of infected tissue and the system of a temporary spacer (Spacer-G, Tecres SpA, Sommacampagna, Verona) through a lateral approach. In all cases, a cementless total hip prosthesis was implanted in the second stage.

**Results** The mean interval between the first and second operation was 22 ± 5.1 weeks. After a mean follow-up 56.6 months (range 24–104) after prosthetic implant, only one patient had a recurrence of infection. The average Harris hip score increased from 27.5 (range 15–56) to 61.8 (range 52–85) between the two stages and to 92.3 (range 68–96) at the latest follow-up.

**Discussion** Chen et al. [1] reported a reinfection rate of 14%, with complications in 36% of the cases of two-stage reimplantation after septic arthritis of the hip, without the use of a spacer, while only isolated case reports [2, 3] propose the use of an antibiotic-loaded cement spacer, and then of prosthesis. This work contains the most extensive and consistent case series in this field published so far.

**Conclusions** Two-stage reimplantation with the use of a preformed hip spacer and a cementless hip prosthesis is a viable option for the treatment of adult patients with septic arthritis of the hip. The proposed technique is easily standardized and can be replicated in other centers, thus allowing a reliable solution to this serious condition.

**References**

1. Chen CE, Wang JW, Juhn RJ (2008) Int Orthop 32:573–580.
2. Schoellner CS, Fuerderer Rompe JD et al (2003) IARC Orthop Trauma Surg 123:254–259.
3. Regis D, Sandri A, Rizzo A et al (2010) Int J Infect Dis 14(3): E259–E261.

### Periprosthetic infections in critical patients: a challenge for orthopaedics

C. Pempinello*[1], T. Ascione[2], M. Conte[2], A. Bova[1], R. Pempinello[2]

[1]Ospedale S. Gennaro (Naples, IT);
[2]Azienda Ospedaliera Monaldi-Cotugno-C.T.O. (Naples, IT)

**Introduction** The basis for treatment is a highly specific, problem-adapted therapy with a defined strategy based to an early and radical surgical eradication of the affected bone and soft tissue in order to preserve the stable weightbearing bones, maintain a good mechanical axis with correctly working muscles and joints, and avoid permanent disability. Therefore, the algorithm defines a rational surgical and antibiotic treatment strategy [1].

**Materials and methods** From July 2009 to January 2011, 15 patients with infection following hip and knee replacement were treated (12 women e 3 men). The median patients' age was 72 years (42–82 years). Hypertension, obesity and diabetes mellitus were the most common underling illnesses.

**Results** Debridement and placement of a temporary spacer was applied in 10 patients, whereas 3 patients were underwent a surgical debridement followed by a prolonged antibiotic regime. Furthermore, the last 2 patients affected by late infection were treated only with antibiotic therapy. Follow-up showed satisfactory results, except in 4 cases. In 1 patient with a severe polimicrobic infection related to *Pseudomonas Aeruginosa* ESBL and MRSA the infection relapsed 8 months later, so that the spacer was removed without any replacement. Other 2 patients have still the spacer considering their critical clinical conditions. The last patient affected by MRSA infection with a femoral pathological fracture not yet healed is still waiting replacement of the spacer with a prosthesis.

**Discussion** The treatment and prognosis of adult osteomyelitis and prosthetic joint infections correlate with the clinical stage of the disease. According to McPherson classification, we have treated 3 patients with infection type C where arthrodesis or leg amputation was reasonable [2]. However, our efforts were finalized to eradicate infections and to improve postoperative outcome sometime with delayed removal of the spacer.

**Conclusions** Whereas the management of native-joint infection and osteomyelitis is well established, the management of infection associated with prosthetic joints is less standardized, because of the variable clinical presentations and the lack of data from randomized, controlled trials. Despite the importance of different surgical options, antimicrobial therapy in the treatment of osteomyelitis is unquestioned [3].

**References**

1. Zimmerly W, Trampuz A, Ochsner PE (2004) N Engl J Med 351:1645–1654.
2. McPherson EJ, Woodson C, Holton M, Roidis N, Shufelt C, Patzakis M (2002) Clin. Orthop 403:8
3. Ascione T, Pagliano P, Pempinello C, Rotondo R, Mariconda M, R.Pempinello (2011) J of Infec (*In press*).

### Sonication technique for microbiological diagnosis of periprosthetic knee infections

A. Schiavone Panni[1], M. Vasso*[1], S. Cerciello[1], M. Lichtner[2], L. Scorzolini[2]

[1]Dipartimento di Scienze per la Salute (Campobasso, IT);
[2]Dipartimento di Malattie Infettive (Rome, IT)

**Introduction** Deep infection remains the second cause of implant failure after total knee arthroplasty. The increasing number of prosthetic joint replacements that are every year performed in all over the world has resulted in a concomitant rise in bacterial infections, and despite the use of systemic antibiotic prophylaxis, strict hygienic protocols, and special sterile enclosure with laminar flow, the infection rate in primary total knee replacement is between 1% and 3%, with a mortality rate of 2.5%. The etiologic diagnosis of infection at the site of a total knee arthroplasty is detrimental but it can be complex. Aspiration of synovial fluid often results negative, as micro organisms infecting a total knee generally proliferate in a bacterial biofilm.

**Materials and methods** The sonication method for microbiological diagnosis in implant-associated orthopaedic infections has not yet introduced in routine clinical practice. Sonication might improve microbiological diagnosis analyzing fluid obtained from removed prosthetic components.

**Results** In a study on 41 periprosthetic knee infections, sensitivity of standard culture methods and sonicated fluid cultures was 42 and 76%, respectively. Coagulase-negative Staphylococcus was the most frequent microorganism detected by sonicated cultures (81%), while standard cultures showed growth of this microorganism only in 41% of the cases. Standard cultures failed to detect any bacterial growth in 69% of the cases.

**Discussion** Sonication might improve sensitivity of etiologic diagnosis in infected total knee arthroplasties.

**Conclusions** Our study showed sonication to be useful in Coagulase-negative Staphylococcus infections in which biofilm formation plays a central role.

### "Granulomatous inflammation" in HIV positive patients: not just tuberculosis

G. Rorato*[1], E. Zamparini[1], B. Del Pin[2], F. Bragantini[2], A. Pagotto[2], M. Merelli[2], G. Villa[2], F. Brillo[2], B. Cappelletto[3], G. Cattani[2], P. Viale[1]

[1]UO Malattie Infettive, Dip. Attività Integrate Malattie Apparato Digerente e Medicina Interna, Università degli Studi Bologna (Bologna, IT);
[2]Clinica di Malattie Infettive, Dip. Scienze Mediche e Morfologiche, Università degli Studi di Udine (Udine, IT);
[3]Chirurgia Vertebro-Midollare e Unità Spinale, Azienda Ospedaliero Universitaria Santa Maria della Misericordia (Udine, IT)

**Introduction** Infections with nontuberculous mycobacteria (NTM) usually affect immunocompromised patients. *M. xenopi*, mild pathogen, rarely causes spondylodiscitis, mostly post local surgery

(exogenous). Seven are the known cases of not postoperative spondylodiscitis (endogenous) from *M. xenopi*. The etiologic diagnosis, difficult due to the peculiar conditions of growth in culture, should be pursued as *the M. xenopi* has a profile of drug treatment, that results in significant difficulties with the risk of complications for the patient.

**Materials and methods** A forty-year-old patient, known HIV + -since 1993 (stage C3), in HAART, in 2001 had been diagnosed (imaging and blood cultures) with *S. epidermidis* related L1-L2 spondylodiscitis. In 2006, for recurrence of symptoms, M. Pott's L1-L2 was diagnosed histologically, on bone biopsy (granulomatous inflammation), without microbiological confirmation. The empirical anti-tuberculosis therapy, poorly tolerated, needed more suspensions and regimens, with apparent improvement. No surgical indications for spinal stabilization were given. In August 2009, a lumbosacral MRI control (patient asymptomatic, CD4 880/μL, suppressed viremia, ESR 98 mm/h, CRP 5.20 mg/L), detected recurrent disease, with a large multilocular abscess to the muscle-ileum right psoas, confirmed on the PET F18-FDG (SUV max 30). In the purulent material drained from the muscle was isolated *M. xenopi*. Then, in October 2009, the patient assumed moxifloxacin, ethambutol, and clarithromycin. CAMP bust C-35 was adopted but, for the absence of neurological symptoms, it is waived debridement and stabilization.

**Results** Lumbosacral MRI at 1 month of treatment was unchanged, while the $F^{18}$-FDG PET significantly improved (SUV max 10) (patient asymptomatic, CD4 942/μL, suppressed viremia, normal ESR and CRP). At the later radiological control (3, 6 and 12 months of therapy), the picture progressively improved. To date, the patient is on therapy, scheduled for >18 months, according to tolerability and radiological evolution.

**Discussion** It is the 3rd report of spondylodiscitis endogenous *M. xenopi* and the 1st of ileus psoas muscle abscess in patient HIV + never undergone spinal surgery. Nonspecific clinical and histological appearance similar to that of the more common *M. tuberculosis*, make the risk of mis-diagnosis high. In HIV + patient with spondylodiscitis histologically attributable to mycobacteriosis, should be pursued a microbiological date to exclude NTM, even rare, like the *M. xenopi*, in order to start adequate treatment, as the prognosis is unfavorable if not appropriately treated. Medical therapy takes advantage of the surgical approach in case of ileus psoas abscess, and spinal surgery is limited mostly to cases of neurological complications.

### *C26—HAND AND WRIST 1*

### Percutaneous fixation for scaphoid fractures with HCS screw

C. Iacobellis, C. Chemello*, R. Aldegheri

Clinica Ortopedica (Padua, IT)

**Introduction** Fractures of the scaphoid type B1 and B2 according to Herbert, are generally treated with casts or percutaneous screws. Some authors prefer surgical treatment to reduce patients' healing times and the possibility of returning to work earlier [1]; other still prefer casts, which are believed not to be prone to complications. Yet others select patients for casts according to the type of the work they do [2]. In this study we report our experience in percutaneous fixation for scaphoid fractures with HCS screw in 44 patients.

**Materials and methods** We treated 44 fractures of the carpal scaphoid (34 men, 10 women; mean age 32 years, range 14–60). Fractures were assessed by X-rays, separated into B1 (11 cases) and B2 (33 cases) according to Herbert. Patients were treated with a percutaneous HCS screw within 48-h of injury. The wrist was immobilized with a short thumb-spica cast for 2 weeks. X-ray check-ups were made at 6 and 8 weeks after surgery. We used Quick-DASH Score and the Mayo Wrist Score for the clinical evaluation.

**Results** We had a consolidation in 43 cases within 5 month. Only in one patient did pseudoarthrosis developed, due to insufficient compression. The mean Quick-DASH score was 2.95, while the mean Mayo Wrist score was 94.16 In 3 patients, it was necessary to remove the screw 9 months after surgery, because of a too long screw. There were no cases of infection, necrosis or radial dysesthesia.

**Discussion** There are a lot of different approaches to this type of fractures, we can assert that this surgical technique can reduce if not eliminate, the risk of nonunion of the scaphoid. The only case of nonunion and 3 cases of pain are all removable after a proper learning curve of the surgical technique [2].

**Conclusions** To us, this turns out to be the treatment of choice in fractures of the scaphoid, in patients requiring rapid and safe recovery with the exception of compound fractures of the tubercle or adolescents. In patient with poor functional requirements can opt for conservative treatment.

**References**

1. Modi CS, Mancoo T, Powers D, Ho K Boer R, Turner SM (2009) Operative versus nonoperative treatment of acute undisplaced and minimally displaced scaphoid waist fractures: a systematic review. Injury 40:268–273.
2. McQueen MM, Gelbke MK, Wakefield A, Will EM, Gaebler C (2008) Percutaneous screw fixation versus conservative treatment for fractures of the waist of the scaphoid. J Bone Joint Surg Br 90:66–71.

### Fracture of distal ulna: an additional instability factor in distal radius fractures

J.C. Messina*, F. Torretta, V. Pedrini

Istituto Ortopedico Gaetano Pini (Milan, IT)

**Introduction** Fracture of the ulnar head can be associated to distal radius fractures in severe trauma of the wrist. Frickman in 1967 was the first to identify these fractures as a factor of additional instability. Internal fixation of the distal radius only is not sufficient to obtain a stable fixation and an early mobilisation of the wrist. Subsequentely immobilisation has to be prolonged and stiffness of the wrist can occur with delay in rehabilitation program. Different modern therapeutic options exist for stabilisation of distal ulna as internal fixation with plate and screws, pinning, external fixation. The aim of this study is to evaluate the results of the different treatment options in distal radius and ulnar fractures

**Materials and methods** From 2006 to 2009, 23 patients affected by distal radius and ulnar fractures were treated. Internal fixation with plate and screws of distal radius and ulna was performed in 15 patients. In 8 cases internal fixation of distal radius was performed with pinning or external fixation of distal ulna. Internal fixation of distal radius was performed by volar approach using AO LCP 2.4 plates or De Puy plates and 2.00 or 2.7 mm AO plating for distal ulna or pinning and external fixator (Joshi) by posterolateral approach. Mean age of patients was 65 years (range 26–78). There were 20 females and 3 males. All patients were reviewed at 30, 60, 90 days post-operatively and then at a mean follow-up of 21 months (range 4–34 months). Plaster slab was removed at 30 days in the first group, at 50–60 days in the second group. Then physiotherapy was started. Mayo wrist score, DASH score and PRWE were evaluated at follow-up.

**Results** All patients of the first group (internal fixation of distal radius and ulna) obtained an excellent result, with quick recovery of full ROM and absence of pain. In 2 cases a slight reduction of grip (10%) was recorded, Mean DASH was 5.3 (0–10.8) ad mean PRWE was 10 (0–38). Excellent and good results were obtained in the second group (internal fixation of distal radius and pinning or external fixation of distal ulna) but rehabilitation was not possible untill 50–60 days postoperatively so full function was recovered several months later (mean 4 months). All patients returned to previous work or occupations.
**Conclusions** Association of distal radius and distal ulnar fracture is not very common but it is an additional instability factor in distal radius fracture. If the ulnar fracture is left alone the immobilisation time has to be prolonged up to 60 days with a delay in rehabilisation program and stiffness of the wrist. Modern treatment options allow stabilisation of the fractures. Early mobilisation is possible with double plating, while the use of pinning or external fixation delays the rehabilitation up to 50-–60 days. The distal ulnar fragment is not always easy to fix as it is often small of multifragmented. In those cases plating is not possible and pinning with external fixation is advised. Our results show that internal fixation of distal radius and ulna is the best treatment option when possible as it allows an optimal stabilisation of the fragments and early mobilisation with excellent results.

### Wrist fractures in young patients with associated capsule-ligament injuries: arthroscopic evaluation today

N. Galvano*, S. Moscadini, A. D'Arienzo

Clinica Ortopedica e Traumatologica, Università di Palermo (Palermo, IT)

**Introduction** Wrist fractures in young patients are on the rise due to the increase of direct traumas to the wrist. Compared with elderly patients, these kind of traumas occur for high-energy traumas so it's conceivable an association with capsule-ligament injuries. In 90% of cases, these lesions are undetected with a serious situation of arthritis and instability in the medium and long term.
**Materials and methods** On this basis, we observed since January 1st 2008 to December 30th 2010, 42 patients aged between 18 and 50 years with one or more fragments wrist fractures, determined by high-energy traumas. In all patients, a pre-reduction MRI and a diagnostic arthroscopy were performed to discover an associated capsule-ligament injury: we found in 29 patients a scapholunate dissociation (between I and III stadium) treated in the same operative session. All patients were then monitored at 1, 3, 6 and 12 months later, using VAS scale, DASH scale and Mayo Clinic.
**Results** In 90% of cases, we reached an excellent functional recovery, no pain, and a job activity resuming in 6 months after the treatment. Only 10% of cases had a moderate result caused by an incomplete functional recovery due to a not appropriate physiotherapy.
**Discussion** The MRI evaluation and the diagnostic arthroscopy in young people with wrist fractures is the gold standard to have a correct diagnosis useful to highlight a capsule-ligament injuries that otherwise would go undetected missed. Please note that the scapholunate dissociation is the cornerstone of a carpal instability with evolution to a SLAC (Scaphoid Lunate Advanced Collapse).
**Conclusions** According to our experience about young patients with wrist fractures, It is always necessary to evaluate the entire wrist capsular ligaments apparatus by an MRI; but especially, the pre-diagnostic arthroscopy should be made in all fractures where an implication of the scapholunate ligament is suspected, and in these cases the arthroscopic technique is the gold standard according to our experience.

### Treatment of pseudoarthrosis of the carpal scaphoid with platelet gel

N. Galvano*, A. Conti, S. Mannino, A. D'Arienzo

Clinica Ortopedica e Traumatologica, Policlinico Universitario (Palermo, IT)

**Introduction** Fractures and pseudoartrosis of carpal scaphoid are relatively common for an increasing number of car accident injuries as well as for a late diagnosis. This delay in diagnosis may result in a nonunion and evolve in carpal instability.
**Materials and methods** 13 cases of pseudoarthtrosis in carpal scaphoid have been treated in our clinic from January 2008 to December 2010. All of them were classified following the classification of Herbert, which, according to us, is the most complete and exhaustive. The treatment consisted in a fixation with Herbert screw and use of platelet gel, both in the scaphoid body by drilling the bone to create cavities as well as in the joint cavity. In both of cases in post-operative period antibiotic prophylaxis and anti-thrombotic one was performed.
**Results** The follow-up was performed by a minimum of 6 months to a maximum of 12 months and both the objective results than the subjective one were evaluated, using both the Mayo clinic as well as the DASH scale. In three cases RMI, as well as X-ray examination, was performed to evaluate the vascular supply. The clinical and instrumental results were very good with a good functional recovery and pain relief and without any secondary necrosis.
**Discussion** Functional recovery is always conditioned by the physiotherapy. In three cases, we also added the use of magnetic therapy. The subjective evaluation is helpful but not stable over time.Complications seems to be connected to a local infection, always resolved. There was either no vascular impairment or secondary carpal collapse.
**Conclusions** The indication to the synthesis associated with use of platelet gel results in a complete bone healing without any secondary necrosis reducing carpal collapse.

### Distal radius fractures treatment with plates and multidirectional angular stable screws

D. Bazzoni*, A. Mazzucco, S. Rossi, M.W. Rossi, M. Merlo, A. Grisoli

Ospedale di Circolo di Busto Arsizio (Busto Arsizio, Varese, IT)

**Introduction** In the last 5 years we have seen many changes in the surgical strategies for the treatment of distal radius fractures. Many patients, young and elderly, refuse cast and ask for early recovery of function of wrist and hand. Many companies have produced anatomically contoured plates with locking screws, very helpful for fixing distal radius fractures, even if in osteoporotic bone. Volar plating is the preferred choice of almost all Authors, due to the advantages of these new materials.

**Materials and methods** In the last 3 years we used mostly plates with screws having multidirectional angular stability. Screws can pivot freely by ±15° in all directions for optimal positioning, having always secured an angular stable locking in the plate. The surgeon can choose the better plate and the better directions of the screws, to fix even troublesome fragments; the system is precisely fitting to the anatomy of patients and to the pattern of the fracture, so volar plating is even more preferable.
**Results** In the last 5 years we treated about 250 distal radius fractures. Since 2007, 130 of them were fixed with plates and multidirectional angular stable screws, 95% with volar plating. We reviewed the data of these 130 cases. All patients started immediately active motion 2 days after surgery. Mean time of recovery of normal domestic use of the hand was 20 days; mean wrist ROM after only 2 months was extension 67°, flexion 62°, supination 85°, pronation 87°. We had no infections and no plate intolerance.
**Discussion** In the displaced stable or unstable distal radius fractures, the treatment of choice, for many Authors, is fixation with volar plate and locking screws. Many articles have evidences of better long-term outcomes for the patients who had surgical treatment versus patients who had conservative treatment.
**Conclusions** Multidirectional and angular stable screws give, in the treatment of distal radius fractures, a further advantage in order to obtain good surgical results and early recovery of wrist function.
**Suggested reading**

1. Mehling I, Meier M, Schloer U, Krimmer H (2007) Multidirectional palmar fixed-angle piace fixation for unstabie distai radius fracture. Handchir Mikrochir Plast Chir 39:29–33.

### Fixed-angle plates for the treatment of wrist fractures in patients older than 65 years

L. Tarallo*[1], R. Mugnai[1], R. Adani[2], F. Catani[1]

[1]Clinica Ortopedica e Traumatologica, Università degli Studi di Modena e Reggio Emilia (Modena, IT);
[2]U.O.C. di Chirurgia della Mano e Microchirurgia, Policlinico G.B. Rossi (Verona, IT)

**Introduction** The purpose of this study was to evaluate the efficiency of fixed-angle plates of the latest generation in the treatment of wrist fractures in elderly patients older than 65 years. The study included patients with fractures of the wrist type B and C of the AO classification.
**Materials and methods** From January 2000 to February 2010, 42 wrist fractures were treated, 24 of *Group B* and 18 of *Group C*. The average age of patients ranged from 65 to 87 years. All patients were followed for 13 months on average after surgery. In 10 patients, preoperative CT scan was performed with three-dimensional reconstructions.
**Results** The average joint mobility was 62° in extension and 60° in flexion. The grip strength was 85% of contralateral. The final radiographic parameters were, on average, 9° of volar inclination and 22° of ulnar inclination with 2.4 mm of ulnar negative variance. All patients were evaluated according to the Mayo Modified Wrist Score. They obtained a very good/good result in 76% of the cases, and a medium/low result in the remaining 24%.
**Conclusions** All fractures were treated with LCP systems of new generation and DVR plate of Hand Innovation with the use of Peg smooth instead of conventional screws, which have shown sufficient reliability and stability even in the presence of complex fractures such as type C in osteoporotic patients with poor bone quality.

### Intra-articular distal radius fractures: a clinical and radiographic comparison of treatment with volar angular stability plate and percutaneous pinning with K-wires

G. Campochiaro*[1], C. Tsatsis[1], G. Gazzotti[1], M. Rebuzzi[1], V. Tronci[1], C. Rovesta[1], F. Catani[1]

(Modena, IT)

**Introduction** Two surgical techniques for treatment of intra-articular distal radius fractures are compared: ORIF with plate versus percutaneous pinning with K-wires. Purpose of the study is to analize and compare vantages and disadvantages of these two techniques in long term.
**Materials and methods** We reviewed 78 intra-articular distal radial fractures type B and C (according to AO classification). These fractures were treated surgically from 2005 to 2009. For this study they were divided into two omogeneous groups according to patient age, gender, fracture type and follow-up. The first group was treated with ORIF using Aptus-Mikai angular stability volar plate, the second one with closed reduction, K-wires percutaneous pinning and ante-brachial plaster casting. Functional outcomes were assessed with MAYO score, DASH questionnaire; wrist range of movement and handgrip strength compared to controlateral side. Radius Shortening and dorsal radial tilt, obtained with these two surgical treatments, were calculated in the post-operative and long-term X-ray.
**Results** The mean follow-up was 29.5 months. ORIF group showed better mean DASH score (9 vs. 13) and MAYO score (80 vs. 77) compare to K-wires group. ORIF group showed also better range of movement and handgrip strength. Better clinical results were achieved in type C fractures for ORIF group especially in younger patients (<65 years). Minor differences were observed in type B fractures. No infections were noticed. Four cases of plate intolerance with consequent removal were noticed. Two cases of surgically-treated medial nerve compression in ORIF group and one case in K-wire group. Referring to antero-posterior and lateral projections parameters, long term radiographic values show data clearly positive for ORIF group.
**Discussion** Numerous options are available for treatment of intra-articular distal radius fractures. Although several studies on these two techniques have been performed, no scientific evidence proves the superiority of any surgical treatment. Therefore we want to contribute to this discussion sharing our clinical experience.
**Conclusions** Intra-articular fractures, especially C-type, need to be treated with plate in young patients or in elderly patients with good functional demand. Elderly patients (>75 years) with low functional demand can achieve satisfactory results also with percutaneous pinning, especially in B-type fractures.

### *C27—HAND AND WRIST 2*

### Nail injuries: treatment with platelet gel and nail reconstruction

N. Galvano*, G. Valenti, S. Moscadini, A. D'Arienzo

Clinica Ortopedica e Traumatologica, Policlinico Universitario (Palermo, IT)

**Introduction** The treatment of nail lesions of the fingers is important to preserve of the anatomical structures useful to the epicritic sensitivity. This kind of injuries is classified according to topographical criteria or through the PNB classification, considering the whole apical apparatus (nail, bone, soft tissues). This is necessary to reach an adequate evaluation and treatment.

**Materials and methods** From January 2007 to December 2010, we treated 35 nail lesions, generally of the right hand. II and III finger are mainly affected. In 84% of cases, patients were male manual workers. We removed the nail in all 35 cases, with suturing of the nail bed after bone reduction, where needed, grafting of platelet gel to the nail base and nail reconstruction, held for 45 days. Spontaneous physiotherapy was started after 15th day.

**Results** The evaluation was done at 3, 6 and 12 months, considering functional recovery, pain and sensitivity. All cases that we examined reported no pain, already in 3 months after trauma. The hyposensitivity was significantly reduced in 6 months, to disappear at all in 1 year. A functional limitation of the distal phalanx kept only in 6% of cases.

**Discussion** Main goals of the treatment are the complete functional and aesthetic restoring of the distal phalanx. With the platelet gel and the nail reconstruction, we also observed a total neurological recovery with a resumption of the epicritic sensitivity, always damages by the trauma.

**Conclusions** Fingertips are injuries with a difficult solution linked to an incomplete recovery of function, aesthetics and sensitivities. By combining nail reconstruction and platelet gel, the risk of functional failures is significantly reduced.

### A preliminary experience in the use of APSI prosthesis to treat stylo-scaphoid arthritis and scaphoid pseudoarthritis

M. Rosati*[1], A. Poggetti[1], G. Ciapini[1], L. Dolfi[1], C. Casini[1], L. Bonini[1]

1[a] Clinica Ortopedica Universitaria (Pisa, IT)

**Introduction** The substitution of the partial scaphoid replacement with pyrocarbon implants APSI is designed to treat stylo-scaphoid arthritis, and to prevent further deterioration and carpal collapse by restoring the first carpal row to its original height after scaphoid pseudoarthritis (SNAC wrist) or following scapho-lunate dissociationis (SLAC wrist).

**Materials and methods** From 2008 to 2010 in the Ist Orthopaedic Department of Pisa 10 patients (4 SNAC, 3 nonunions, 2 SLAC, and 1 scaphoid bone cyst) were treated. The patients underwent clinical evaluation by Krimmer tests, X-rays of the wrist (to check the height of the carpus) and MRI in two cases. The APSI prostheses were applied with dorsal approach and capsular joint denervation. Patients were evaluated with a follow-up of 8 months with clinical evaluation, JAMAR dynamometer test and radiographic imagine.

**Results** No implant dislocation was detected by radiological investigation. We obtained 6 excellent results (average of 86 points), 3 good (average of 75 points) and 1 moderate (65 points) after the evaluation with the Krimmer test. The pain was gone in 8 cases and it decreased in 2 cases particularly in the wrist radialization. ROM has improved in all cases. To report an increase in grip strength evaluated by JAMAR dynamometer, the X-ray investigation showed the restoration of carpal height in 7 cases, while in 3 cases, this index remained unchanged.

**Discussion** The partial scaphoid replacement with pyrocarbon implants, APSI, allows to avoid further deterioration and carpal collapse preventing the degenerative changes to SNAC or SLAC wrist. These pirocarbon implants require little sacrifice of bone and have proven to be very well tolerated from a biological point of view.

**Conclusions** Despite the few patients treated, our experience with the APSI implants is hopeful. This prosthesis is a good solution to eliminate pain and to allow a relatively rapid recovery of ROM and grip strength.

### Fractures of the neck of the fifth metacarpal bone. Mid-term results in 35 cases treated by percutaneous transverse pinning

V. Potenza*, R. Caterini, S. Bisicchia, E. Ippolito

Università di Roma Tor Vergata (Rome, IT)

**Introduction** Fractures of the neck of the fifth metacarpal (boxer's fractures) can cause impairment of hand function when they heal in malrotation and/or in volar angulation of the metacarpal head. Conservative treatment is indicated for undisplaced or minimally displaced fractures, while in severely displaced fractures surgical treatment is indicated. Percutaneous transverse pinning represents an excellent surgical option to treat "boxer's fractures", especially when severe soft tissue swelling of the hand is associated, minimizing the surgical trauma and postoperative complications.

**Materials and methods** We report the mid-term results of 35 closed displaced boxer's fractures treated with percutaneous transverse pinning to the intact fourth metacarpal in all that cases in which malrotation of the fifth finger and volar angulation of the metacarpal head greater than 30° were associated with a severe swelling of the hand.

**Results** At an average of 25 months after surgery, no patient reported residual pain. All patients showed a good functional result. No patient had any clinically detectable rotational deformity of the fifth finger with a deficit of grip strength. All patients considered their result as good or excellent. At the final x-ray examination a residual palmar angulation of the head of the fifth metacarpal was found in 3 patients, with a mean of 7°.

**Discussion** Some authors obtained good results with retrograde intramedullary pinning for boxer's fracture. In our opinion retrograde fixation has the potential disadvantages of MCP joint stiffness and extensor lag, moreover it is technically more demanding than transverse pinning and the surgeon has a more definite learning curve.

**Conclusions** We recommend percutaneous transverse pinning in all boxer's fractures in which operative treatment is indicated, especially in patients with severe soft tissue swelling. The surgical procedure is easy to perform, surgical trauma is minimised and the outcome after surgery is generally good or excellent.

### Our experience with glomus tumors

G. Scaravilli*[1], I. Scaravilli[2], P. Bianchi[1]

[1]II Clinica Ortopedica S.U.N. (Naples, IT);
[2]Naples, IT

**Introduction** Glomus tumors are rare mesenchymal neoplasia. They represent about 2% of all cancers of the soft tissues and 1% to 5% of all tumors of the hand. Benign tumors arise from a neuromyoarterial mechanoreceptors, the glomus body. It is an arteriovenous thermoregulatory shunt of the blood flow in the dermis. Even if they may interest different anatomical regions, they have an elective location in the hand.

**Materials and methods** We report some cases of glomus tumor came under observation. A careful case history was made in order to highlight the presence of the symptomatological triad of Carroll: paroxysmal pain, hypersensitivity to cold, pinpoint pain evoked by simple touch (revealed by "Love's Pin Test"). Patients were all treated by surgical excision.
**Results** Surgical excision results in complete healing and disappearance of pain symptoms. In the cases treated, there were not recurrences during a follow up of 5 years. However, it is always necessary to inform the patient about the possibility of this event.
**Discussion** Several studies showed that glomus tumor recurrence after surgical excision in a range usually between 5 and 15%, with a maximum of 50%. The early recurrence is mainly due to incomplete removal of the tumor or the presence of an unrecognized multiple glomus tumor. The late recurrence is usually characterized by the development of a new glomus tumor near the site of the primary lesion removed. In other cases, the pain symptoms may reoccur due to the appearance of a cicatricial neuroma. Clinical history has shown that patients often come belatedly to the observation, after several diagnostic attempts that did not allow immediate recognition of the lesion.
**Conclusions** An early diagnosis of glomus tumor is not always easy. In fact, in some cases observed, the initial examination did not show significant signs of neoplasia. Therefore, a better knowledge of this condition is needed to arrive at rapid solution, through a proper diagnosis, in order to avoid wide excisions.

### Thumb arthritis at stage 2: complications in the treatment by pyrocarbon spacer (Pyrodisc)

S. Odella*, R. Sartore, F. Torretta

Istituto Ortopedico G. Pini, U.O Chirurgia della Mano (Milan, IT)

**Introduction** To date, the treatment of thumb arthritis has not identified a gold standard, just as there is not necessarily a correlation between the radiographic stage of the disease and pain, sometimes a good radiographic outcome of surgical treatment performed does not correspond to patient satisfaction.
**Materials and methods** In our Unit of hand surgery from January 2007 to January 2009, 24 patients affected by thumb arthritis at stage 2 by Eaton, in the absence of subluxation of the first metacarpal, were consecutively operated n. They were treated by osteotomy of the trapezium and resection of the cartilage surface of the first metacarpal bone and positioning the pyrocarbon spacer (Pyrodisc type), stabilized by the flexor radialis carpi inserted in a tunnel into the bone of the trapezium and first metacarpal previously prepared. Median age is 64 years (min. 51, max. 70), in 22 cases the patients were women, 2 cases were men. Patients were monitored until 2 years after surgical treatment with DASH evaluation board, and the board of pain VAS.
**Results** To date we have removed 4 spacers because of persistent pain in spite of the spacer seems to be well housed at the radiographs in all cases, surgical treatment involved removing the spacer and performing arthroplasty. In one of the four cases the removal was very messy for the formation of exuberant bone tissue which completely covered the spacer. A patient undergoing surgery 6 months ago developed a significant stiffness, with a functional limitation in adduction and extension of the thumb. This patient is currently treated by a conservative treatment.
**Discussion** The treatment of thumb arthritis by pyrocarbon spacer allows a remission of painful symptoms after 6 months of surgery due to the resentment of the donor site (flexor carpi radialis), a longer time than just arthroplasty. The suture of the capsule with the thumb in adduction or use of the abductor pollicis longus tendon instead of the flexor carpi radialis tendon has reduced the postoperative pain intensity and duration.
**Conclusions** The treatment of Pyrodisc type rizoartrosi by spacer allows a good functional recovery and preservation of the length of the first ray. Recovery times, however, are longer than other surgical techniques. This procedure also requires absolute precision in the choice of the size and positioning.

### *C28—PAEDIATRIC ORTHOPAEDICS*

### Legg-Calvé-Perthes disease: a new minimally invasive surgical technique

A. Cavaciocchi*, G. Chiarugi

Azienda Usl 4 di Prato (Prato, IT)

**Introduction** Legg–Calvé–Perthes disease (LCPD) is a degenerative disease of the hip joint that is characterized by idiopathic avascular osteonecrosis of the femoral head. Perthes is rare, occurring in approximately 5 out of 100,000 children per year. It is most commonly seen in persons aged 3–12 years, with a median of 6 years of age.
**Materials and methods** Up to now there is no valid resolutive treatment of the most severe form (according to Herring C) of LCPD. Treatment has traditionally centered on removing pressure from the joint to increase blood flow until the disease has run its course. Options include traction (also using an external fixator), braces (Atlanta Scottish Rite), and surgical intervention: pelvic osteotomy or femoral varus osteotomy. Pressure is minimized on the hip also through use of Botulinum Toxin treatment of hip adductor and iliopsoas ipsilateral to the disease.
**Results** We daily care for children with Cerebral Palsy (CP). Iliopsoas and adductor tenotomy is the most common surgery used to prevent a hip dislocation. In May 2007 we undertook this kind of surgery in a 6-year-old tethraplegic child (CP). This child had a LCDP of his right hip (classified as Herring C type). Follow-up evaluations were yearly. What it came out was a total regression of the LCDP and bone remodelling of the necrotic femoral head.
**Discussion** Based on this case we propose a new mini invasive surgery for LCPD. First step: adductor tenotomy, then release of the iliopsoas tendon from the lesser trochanter. Second step is formed of using a Orthofix Eight-Plate for the guided growth of femoral proximal epiphysis. This board achieves a temporary epiphysiodesis of the lateral side of the apophysis of the greater trochanter ipsilateral to the LCPD. As it is well known that it causes a progressive varus deviation of femoral head, changing the load point of the head.
**Conclusions** The rationale of this technique is to remove the pressure on the femoral head affected by LCPD with a mini invasive surgery. A validation study is though needed to be performed.

### Correction of ankle's angular deformity during growing phase: temporary emi-epiphysiodesis using Eight-plate

C. Vercio*[1], G.A. Vercio[1], C. Ribaudo[1], I. Guttadauro[1], L. Promenzio[2], M. Bisignani[2]

[1]Palermo, IT;
[2]Rome, IT

**Introduction** In the knee during growth phase, detection of angular deformities in varus or valgus, frequently force orthopaedic surgeons to intervene surgically. New techniques of assisted growth of knee were eventually joined to osteotomies conducted on mature bone, performing a temporary emiepiphysiodesis applying an Eight-plate on more developed articular margin to allow the controlateral margin recovery.
**Materials and methods** In our series of 40 patients aged between 8 and 12 years, 31 were treated with placement of the plaque on the distal epiphysis of femur; in 9 cases, the site was proximal epiphysis of tibia.
**Discussion** The plaque was kept in place for an average of 14 months, according to the potential growth of the patient and the degree of correction obtained. Patients were followed for an average time of 3 years, evaluating the maintenance of the correction radiographically and clinically up to the final welding of the growth plates. In one case it was necessary remove the plaque for the occurrence of an over-correction, which using the residual growth potential returned to normal values, highlighting the reversibility and the enormous potential of the technique.
**Conclusions** The intervention with Eight-plate is confirmed as a valid alternative to osteotomy allowing an early angle correction according to the harmonious development of the skeleton in growing stage.

### Radial neck fracture in children: minimally invasive reduction and fixation

M. De Pellegrin*, A. Brivio

IRCCS Ospedale San Raffaele (Milan, IT)

**Introduction** Radial neck fractures in children are rare and, in most cases, undisplaced; the treatment of choice is usually conservative. However, for severely displaced fractures various surgical techniques have been described in literature. Many authors report a high number of complications, such as avascular necrosis, radio-ulnar synostosis, myositis ossificans and joint stiffness, following surgical procedures.
**Materials and methods** During the period from 2000 to 2009, we collected data of 7 patients (age range 6–13 years) with severely displaced radial neck fractures (Judet types III and IV), treated with a combined minimally invasive technique of closed reduction and intramedullary nailing according to Métaizeau. A percutaneous Kirschner wire inserted laterally at the fracture's level was used to obtain a closed reduction. After reduction the intramedullary wire was introduced for the osteosynthesis.
**Results** Reduction was evaluated with Métaizeau score and the results were: excellent ($n = 6$), good ($n = 1$), poor ($n = 0$). Clinical and functional results evaluated according to Tibone and Stoltz classification were: excellent ($n = 6$), good ($n = 1$), poor ($n = 0$). According to Métaizeau score, all patients had an excellent functional result.
**Discussion** Management of radial neck fractures in children is controversial when the displacement in the frontal plane is greater than 30°. The degree of displacement of the proximal fragment determines the therapeutic strategy, which may vary from casting to open reduction. We adopted the minimally invasive technique described by Feray in 1969, where the radial head is raised by means of a percutaneous Kirschner wire. This technique allows a good reduction, but not an effective stabilisation. We, therefore, associated intramedullary nailing for osteosynthesis as described by Métaizeau, which contemplates the insertion of an intramedullary nail from the distal radius to the proximal fragment.
**Conclusions** In our experience, this technique has proven to be simple, effective and without complications.

### *C29—FOOT 1*

### Clinical outcome of closed isolated subtalar dislocations

M. Marinelli*, M. Del Torto, A. Santucci, L. De Palma

Cattedra di Ortopedia e Traumatologia, Università Politecnica delle Marche (Ancona, IT)

**Introduction** Subtalar dislocation (SD) is an uncommon injury accounting for 1–2% of all dislocations. We present a retrospective study of pure medial and lateral SDs treated conservatively and discuss the pathogenesis, classification, prognostics and therapeutic aspects of SD.
**Materials and methods** Thirty patients, 24 men and 6 women (mean age 33 years; range 18–55) with closed isolated SD were treated conservatively and re-evaluated at 5–12 years. All patients were managed with immediate closed reduction under general anaesthesia. Open dislocations and SDs associated with fractures were excluded.
**Results** The mean AOFAS Ankle-Hindfoot score was 78.8. Seven patients (all with medial SDs) had an AOFAS score of 100; 14 patients (11 with medial and 3 with lateral SD) had a mean AOFAS score of 85; 6 patients (3 with medial and 3 with lateral SD) had a mean AOFAS score of 65; and 3 patients (all with lateral SDs) had a mean AOFAS score of 28. The latter patients subsequently underwent subtalar fusion, with a fair outcome. The mean AOFAS scores of patients with lateral and medial SD were not significantly different ($p = 0.05$).
**Discussion** Various factors adversely affect outcome, including type of dislocation (lateral/medial, open/closed), severity of the injury, associated fractures, length of immobilization. Management of closed isolated subtalar dislocation is by immediate conservative treatment in order to avoid or reduce the incidence of early soft-tissue and vascular complications and poor long-term outcomes due to post-traumatic arthritis, talus necrosis and subtalar joint stiffness. However, complications may still arise despite correct treatment.

### The treatment of osteo-chondral injuries of the ankle, our experience

F. Cortese*, V. Iacono, R. Regnotto, D. Mercurio, C. Zorzi

Ospedale S. Cuore (Negrar-Verona, IT)

**Introduction** Osteo-chondral lesions of the ankle are a common pathology of the adolescent and young adult in both form acute traumatic (osteo-chondral detachment) and as a sequela of "osteo-chondritis dissecans" of the talus. Incidence is about 20 cases per 100,000 with a bilaterality in 30% of cases and a prevalence of male sex. The diagnosis relies on conventional radiology but above all on MRI and CT scan. The main problem of these pathologies is that the damage is not only on the cartilage but also in the subchondral bone.
**Materials and methods** In the period 2006–2009 we treated a total of 29 cases of osteo-chondral lesions. In 11 cases we performed detached fragment fixation with absorbable pins, in 13 cases we performed the substitution of damage with biomimetic materials (12 Trufit and 1 Maioregen). In 5 cases we performed a massive osteo-chondral graft by donor (allograft). All patients were evaluated by AOFAS scores the pre-and post-operatively at 3, 6 and 12 months of follow-up.
**Results** In 23 cases we performed an osteotomy of the medial malleolus to reach the lesion. The osteotomy was then stabilized with 1 or 2 screws, we had no malleolar osteotomy nonunion. In the other 6

cases we have performed the operation without an osteotomy because the lesion was in the antero-lateral side of the talus or anteriorly in the talar neck. The pre-operative AOFAS mean score was 67 and rose to an average of 88 at 12 months. In cases where we have removed the screws have also performed an arthroscopic control.

**Discussion** The osteochondral lesions are difficult to treat; our experience has led us to prefer the fixation where the bone is still viable, otherwise we prefer biomimetic substitutes for lesions up to 1 $cm^2$ and with depths up to 6–7 mm. For larger lesions prefer using an allograft.

**Conclusions** The overall good results are not always well correlated to the MRI control images that often appear not exciting. We will probably need a longer period of follow-up to better assess the long distance arthritic changes.

**Suggested readings**

1. van Dijk CN, Reilingh ML, Zengerink M, van Bergen CJ (2010) The natural history of osteochondral lesions in the ankle. Instr Course Lect 59:375–386.
2. Giannini S, Vannini F (2004) Operative treatment of osteochondral lesions of the talar dome: current concepts review. Foot Ankle Int 25(3):168–175.
3. Ferkel RD, Scranton PE Jr, Stone JW, Kern BS (2010) Surgical treatment of osteochondral lesions of the talus. Instr Course Lect 59:387–404.

### Endolog system: a new surgical device for the correction of moderate to sever hallux valgus deformity

L. Di Giorgio*[1], S. Simone[2], G. Touloupakis[2], L. Imparato[2], L. Sodano[2], F. Vitullo[2], C. Villani[1]

[1]Ortopedia e Traumatologia B, Policlinico Umberto I (Rome, IT); [2]Scuola di Specializzazione in Ortopedia e Traumatologia, Università "Sapienza" (Rome, IT)

**Introduction** Several surgical techniques are indicated in the surgical treatment of hallux valgus moderate and severe pain. We present our experience with the Endolog system.

**Materials and methods** Our study aims to evaluate the results in the medium term (1 year) of 24 patients treated with mono- and bilateral hallux valgus (32 feet long) with the Endolog system (nail-plate) which was inserted in the mid-distal diaphysis after a distal osteotomy of the first metatarsal. The soft tissues were treated according to the present deformity. In some cases it was associated with an Akin osteotomy of the first proximal phalanx. For the pre-and post-operative clinical evaluation the AOFAS (American Orthopaedic Foot and Ankle Society score) was used. One year after surgery, the pre-operative radiographic measurements were taken on the angles: HVA (Hallux Valgus Angle), IMA (Intermetatarsal Angle) and DMAA (Distal Metatarsal Articular Angle).

**Results** The AOFAS score result gave an average of 93/100. For all the angles under consideration we saw an improvement in the average scores: HVA from 37° to 18°; I.M.A. 15° to 8°, P.A.S.A. 15° to 6°.

**Discussion** Results show that the system Endolog is a viable alternative in the surgical correction of the deformity with an intermetatarsal angle $> 30°$. The ability to perform oblique osteotomy allows the spatial arrangement of the metatarsal head to redistribute the load on the first metatarsal to correct even the axis. The mini incision reduces soft tissue trauma contributing to the reduction of post-operative pain relief. In addition, the formation of periosteal callus helps prevent early relapse.

**Conclusions** Results obtained in the medium term are satisfactory, the long-term effects should be investigated further.

### Hallux valgus surgery: comparison of techniques

R. Valentini*

Clinica Ortopedica e Traumatologica, Università degli Studi (Trieste, IT)

**Introduction** The distal first metatarsal osteotomy is indicated for the surgical treatment of mild and moderate hallux valgus deformity. For over 15 years we use the technique proposed by Boesch and modified by us with a capsulotomy and capsuloplasty to correct severe deformities. The correction is maintained with a Kirschner wire introduced from the apex of the finger. Sometimes the K-wire can create pressure skin lesions, superficial infections and post-operative stiffness. To avoid these complications, we used two different systems, Endolog system and Stoffella system. The first consists of a taproot bent titanium inserted into the shaft of M1 and secured with a screw in the head of M1 and the second by a double steel paperclip to insert in the shaft of M1 and locked with a screw in the head of M1.

**Materials and methods** From January 2009 to May 2010 we implanted 15 Endolog systems and 15 Stoffella systems. In order to compare results obtained with the above methods with those obtained with the traditional technique (K-wire), we formed three groups of 15 patients with homogeneous clinical features and evaluated with AOFAS scale. All patients were operated on under truncal ankle anesthesia and immediate loading.

**Results** The clinical results (AOFAS 92 points with traditional technique and 94 points with Endolog and 95 points for those with Stoffella) and angular measurements are similar with the three methods. With the traditional technique, the IM angle went from 13° to 8°, the MF angle from 33° to 16°, PASA from 15° to 6°. With the Endolog technique the IM angle went from 15° to 8°, the MF angle from 32° to 14°, PASA from 14° to 7°. With the Stoffella technique the IM angle went from 13° to 7°, the MF angle from 35° to 13°, PASA from 15° to 5°. The subjective satisfaction of patients operated with Endolog and Stoffella systems was better than those operated with traditional technique.

**Discussion** From our study, we have noticed a slight increase of positive results in patients treated with the Endolog and Stoffella systems in relation to the better control of the angular deformity and the subjective satisfaction for an early resumption of daily activities and the absence of skin complications.

**Conclusions** The new proposed techniques bring better results than the traditional (Boesch) because it supports a physiological post-operative period and a marked reduction of postoperative stiffness of the MF.

### The effect of cigarette smoking on bone healing after hallux valgus surgical treatment

L. Bondì*[1], R. Bondì[2], N. Aiello[1], F. Salimei[1]

[1]Ospedale S. Sebastiano Martire (Frascati-Rome, IT); [2]Ospedale S. Giovanni Addolorata (Rome, IT)

**Introduction** Delayed bone healing has been linked to cigarette smoking but only few studies have examined smoking in relation to elective foot surgery. The aims of this study was therefore to compare bone healing after bunionectomies in smokers and nonsmokers.

**Materials and methods** A total of 63 subjects, smokers and non-smokers, underwent hallux valgus correction (Reverdin-Isham and

Akin osteotomy) and were examined using the American Orthopaedic Foot and Ankle Society (AOFAS) 100-point scale concurrently with the MODEMS TM (Muscoloskeletal Outcomes Data Evaluation and Management System) Foot and Ankle Booklet and postsurgical radiographs.

**Results** Healing time after bunionectomy was 60 days in nonsmokers, 120 days in smokers with a healing time directly proportional to number of cigarettes smoked, smokers are significantly disadvantaged.

**Discussion** Smoking is shown to delay radiographic healing. We should council our patients to stop smoking perioperatively and at least during the period of their treatment to improve outcomes.

**Conclusions** Smoking is shown to delay radiographic healing. We should council our patients to stop smoking perioperatively and after surgery, however longer follow-up studies are necessary to assess how long is necessary to stop smoking during treatment to improve outcomes.

### Irreducible isolated subtalar dislocation: a report of three cases

M. Marinelli*, M. Torcianti, A. Santucci, L. De Palma

Cattedra di Ortopedia e Traumatologia, Università Politecnica delle Marche (Ancona, IT)

Subtalar dislocations are uncommon and account for approximately 1% of all dislocations. Management is by immediate closed reduction under general anesthesia. We report three cases of irreducible isolated subtalar dislocation that required an open procedure. Closed reduction failed in two patients with lateral dislocation due to interposition of the posterior tibialis tendon caused by a large tear of the flexor retinaculum. The flexor retinaculum was accurately reconstructed after the reduction. In the third case, a medial dislocation, a displaced extensor retinaculum prevented relocation of the talar head and required resection. We also discuss the mechanisms underpinning irreducible subtalar dislocations.

### *C30—FOOT 2*

### Surgical treatment of missed congenital talipes equinovarus foot

C. Faldini*[1], F. Traina[1], S. Pagkrati[1], M. Nanni[2], D. Leonetti[2], F. Acri[1], M.T. Miscione[1], S. Giannini[1]

[1]Clinica Ortopedica, Istituto Ortopedico Rizzoli, Università di Bologna (Bologna, IT);
[2]Unità Operativa di Ortopedia e Traumatologia, Ospedale Maggiore "C.A. Pizzardi" (Bologna, IT)

**Introduction** There are different conservative and surgical techniques for treatment of congenital talipes equinovarus at the birth. The treatment of club foot still is a challenge if the deformity is seen after the age of deambulation without any previous treatment. The aim of this study is to evaluate 50 patients with missed idiopathic congenital talipes equinovarus who have been treated surgically with modified codivilla technique with or without cuboid wedge osteotomy.

**Materials and methods** 92 missed talipes equinovarus feet in 50 children were evaluated (range 3–9 years, mean age 4 years). Skeletal maturity, grading & rigidity of deformity attentively were eavaluated by clinical and radiographic examination. In 48 feet the surgical procedure consisted of a postero-medial incision, lengthening of posterior tibialis tendon, reduction of talonavicular joint, lengthening of flexor hallucis longus, lengthening of flexor digitrum longus and Achilles tendon lengthening. In 44 feet instead, at the end of the intervention there was another residual deformity from excessive lengh in the lateral column of the foot that were corrected by cuboid wedge osteotomy. Post-operative treatments included short leg cast for 6 weeks following a brace during the night in talo-valgus position, insole and physiotherapy till the end of growing age. Patients were evaluated with a mean 6-year follow-up (range 3–9 years).

**Results** 6 feet had problem in the healing of skin but finally they were healed. An excellent correction was achieved in 38 feet, good correction in 30 feet, moderate correction in 18 feet and malcorrection in none of them. The second intervention was necessary in 44 feet to complete the correction of residual varus deformity.

**Discussion** Few patients affected by missed talipes equinovarus foot, have severe deformity that hamper normal activities, such as putting normal shoes and autonomous walking. Therefore the aim of surgical treatment is to put the patients' foot in plantigrade position. The modified codivilla intervention with or without cuboid wedge osteotomy is effective to correct all components deformities in one stage intervention.

**Conclusions** The modified codivilla intervention with or without cuboid osteotomy is a demanding surgical intervention, particularly in case of sever deformity. The intervention of codivilla associated with cuboid wedge osteotomy lead to a satisfactory correction of missed talipes equinovarus with a few complications.

### Chevron osteotomy with lateral release and adductor tenotomy for hallux valgus

V. Potenza*, R. Caterini, S. Bisicchia, E. Ippolito

Università di Roma Tor Vergata (Rome, IT)

**Introduction** Distal chevron osteotomy is a procedure widely performed for the surgical treatment of painful hallux valgus. The risks and benefits of a lateral capsular release and adductor tenotomy combined with chevron osteotomy are still debated. The aim of our study was to report the clinical and radiographic outcomes of this combined procedure in mild and moderate incongruent bunion deformities, with a hallux valgus angle (HVA) up to 40 degrees and an intermetatarsal angle (IMA) up to 20 degrees.

**Materials and methods** Forty-two patients (52 feet) who consecutively underwent chevron osteotomy combined with lateral release and adductor tenotomy were reviewed 24–36 months after surgery. The mean age of the patients was 53.5 (range 43–64) years. All the deformities were mild to moderate, with a mean preoperative value of 28 degrees in the HVA (range 16–40 degrees) and of 13 degrees in the IMA (range 9–20 degrees).

**Results** At follow-up, the AOFAS hallux score improved from an average of 46 to an average of 88. The HVA and IMA had an average postoperative decrease respectively of 12 degrees and 6 degrees; lateral sesamoid displacement decreased by a mean of 15%. In no case did we observe infection or nonunion of the osteotomy. In one case, painless avascular necrosis of the first metatarsal head developed.

**Discussion** Our short-term results show that distal chevron osteotomy combined with lateral release and adductor tenotomy is a feasible surgical option to address mild to moderate hallux valgus deformity, even with an IM angle between 15 and 20 degrees.

**Conclusions** Clinical and radiographic outcomes are generally good and patient satisfaction is generally high.

### Prevalence of different foot pathologies and its association with disability in adults with foot pain

L. Bondì*[1], R. Bondì[2], N. Aiello[1], F. Salimei[1]

[1]Ospedale S. Sebastiano Martire (Frascati-Rome, IT);
[2]Ospedale S. Giovanni Addolorata (Rome, IT)

**Introduction** Few studies examined the prevalence of foot pathologies in adults with foot pain. The aim of this study was therefore to determine the prevalence of pathological conditions of the feet and the impact on functionality and quality of life in subjects complain of foot pain.
**Materials and methods** A total of 1253 subjects between 18 and 75 years complaining of feet pain was examined since January 2008 to December 2010 using the American Orthopaedic Foot and Ankle Society (AOFAS) 100-point scale concurrently with the MODEMS TM (Muscoloskeletal Outcomes Data Evaluation and Management System) Foot and Ankle Booklet, the FFI (foot function index) and weight-bearing radiographs.
**Results** Hallux valgus, hammer toes and metatarsalgia were the most observed in patients with foot pain especially women, in some cases hammer toes were associated to hallux deformities, metatarsalgia and Morton's Neuroma, these patients complained of moderate foot pain and difficulty to wearing shoes and to walk for long distances. Calcanear spur and plantar fasciitis were less common and Achilles tendonitis represented the most frequent foot tendinopathy but tibialis posterior tendonitis were associated with high foot pain and related to disability in basic activities of daily living, particularly those related to standing and ambulation capacities also for short distances.
**Conclusions** Foot pathologies may differently affect quality of life in subjects complain of foot pain. Adequate assessment and treatment of foot problems may prevent foot pain and potentially reduce risk of disability.

### Endoscopic treatment of Haglund's Syndrome: indications and limits

A. Rota*, P. De Santis

Ospedale S. Pertini (Rome, IT)

**Introduction** The Haglund's Syndrome is a disease of the posterior calcaneal region and is characterized by retrotendinous bursitis, insertional tendinopathy of Achille's tendon and prominence of superior heel's angle that causes impingement with the anterior surface of the Achille's tendon. The traditional open surgery can cause complications such as delays in healing, painful scars and numbness in the heel, while the endoscopic treatment offers the benefits of minimally invasive procedures.
**Materials and methods** From 1996 to 2011, 78 patients with Haglund's Syndrome were treated by endoscopic calcaneoplasty. The patients were aged between 25 and 61 years (47 men and 31 women). All patients underwent X-ray examination which showed prominence of superior heel's angle and impingement with the anterior surface of the Achille's tendon. All patients had made conservative treatment for at least 6 months without any results.
**Results** 47 patients were reevaluated with a follow-up of 35.3 months (12–72). According to Ogilvie-Harris-Score, 21 patients had good results and 19 patients excellent, while 4 patients had fair results, and 3 patients poor results. All post-operative radiographs showed sufficient resection of the heel spur.
**Conclusions** According to our experience (maximum follow-up, 15 years), this technique has limitations; in fact contraindications to endoscopic treatment are Haglund's Syndrome with voluminous spur and tendon's calcifications. The purpose of our study was to describe and evaluate the effectiveness of a minimally invasive procedure, demonstrating the validity as well in the revisions of the open treatments.

### Our experience in ankle arthrodesis using the "panta nail"

F. Cortese*, R. Regnotto, V. Iacono, C. Zorzi

Ospedale S. Cuore (Negrar-Verona, IT)

**Introduction** We report our experience in pan-talar ankle arthrodesis (tibio-talar and subtalar joints) using the retrograde compression nail "panta-nail". This system allows less invasive surgical access to carry out the double arthrodesis and achieving a solid primary fixation by compression of the arthrodesis site.
**Materials and methods** Over the past 3 years we have used this system in 12 cases of arthrodesis in 10 patients affected byidiopathic or post-traumatic arthritis of ankle and subtalar joint, in 2 cases following the removal of prosthetic ankle. In all cases we got a proper post-operative ankle alignment. In one case we observed a postoperative infection begun 4 months after surgery which was resolved with antibiotic therapy and removal of the nail however obtaining the consolidation of arthrodesis. We observed no other vascular or infectious complications.
**Results** In all patients the consolidation of the arthrodesis was obtained in an average time of 4.3 months, the partial weight bearing with ankle orthosis has been allowed at 35 days post-op while the full load bearing has been allowed an average of 60 days. The average preoperative AOFAS score was 53 while the post-op at 6 months was 78.
**Discussion** The "panta-nail" allowed us to obtain a proper alignment of the ankle and perform an arthrodesis compression improving the chances of early stable fusion.
**Conclusions** The system used, even showing some complexity in the surgical procedure, has enabled us to achieve a safe fusion of the arthrodesis by limiting surgical exposure and with early weight bearing.
**Suggested readings**
1. Yakacki CM, Khalil HF, Dixon SA, Gall K, Pacaccio DJ (2010) Compression forces of internal and external ankle fixation devices with simulated bone resorption. Foot Ankle Int 31(1):76–85.
2. Haaker R, Kohja EY, Wojciechowski M, Gruber G (2010) Tibio-talo-calcaneal arthrodesis by a retrograde intramedullary nail. Ortop Traumatol Rehabil 12(3):245–249.

### *C31—MISCELLANY 1*

### Lumbar facet joints injections for chronic low back pain

L. Proietti*, L. Scaramuzzo, G.R. Schirò, S. Sessa, C.A. Logroscino

Università Cattolica del Sacro Cuore (Rome, IT)

**Introduction** Aim of the study was to evaluate the effectiveness of facet joints injections using glucocorticoids and local anesthetics. A cohort of 40 patients affected by lumbar facet syndrome was considered and clinical results were correlated to the sagittal contour of the spine.

**Materials and methods** Inclusion criteria were: age older than 45 years, low back pain from at least 6 months and unresponsive to conventional treatments; patients with no history of previous lumbar surgery. Exclusion criteria were: lumbar disc herniation, metameric instability, severe lumbar stenosis. Facet joints degree degeneration was evaluated using MRI according to Fujiwara classification. Sagittal contour of the spine was evaluated according to Roussouly classification. Facet joints injections were performed under fluoroscopic control with bupivacaine 0.5 cc and triamcinolone acetonide 1 cc for each facet. In all patients the treated facet joints showed different degeneration ranging between 2, 3 or 4 grades. The clinical results were evaluated with Visual Analog Scale (VAS) during the first 15 days, at 1 month and at 3 months after the index procedure.

**Results** 54.7% of the cases showed grade 2 facet joints degeneration, 33.2% grade 3, 12.1% grade 4. Twenty-eight (70%) of the 40 patients had clinical symptoms improvement in, 12 (30%) showed no benefit. The mean pre-operative VAS score was 8.3 (7–10) in the patients who had some benefit, 3.84 (1–5) at 15 days, 6.51 (4–8) at 1 month and 7.8 (6–9) at 3 months. There was a statistical significant correlation between postoperative VAS value improvement and Roussouly spine type 1 and 3 ($p = 0.003$). The benefit was more durable in patients with a degeneration grade 2 or 3.

**Discussion** The lumbar facet joints injections lead to an improvement of painful symptoms in patients with spine type 1 or 3 according to Roussouly classification and with a grade 2 or 3 according to Fujiwara classification facet joints degeneration. Clinical improvement was time-limited especially in presence of severe arthrosis.

**Conclusions** Our study showed that facet joints injections have a more effective diagnostic than therapeutic value. The procedure could, however, give a temporary pain relief in cases with an overload of the facet joints due to lumbar iperlordosis.

### Odontoid fractures in elderly patients: mortality index in surgical and conservative treatment

L. Proietti*, L. Scaramuzzo, G.R. Schirò, S. Sessa, C.A. Logroscino

Università Cattolica del Sacro Cuore (Rome, IT)

**Introduction** Odontoid fractures represent the 20% of upper cervical spine injuries. These are the most common fractures in elderly over 65 of age. Aim of the study was to evaluate the mortality index after surgical and non surgical treatment in elderly patients affected by odontoid fractures.

**Materials and methods** Since 2001 a cohort of 42 patients affected by odontoid fractures have been evaluated in a retrospective way in our Center. Comorbidity was evaluated using Charlson Comorbidity Index (CCI). Mortality was evaluated at 3 years follow-up.

**Results** There were no statistically significant differences in the mortality between the two groups at 3 years follow-up ($p = 0.18$; $p = 0.25$). CCI showed no statistically significant differences between the non surgical group (6.37 range 3–13) and the surgical group (6.88 range 3–9) ($p = 0.23$). In the surgical group survived patients showed a CCI (6.2; range 4–8) lower than the deceased patients at 3 years after surgery (9; range 6–11) ($p = 0.03$). In the non surgical group there were no statistically significant differences between deceased and survived patients at 3 years follow-up, $p = 0.21$. Cardiovascular diseases represented the most common cause of death in both groups (surgical 32%; non surgical 45%). Patients in the surgical group showed a longer survival time at 3 years follow-up than patients in the conservative group: 22 and 13 months respectively ($p = 0.001$).

**Discussion** Mortality showed a not statistically significant difference in the 2 groups at 3-year follow-up. Throughout patients in the surgical group showed a longer survival time than patients in non surgical group.

**Conclusions** In our opinion surgical treatment of odontoid fractures in elderly patients is a viable alternative and doesn't imply a higher mortality rate respect to non surgical treatment. Prospective randomized controlled studies are necessary to completely validate these considerations.

### Risk management in clinical orthopaedics: experience of application of the FMEA/FMECA technique

R. Rossi*[1], E. Montanari[2], C. Pelati[2]

[1]AUSL Ferrara (Argenta, IT);
[2]AUSL (Ferrara, IT)

**Introduction** The utility of clinical risk management in the Company Health Trust of Ferrara has envisaged a gradual introduction of analytical techniques including: RCA, Incident Reporting, Audit and FMEA/FMECA (Failure Mode and Effects Analysis). During 2010 the Health Services on six core technology were proven proactive FMEA/FMECA, one of these was the Orthopaedics Argenta.

**Materials and methods** As part of a corporate training, a group of doctors and nurses of the U.O. of Orthopaedics was progressively involved in the definition of operational processes, consultation with the team identified a critical process, "the prescription, preparation and administration of drugs". In the critical process, steps and activities were examined and the potential index of IPR (severity, probability, detectability) was identified. On IPR events with high risk corrective action plans were carried out, they were shared with the whole multiprofessional team.

**Results** We identified six priority risks (incomplete verification allergies, incomplete prescription, prevention, incomplete drug testing devices), including 2 high-risk and 4 medium risk, and appropriate levels of containment were set (pre-operative check list, review board only therapy etc.). These procedures allowed a 42% reduction of the risk index in half a year.

**Discussion** The application of FMEA/FMECA allowed, through a participatory approach, involving all members of the team, the identification of possible error situations. These possible events are now evident to everyone in our organization and everybody is involved in the implementation of corrective actions.

**Conclusions** The corrective actions became shareholders of the Company and will be introduced in other U.O. Orthopaedics.

### Surgical treatment of SLAC II: SNAC II and Preiser syndrome without midcarpal arthrodesis or proximal row carpectomy

R. Luchetti*, R. Cozzolino, A. Atzei

Rimini, IT

**Introduction** Carpal collapse resulting from SL dissociation, scaphoid nonunion or Preiser syndrome are characterized by a severe arthritic degeneration of the radio-scaphoid joint but with preservation of the radio-lunate (RL) and capitate-lunata (CL) joints. When these conditions become symptomatic, treatment of choice is resection of the first row, or midcarpal arthrodesis combined with scaphoid resection. Both alternatives eliminate a normal CL joint. If the two joints (RL and CL) are normal seems rational to keep them untouched to preserve a good wrist ROM. The objective of this study is to show the results of a new technical procedure for the treatment of these diseases with preservation of the two joints (RL and CL).

**Materials and methods** Aim of surgery is to eliminate the cause of pain in the wrist, that is, scaphoid excision and midcarpal stabilization with a strip of flexor carpi radialis (FRC) tendon. After resection of the scaphoid, the FRC tendon strip distally based is passed dorsally, around the neck of the capitate and used to support the midcarpal joint passing around the radio-triquetrum ligament. This will prevent dorsal rotation of the lunate (DISI) and the triquetrum, and prevent radial subluxation of the capitate into the scaphoid fossa.

**Results** The procedure was used in 14 cases with SLAC, SNAC, Preiser's syndrome, scaphoid bone tumor and untreated perilunate fracture dislocation of the wrist. Patients were reviewed at a mean follow-up of 21 months. Twelve patients were satisfied and returned to their previous work. Their wrist range of motion was unchanged, the grip strength increased, while the pain significantly reduced ($p > 0.01$). Post-op. X-ray showed a frequent finding of midcarpal joint narrowing with lateral subsidence of the LC joint. This, however, did not correlate with the presence of adverse functional results. Two cases failed and were treated by proximal row carpectomy after 3 months: all of them returned to previous work within 4 months.

**Discussion** This technique is less aggressive and provides faster functional recovery from surgery than traditional alternatives. Furthermore, should this technique fail, one may still go back and perform either a midcarpal fusion or a more extended carpectomy. It should be noted that this technique should not be applied in heavy manual workers.

**Conclusions** This new technique seems to be very promising in term of wrist function recovery. However before to be sure that it really works wider series and longer follow-up are needed.

### Orthogeriatric co-management of hip fractures in elderly patients: the experience of the Orthopaedic Unit of ASMN Hospital in Reggio Emilia

P. Pignedoli*[1], M. Davoli[2], F. Pellicciotti[2], G. Pioli[2]

[1]ASMN SC Ortopedia (Reggio Emilia, IT);
[2]ASMN UO Geriatria (Reggio Emilia, IT)

**Introduction** Hip fractures are a major health care problem. Most of the elderly patients with such a fracture have one or more co-morbid conditions that affect the outcome and must be managed. Epidemiological studies showed a high incidence of medical complications during hospital stays and also frequent re-admission for medical disease. Moreover, a high percentage of surviving subjects loses their abilities in daily living. A multidimensional approach mainly based on the co-management of patients between orthopaedists and geriatricians has been developed with the aim of reducing complications, length of stay, readmission rate, mortality and in general leading to lower levels of care and better function of patient at discharge. Multidimensional intervention has been shown to be successful also in postacute and rehabilitation phase improving functional recovery of patients and reducing the risk of institutionalization.

**Materials and methods** A co-managed Ortho-Geriatric Service for all patients aged 75 years admitted with a hip fracture was implemented in ASMN in Reggio Emilia in 2007. The service was set up within the Orthopaedic Unit but the model includes separate responsibilities between orthopaedists and geriatricians and a number of standardized protocols based on evidence-based procedure allowing high-level standard of care. After early rehabilitation the patients may be discharged directly to home or to rehabilitation facilities. Data were collected at admission during hospital stay and after 3, 6 and 12 months from the fracture by telephone interviews.

**Results** More than 250 patients per year were admitted with fragility hip fracture to Ortho-Geriatric Services. The co-management led to a significant reduction in surgical delays and a quicker mobilization of patients. The number of patients operated within 48 h rose from 10% to nearly 50% and more than 80% of patients, without weight bearing restriction, were out of bed 1 day after surgery. A significant reduction of acute length of stay was also observed. Both acute and 1-year mortality showed a clear reduction trend. Comorbidity and prefracture functional status were patient's characteristics significantly related to long-term outcome while, among interventional factors, surgical delay showed a negative relationship with mortality and the access to Rehabilitation Service affects functional abilities.

**Conclusions** Despite significant positive results, it is necessary to bear in mind that the co-care models represent a difficult task to be achieved and have to face with cultural, economics and administrative limits. However, the Ortho-Geriatric approach represents an important way to improve administrative and clinical results in elderly patients with hip fracture.

### Out-patient multidisciplinary practice using autologous platelet derived growth factors for the regeneration of musculoskeletal tissues

E. Costabile*[1], A. Crescibene[1], M. Napolitano[2], H. Almolla[3], S. Matera[2], G. Guido[3], F. Togo[1], M. Bossio[2], C. Giannuzzi[3]

[1]U.O.C. di Ortopedia e Traumatologia, Azienda Ospedaliera di Cosenza (Cosenza, IT);
[2]U.O.C. di Immunoematologia e Medicina Trasfusionale, Azienda Ospedaliera di Cosenza (Cosenza, IT);
[3]U.O.C. di Radiologia, Azienda Ospedaliera di Cosenza (Cosenza, IT)

**Introduction** Technological improvements and particularly the developed knowledge on growth factors paves the way to innovative procedures in social and sanitary services. The project aims at building a formal multidisciplinary practice for regenerating tissues by usingplatelet gel.

**Materials and methods** From September 1st till December 30th, 2010, 284 procedures of platelets rich plasma (PRP) gel preparation and its use in articular infiltration and 100 ultrasound guided procedures were were performed. Patients presented tendineous disease, muscular lesions or articular degenerative pathology. A pre-treatment questionnaire was administered to each patient and it was repeated at day 7, day 120 and after treatment end. Family history, remote and recent pathological history are collected from every patient, the patient's haematological and radiological exams are then evaluated. Informed consent is always obtained before applying a weekly PRP articular injection for 4 consecutive weeks. Each time the spot preparation of PRP gel was performed by drawing 8 cc of blood into a dedicated vial, Regen Fibrin polymer 2. The vial was centrifuged at 3,100 rpm for 8 min, 10% calcium gluconate is then added to the PRP which is immediately injected into the joint space, gel formation is complete within 2–7 min, as the PRP gets contact with the body temperature.

**Results** the use of platelet gel permits acceleration and improvement of healing processes and enhances tissue regeneration, these facts are evident through: (1) efficiency measurable through acceleration and quality of the healing process, prevention of set backs and improvement of patient physical, psychological state and quality of life; (2) low cost, simplicity of application and preparation on an out-patient basis; (3) absence of side effects.
**Discussion** The out-patient practice is coherent with the legislative decree 502/92, and it is based on merging innovative technology with the need to reduce health care costs, it also permits: (1) a diagnostic-therapeutic path of low assistance intensity and high professional and technical quality; (2) continuous management and monitoring of the patient with a multidisciplinary team; (3) moderate and contained hospitalization and need for drug therapy.
**Conclusions** This activity was possibly due to the close cooperation between an orthopedic surgeon, a specialist in immunohaematology and a radiologist, their multidisciplinary specialistic approach optimized duration a materials of the therapeutic process. This is intended to be a pilot project, the target is to propose autologous blood platelet derived growth factors as a treatment for musculoskeletal pathology.

### Femoral derotation in cerebral palsy

S. Reverberi*

Casa di Cura Villa Verde (Reggio Emilia, IT)

**Introduction** Derotation osteotomies in cerebral palsy are commonly stabilized with plate and screws. Bone of these patients is often osteoporotic, the use of plaques rarely consents early load because with a non-negligible frequency could occur in the mobilization or the failure of the implant. We wanted to prove the use of locked intramedullary nail in derotation osteotomy, femoral and tibial; the present study reported the results of the first 4 years of experience on the bone healing on post-operative pain, the complications observed and indications.
**Materials and methods** From 2004 to 2011 we operated 21 patients, ranging in age between 13 and 31 years, with important outcomes of PCI with torsional failure of the lower limbs. The treatment consisted in the correction of the deformity with transverse femoral osteotomy and/or locked intramedullary tibial nailing. We have not applied any immobilization. The findings, clinical and functional, were compared with those of similar osteotomies with plate.
**Results** All osteotomies healed at the final inspection. The average time of proscription of the load, in cases where the patient was ambulatory, was 40 days, while bone healing was achieved in all cases within 90 days. The functional and motor recovery was closely related with the size of the initial neurologic injury. The problems we found were minimal and rare, mainly related to bruising or bruising at the site of osteotomy or screws. We found no major complications such as infection or nerve damage or vascular disease. Bleeding was abundant in one case, while in another case we had a DVT with pulmonary embolism, fortunately resolved without problems.
**Discussion** We studied the results of femoral osteotomies performed using plate or intramedullary nail, in both cases we found an excellent healing osteotomy, but the technique with locked nailing has a lower morbidity, does not require the use of plaster and allows loads earliest limb function.
**Conclusions** The use of locked intramedullary nail allows a good correction of torsional deformities of the lower limbs in patients with cerebral palsy, reducing appreciably the time of functional recovery. In selected cases it may be a technical advantage over the traditional one, involving the use of the plates, which remains the preferred technique in patients with growth plates still open.

### Proximal femur fractures in elderly patients: the influence of comorbidity on prognosis in the short, medium and long term

L. Di Giorgio*[1], L. Sodano[2], G. Touloupakis[2], F. Vitullo[2], L. Imparato[2], C. Villani[1]

[1]Ortopedia e Traumatologia B, Policlinico Umberto I (Rome, IT); [2]Scuola di Specializzazione in Ortopedia e Traumatologia, Università “Sapienza” (Rome, IT)

**Introduction** In industerialised countries, fractures of the proximal femur in elderly patients have become a serious problem for the extent of the injury and poor prognosis. Our study aims to assess post-intervention and pre-operative mortality in patients admitted to our institute diagnosed with a fractured proximal femur.
**Materials and methods** The influence of comorbidity on prognosis in the short (1 month), medium (3 months) and long term (6 months–1 year) was assessed between July 2006 and July 2009. The comorbidity parameters considered were: sex, age, ASA class of anesthetic risk, fracture location and type of surgical treatment used, the number of previous associated diseases and the presence/absence of cognitive impairment on admission. The 238 patients (85 men and 153 women), between 65- and 99-year-old, were followed-up as outpatients and by telephone for 1 year to monitor the state of persistence in life or eventually the date of death.
**Results** The data obtained, in comparison with that of the general population in the Lazio region (ISTAT table), showed: higher mortality rate in men than women, high mortality rate for patients with cognitive impairment at admission (50%), mortality over twice as high in patients belonging to ASA classes III and IV than in patients belonging to the lower categories. By observing the time between surgery and death it was also evident that the first 6-month period was the most critical; furthermore, 63.38% of deaths occurred within the first 3 months.
**Discussion** Our results are in full agreement with the literature, with the exception of mortality related to the location of the fracture (no significant difference) and the time elapsed since surgery (high in the first 3 months, then gradually lower but not identical to that of general population).
**Conclusions** Identifying the causes that influence the mortality of our patients and being able to quantify the relevance in subsequent death enables us to improve the care for the sick, strengthen the therapeutic choices based on new evidence, and especially lay the groundwork for the development of new treatment protocols, useful for clinical and forensing decision-making.

### *C32—MISCELLANY 2*

### The treatment of tendinosis by PRP: preliminary results

S. Odella*, R. Sartore, V.E. Pedrini, F. Torretta

U.O. Chirurgia e Microchirurgia della Mano, Istituto Ortopedico G. Pini (Milan, IT)

**Introduction** The platelet-rich plasma is a fraction of plasma in which platelets are concentrated. This product is rich in growth factors (PDGF, TGF-ß, IGF-1, VEGF) that promote tissue repair. The literature reports an increase of Tenoch and collagen in tendon tissue treated with PRP in vitro [1].

**Materials and methods** Since June 2010 at the Unit of Hand Surgery 20 patients, 8 men and 12 women, affected by elbow epicondylitis or medial epicondylitis, have been treated by three consecutive infiltrations, 7 days apart, of PRP obtained through Arthrex ACP system, after local anesthesia.
**Results** Preliminary results are encouraging. We have prospectively monitored patients with DASH evaluation board for the upper limb and by the evaluation of pain VAS. All patients, after an initial peak of pain occurred in the early hours after treatment, reported a remission of painful symptoms after the first infiltration.
**Discussion** In literature, the results concerning the treatment of tendinosis by PRP are conflicting. Many questions are still open such as timing, frequency and amount for each infiltration, now is not yet completly clear the molecular mechanism of action whereby the platelet-rich plasma may act on the area damaged tendon [2].
**Conclusions** In our series we found that the treatment of tendinosis of the elbow by infiltration with PRP gives a nearly complete remission of painful symptoms in about 30 days, there were no differences in the outcome of treatment in patients whose symptoms had been present for more time. To get the final results, however, it is necessary to have a longer follow-up to assess the evolution over time.
**References**

1. Fallouh L, Nakagawa K, Sasho T, et al (2010) Effects of Autologous Platelet-Rich Plasma on Cell Viability and Collagen Synthesis in Human Anterior cruciate Ligament Injured. J Bone Joint Surg Am 92:2909–2916.
2. Rodeo SA, Delos D, Weber A et al (2010) What's New in Orthopaedic Research. J Bone Joint Surg Am 92:2491–2501; doi: 10.2106/JBJS.J.01174.

### Aquacel Ag applications in total hip and knee replacement: preliminary results

A. Schiavone Panni, S. Cerciello*, M. Vasso

Unimol (Campobasso, IT)

**Introduction** Total hip and knee replacement are extremely common procedures and more than 2 millions implants are performed each year. Infections incidence is still decreasing thanks to surgical techniques, antibiotic prophylaxis and ancillary and instrumentation improvement. However due to its devastating consequences it is still the most dangerous complication. Wound management in the very early post-operative period is often neglected even if it is important to avoid deep diffusion of superficial infections. Wound leakage or blistering is dangerous as it changes the normal wound pattern facilitating bacterial income. "Hydrofiber" technique with or without silver addiction is the most recent tool in the management of postoperative wounds. We report our preliminary experience with the Aquacel Ag device in hip and knee prosthetic surgery.
**Materials and methods** This dressing has been adopted in 40 cases: 10 primary hip prostheses, 26 primary knee prostheses and 4 revisions of total knee arthroplasties. Since the introduction of this device we changed our postoperative protocol. At the beginning wounds were treated at D1, D3, D7 and finally suture stitches were removed at day 14. Aquacel Ag dressing was done in the operative room and then kept in place until G14.
**Results** In all cases wound healing was completed at D14 with perfect skin aspect and no local complications were recorded.
**Discussion** Aquacel Ag dressing is extremely elastic and perfectly adheres during knee flexion–extension cycle. It highly reduces friction on the skin, which is responsible for blistering. This elasticity also reduces dead space, which is usually present between dressing and skin incision and is ideal site for bacterial proliferation. Aquacel dressing is waterproof and it is highly recommended when early water rehabilitation is indicated. Moreover highly absorbent fibrin layer reduces the need for dressing changement and decreases exudate coming from the wound. Exudate diffusion is dangerous; bacterial from the surrounding skin can reach it and proliferate. This dressing enhances macrophages healing properties thus reducing inflammatory response. Aquacel with silver guarantees additional specific anti-microbial activity against *Staphilococcus Aureus* and *Pseudomonas Aeruginaosa*.
**Conclusions** Our preliminary experience confirms the reliability and efficacy of this new dressing in wound management after primary total knee and hip prosthesis and in revision surgery as well.

### Absorbable scaffolds in treatment of osteochondral defects of the talar dome

E. Di Cave*, A. Alfieri, R.W. Hoedemaeker, D. Luzon, P. Versari, A. Basile, M. Ciurluini

Ospedale Israelitico (Rome, IT)

**Introduction** The purpose of the present study is to evaluate at short term follow-up, the efficacy of synthetic absorbable scaffolds in repair of isolated degree III and IV full thickness osteochondral defects of the talar dome.
**Materials and methods** 15 patients were included in the study (mean age 42.3 years). All patients were symptomatic and presented in arthroscopy an isolated III or IV degree defect (<1.5 cm). After debridement, the two layers, three-dimensional porous cylindrical implant (Trufit) were press-fit into the defect. The cylinder is composed of poly(D,L-lactide–co-glicolide) to which calcium sulphate and surfactant are added to enhance bone in-growth and make implant's surface more hydrophilic. Press-fit encourages migration of repair tissue as blood and marrow into the scaffold. At 6 months follow-up all patients were evaluated with MRI. Patient satisfaction and function were evaluated with the AOFAS foot and ankle questionnaire.
**Results** All surgical procedures were completed uneventfully. Serial ankle MRI's at 6 months showed healing of the defect with formation of good quality cartilage and integration of bone plugs in absence of adverse reactions in all patients. The AOFAS score had improved 45 points, from 38 to 83 points, with a 56.3% overall patient satisfaction rate.
**Discussion** Today several methods are available for surgical treatment of hyaline cartilage defects frequently encountered during arthroscopies. In our institute we started using Trufit absorbable scaffolds because it offers a secure support for secondary bone and hyaline-like cartilage in-growth with the advantage of being applied in one single step procedure, with less morbidity, moving patients quickly back to previous daily and sport activity.
**Conclusions** Preliminary results from our study enable us to conclude that the use of Trufit scaffolds can be a valid alternative to techniques already described in literature in treatment of medium size focal cartilage defects of the talus. Long term follow-up as well as second look arthroscopy will be needed to draw better conclusions.

### Treatment of SNAC SLAC with 4-corner arthrodesis

A. Vesnaver*[1], M. Claudio[2], A. Megaro[2], S. Galli[2], P.R.F. Ferrari[1]

[1]O.C-S. Pellegrino (Castiglione D/S-Mantova, IT);
[2]Clinica Ortopedica, Spedali Civili di Brescia (Brescia, IT)

**Introduction** The scaphoid has a fundamental role in the radio-carpal joint kinematics, and SNAC SLAC have clinical evidence of pathoarticular biomechanics. In the past, post-traumatic arthritis, secondary to ligament injuries (SLAC) and scaphoid nonunion (SNAC) were treated with symptomatic total arthrodsis, thereby nullifying the biomechanics of the wrist joint. Over the past 20 years the problem was addressed with limited arthrodesis, including the 4-corner arthrodesis. An arthrodesis success requires post-surgical pain and a limited joint function remaining as close as possible to the district contralateral joint. Although successful, this surgical procedure is not without risks, including impigement radiocarpico, infection and nonunion. The purpose of this study is the evaluation and comparison of our series with data available in the literature about the treatment and SNAC SLAC with 4-corner arthrodesis.
**Materials and methods** In the period 2003–2010, 24 wrists (including 12 out of 14 SNAC grade 3, 2 of 2 degrees, and 10 SLAC, 6 of which 2 grade 3 grade and 4) were evaluated and treated with scafoidectomy 4-corner athrodesis. In twelve cases the osteosynthesis was performed with K-wires. The mean FU was 21.8 months and in the post-operative evaluation residual function, grip strength and digital caliper, satisfaction and radiographic appearance were taken into account.
**Results** All of our patients, as well as the results of other authors, seem to be satisfying. As in literature, there is a reduction of the preoperative average of 9.18 Vas to Vas in our average FU of 1.5. The average ROM in flexion/extension is reported to be 72.1°. The average increase in strength in the post operative turned out to be 10.6 kP. Regarding complications, 5 cases were documented to the highest degree of rebound pain probably due, in two patients, not a correction of DISI. In our series, no infections were documented, four patients changed their job.
**Discussion** Evaluating the results.
**Conclusions** The results of 4-corner arthrodesis look good in the medium term.

### Isolated fractures of the capitate with dorsal displacement of the proximal pole: a case report

L. Spaggiari*, P.R.F. Ferrari, E. Niccolò

O.C. S. Pellegrino (Castiglione D/S-Mantova, IT)

**Introduction** Fractures of the capitate are rare. They are rarely isolated and more frequently associated with fracture or dislocation of carpal scaphoid (Fenton syndrome). They are probably often unrecognized, because the correct diagnosis requires further examination in addition to X-rays.
**Materials and method** We present a rare case of isolated closed fracture of the capitate with proximal pole dorsal dislocation. After open reduction and fixation with K-wires fracture healing reaching a complete range of peculiarities of the wrist was obtained.
**Results** There were no signs of avascular necrosis in 2 years.
**Discussion** Discussing the case and review of the literature.
**Conclusions** Good results can be achieved in the immediate treatment of the fracture.

### Condral lesions of the talar dome: treatment using microfracture in combination with collagen matrix scaffold

E. Di Cave*, A. Alfieri, R.W. Hoedemaeker, D. Luzon, P. Versari, A. Basile, M. Ciurlini

Ospedale Israelitico (Rome, IT)

**Introduction** The purpose of the present study is to evaluate prospectively at short term follow-up, the efficacy of microfrature in combination with a tri-dimensional collagen matrix scaffold in repair of isolated degree III and IV full thickness osteochondral defects of the talar dome.
**Materials and methods** Ten patients were included in the study. All patients were symptomatic and presented in arthroscopy an isolated III or IV degree defect (<1.5 cm). A debridement of the lesion was performed. In a second phase, by mini arthrotomy the lesion was treated with micro-fractures and the scaffold was applied. The scaffold is a three dimensional two layered membrane. At 6 months follow up all patients were evaluated by AOFAS foot and ankle score.
**Results** All surgical procedures were completed uneventfully. The AOFAS foot and ankle score showed significant improvement.
**Discussion** The described technique is relatively simple to be performed and reproducible. Applying microfractures on the surface of the lesion allows bone marrow and stem cells to migrate into the defect. The membrane holds the cells in situ forming a roof for the biological chamber. Several studies demonstrated that an extracellular matrix, particularly collagen can be used to enhance the dedifferentiation of stem cells and is able to improve the quality of tissue regeneration.
**Conclusions** Preliminary results from our study enable us to conclude that in treatment of medium size focal cartilage defects of the talus the use of microfracture in combination with a collagen matrix scaffold could be a valid alternative to techniques already described in literature. Long term follow-up as well as second look arthroscopy will be needed to draw better conclusions.

### Removal indications of fixation devices

P. La Floresta*, S. Miceli, F. Fascione, A.A. Cavero, M. Ialonardi, A. Gennarelli

U.O.C. Ortopedia e Traumatologia, Ospedale Antonio Cardarelli (Campobasso, IT)

**Introduction** The surgical removal of fixation devices is becoming one of the most frequent orthopaedic surgeries; in USA it represents the 4.9% of surgeries. These surgeries represent a considerable use of resources and they lead to intra- and post-operatory complications. There are contrasting indications for surgical removal of the fixation devices, some authors recommend the systematic removal of all the fixation devices, other suggest the removal only in case of inconveniences surely related to the implant.
**Materials and methods** The removal of fixation devices represent over the 10% of the surgical activity of our U.O. From January 2000 to December 2010, 1446 out of the 13649 surgeries that we performed were removals of fixation devices. In 707 patients the removal was indispensable (external fixation devices, Kirschner wire fixation devices, dynamizations, infections, mobilization of synthesis, pediatric age), in 739 cases the indication of treatment originated from several other causes.
**Results** In 43 cases the removal was performed because the patient was a regular soldier, in 337 cases it was performed for cluttering problems, in 198 cases for local pain, in 78 cases for psychological troubles, in 83 cases no indication for removal was apparent from the case notes. The pain symptomatology in removals for cutaneous projection was solved in the 93% of cases, while the problem of local pain without cluttering problems was solved thanking to the removal just in 35% of cases. We had a considerable complication rate (76 cases, 5%): 3 intraoperative fractures, 5 fractures occurred in the month after the surgery, 4 radial nerve damages (neuroplasy), 16 cases of impossible metalwork removal, 48 partial removal caused by the rupture of the implant.

**Discussion** Except for the necessity to remove the implant in case of infection, cutaneous projection, articular limitation, pseudoarthrosis, temporary implant or implant in pediatric age, we did not find other reasonable motivations to the removal of fixation devices. On the other hand the results of a removal in case of pain originated form an Impingement syndrome or generic implant toubles were not good as expected.

**Conclusions** The removal of fixation devices is a surgical procedure which involves expenses and risks, therefore it should be performed only in cases of real necessity. The prevention of potential but non demonstrable complications (induced fragility fractures, toxicity, allergies, tumors) such as the "tradition" or the simple request from patients for psychological reasons should not constitute a reasonable surgical indication to the removal.

### *C33—ARTHROSCOPY AND SPORT-RELATED TRAUMAS 1*

### ACT versus MSCS reconstruction technique in athlete knee and ankle coin lesions

S. Zanasi*

Villa Erbosa Hospital, GSD e Domus Medica (Bologna, IT and San Marino, RSM)

**Introduction** Purposes of this study are to compare the evolution in the reconstruction of large and coin cartilage defects in the athlete knee from the two staged ACT procedure to the one step BMDC transplant one.

**Materials and methods** Two omogeneous cohorts of 25 patients with medium–high level athletes (average age 34 ± 8 years) were included in this study for seat (MFC, LFC, patella), type (Outerbridge stage III–IV) and extension of the lesion (>3<9 cm$^2$): preoperative clinical study protocols as well as postoperative ICRS-IKDC, and 1.5T MRI assessment of cartilage with 3D-MOCART have been processed.

**Results** The preliminary clinical evaluation at an average of 12-month follow-up (range 8–24), according to ICRS-IKDC scoring system, showed for both methods, in comparison with preoperative data, a very significant improvement ($p < 0.0001$). The evaluation of the cartilage with MRI at 1.5T in 1, 3, 6, 12 months according to MOCART scoring system documented the complete coverage of the loss of substance. In the cases treated by MSCs we observed a hypertrophy of regenerated in 90% cases. X-rays films showed no worsening in any case respect of the preoperative one. The only two possible 2nd look with the use of MSCs at 12-month follow-up showed a complete reconstruction of the loss of substance with moderate hypertrophy of regenerated showing high expression of type II collagen and proteoglycans.

**Conclusions** Both techniques of cartilage reconstruction have been described to provide a tissue repair, which approximates closely to the characteristics of native hyaline cartilage. The evolution of minimally invasive surgical technique and/or arthroscopic one, new biomaterials associated with the use of BMDCs allows: the advantage of a single operative step, without requiring removal of a cartilage sample for cloning in a specialized centre and replanting after about 40 days, with consequent and important cost reductions.

### Autologous chondrocyte transplantation: perspective study at 7-year minimum follow-up

E. Kon*, S. Patella, G. Filardo, A. Di Martino, F. Perdisa, B. Di Matteo, L. D'Orazio, S. Zaffagnini, M. Marcacci

Istituto Ortopedico Rizzoli (Bologna, IT)

**Introduction** In recent times, the incidence of articular cartilage pathologies has been growing up for every group of age, due to the increasing look at body's health and consequent increasing of sport activity. Unfortunately, due to its limited regenerative potential, articular cartilage defects are hard to treat and still represent a challenge for the orthopaedic surgeon. In the last years autologous chondrocyte implants have become one of the possible choices for chondral lesions. We used a biocompatible and biodegradable hyaluronian based scaffold (Hyalograft C) for cell proliferation; its manageability allowed us to develop an arthroscopic technique to implant chondrocytes.

**Materials and methods** This arthroscopic technique was performed in our Institute since December 2000, in over 150 cases. Every patient was perspectively evaluated using the International Cartilage Repair Society (ICRS) form. Actually 83 patients reached 7-year minimum follow-up. We also used Tegner score to rate sport activity level and an MRI evaluation of the treated area.

**Results** At the 7 years follow-up we registered 8 failures, that have been re-operated for the same lesion; despite this, every score used showed good results, with significant increase if compared to presurgery. Mean subjective International Knee Documentation Committee (IKDC) score was 77.5 at the 7-year follow-up. Self-assessment of the quality of life, made by EQ VAS score, showed moreover a significant improvement to 85/100. MRI analysis, performed using the MOCART evaluation form, highlighted good filling of the defect, as testified by MOCART score.

**Discussion** This procedure of matrix-associated autologous chondrocyte implant allows avoiding the use of periosteal flaps, simplifying the surgical procedure and enabling the surgeon to perform an arthroscopic procedure, with consequent reduction in terms of morbidity of the operation.

**Conclusions** Both clinical and radiographic (MRI) evaluations, made at a medium-long term follow-up, confirm the good results previously obtained and the good durability over time of the outcome offered by this kind of bioengineered approach.

### Peroneal vincula and tendoscopy: could a proprioceptive role for vincula be hypothesized? A cadaveric and histological study

A. Marmotti*, M. Germano, R. Del Din, A. Tellini, M. Cravino, A. Tron, R. Rossi, G. Collo, L. Mosso, F. Castoldi

Università di Torino (Turin, IT)

**Introduction** Peroneal tendons possess a vascular supply through mesotendineal structures (vincula), previously related to trophic role and healing response; aim of this study is to verify feasibility of tendoscopy in evaluating peroneal tendon and vincula and to clarify histological structure of vincula and presence of nervous tissue, so formulating a hypothesis regarding their functional role.

**Materials and methods** A cadaver study was performed on 8 fresh-frozen ankles, verifying accessibility of endoscope to tendon and vincula; samples from cadaveric vincula were taken; 5 peroneal vincula biopsies were obtained from 5 patients affected by ankle instability, undergoing tendoscopy for chronic lateral ankle pain. Tendoscopy was performed for persistent pain at the posterior margin of lateral malleolus after at least 4 months of non-operative treatment. Biopsies were taken from center of pathologic vincula. Cadaveric samples were analyzed by light microscopy and immunohistochemistry (anti-human-S100-antibody, vimentin, PGP9.5, neurofilaments, p75(NGFR), myelin-basic-protein); control specimens were sural-nerve and inferior-extensor-retinaculum. Patients biopsies were analyzed by light microscopy and immunohistochemistry (anti-human-S100-antibody).

**Results** Peroneal tendons are accessible by endoscope along whole common sheath. Accessibility of endoscope along the entire length of peroneal tendon was assessed in cadaveric samples: tendoscope was pushed up to 7 cm above apex of fibula without iatrogenic lesion of tendons. Antegrade from distal portal, visualization of tendons course down to peroneal tubercle was possible. Average distance of the apex of endoscope to superficial peroneal nerve at 6 cm above the tip of lateral malleolus (or with his lateral branch, when a more proximal division was observed in the specimen) was 14 mm (DS1.04). Vincula were found in all cadaveric specimens. Histology and immunohistochemistry revealed presence of nervous fibers inside the intimate structure of peroneal vincula. Intraoperative findings of vincula lesion (thickening/scarring) were found in all patients biopsies. Immunohistochemistry of biopsies was positive for S-100-antibody consistent with small nervous fibers.

**Discussion** Tendoscopy allowed visualization of the entire length of peroneal tendons, without interference with superficial peroneal nerve course. Presence of free nervous fibers inside vincula structure is consistent with a proprioceptive role of the vinculum in peroneal tendon physiology and peritalar proprioceptive system.

**Conclusions** Our findings in patients biopsies suggest lesion of peroneal vinculum might be a nociceptive source and an important element leading, synergistically with other soft tissues injuries (i.e. joint capsule, lateral ligaments), to proprioception impairment in clinical pictures of chronic ankle instability. Thus selective excision of degenerated areas of vincula can be justified as an accessory procedure in treatment of chronic lateral pain in patients affected by chronic ankle instability.

### Implant of a polyurethane scaffold for the treatment of partial meniscal lesions

E. Bulgheroni*, P. Bulgheroni, P. Cherubino

Dipartimento di Scienze Chirurgiche e Tecnologie Avanzate (Varese, IT)

**Introduction** In recent years, the treatment of meniscal tears has become more conservative in order to avoid the degenerative changes induced by meniscal defect. In the case of irreparable injury of the meniscus or results of previous meniscectomy the use of meniscal substitutes is increasingly used. Meniscus transplantation is today well known and widely used. Despite results reported in literature are positive, many problems inherent in the procedure are not yet fully solved. Furthermore, in the case of partial meniscal defect, probably it is not correct to remove all the remaining healthy meniscal tissue to replace it with an allograft. Since 2000 it has been marketed a collagen implant to induce regeneration of a meniscus like tissue in case of a partial meniscal defect. Results reported in the medium term are satisfactory, although improvable. Recently has been placed on the market a novel biodegradable polyurethane scaffold for meniscal regeneration, with different structural and mechanical characteristic and different reabsorption time.

**Materials and methods** In 2009 we started implanting the polyurethane scaffold in case of knee pain after partial meniscectomy or irreparable meniscal tears associated with ACL reconstructions. 16 patients (17 implants) were operated on, 13 for medial meniscus injuries and 4 for lateral ones. 10 patients complained of knee pain after previous meniscectomy. In five cases was performed an osteotomy for associated knee malalignment and in 9 patients the ACL was reconstructed. All patients were clinically studied with Lysholm, Tegner and VAS scale and with MRI at pre-op evaluation, 6 months, 1 and 2 years after surgery.

**Results** Two patients reached a 2-year follow-up and 10 one-year follow-up with a significant clinical improvement. At the MRI evaluation the implant signal was evident in all the cases. In 2 cases in concomitance with the removal of HTO plate an arthroscopic re look was performed. The implant was well integrated with the surrounding tissues, with unchanged size and morphology; it was stable to the probing. Adverse reactions to the implant were not observed.

**Conclusions** The ease of implantation and the early clinical results are promising, it will take a long time to evaluate the effective non-toxic degradation products of the implant and its real chondroprotective effect.

### The behaviour of the synovial membrane in sports patients

F. Marras, L. Saturno*, F. Pisanu, P. Lisai, A.F. Manunta

Clinica Ortopedica, AOU Sassari (Sassari, IT)

**Introduction** The behaviour and morphological adaptations of the synovial membrane can be attributed to functional, mechanical and metabolic stress disorders.

**Materials and methods** The aim of this study is to examine the behaviour of the synovial membrane in the presence of the various stimuli that a sports patient's joints undergo.

**Results** In post-traumatic forms we observed a limited increase in villi that were opaque, edematous, bulky, raspberry red in colour, and covering the area. Inflammatory-forms resulting, for example, from joint instability, such as chronic ACL rupture, have an aspect of edema-proliferative synovial membrane, which can assume different aspects should it coexist with associated lesions. After ACL reconstruction surgery, the membrane assumes its role as nurturer of the ligament, and due to penetration of the synovial fringe tissues which are rich in cells and growth factors, the neo-ligament develops. Another characteristic of the membrane is that it can undergo a process of metaplasia which may evolve into joint stiffness. Synovitis of artificial ligaments is frequent in cases where of synthetic ligament revision. In sport's patients who underwent prosthetic surgery, we can see metal synovitis resulting from dispersion of metallic debris from wear of the prosthetic material. Adhesion, abrasion and corrosion are the mechanisms which are responsible for the production of this debris and which give the MS a dark colour with proliferation of different villous to different sizes, and so swelling occurs. A similar case is the infiltration of intra-articular sinovitis, where HA crystals may result from methylprednisolone injections or viscosupplementation products. A new area which requires investigation is the evaluation of inflammation and the synovial villus following infiltration with Prp.

**Discussion** A biopsy performed with arthroscopic technique, a minimal invasive surgical procedure, allows clinical signs and symptoms to be compared with macroscopic intra-articular findings, making differential diagnosis easier.
**Conclusions** In post-traumatic synovitis, the appearance of the synovial membrane is both macroscopically and histologically non-specific and similar to complex joint trauma cases, however, histological examination may inform us of how aggressive the inflammatory process is and on the need for related therapies to aid an optimal recovery in the patient.

### Extra-articular arthroscopic guided treatment for benign neoplasm of the tibial spines area

C. Zoccali*[1], G. Teori[1], U. Prencipe[1], B. Di Paola[2], E. Adriani[2]

[1]Istituto Regina Elena (Rome, IT);
[2]"Mater Dei" Medical Sport Center (Rome, IT)

**Introduction** Lesions located in tibial spines area are rare; the treatment has to follow a biopsy and a histological diagnosis, but when imaging is very characteristic for benign lesions, as chondroblastoma or osteoid osteoma, treatment can be done without histology as well. The gold-standard treatment is curettage for chondroblastoma and radiofrequency thermoablation for osteoid osteoma. Nevertheless thermoablation is having an important role in treatment of chondroblastoma of small size as well.
**Materials and methods** We describe an unusual arthroscopic technique, useful in extra-articular treatment of benign lesions in tibial spines area referring to a case of chondroblastoma so approached.
**Results** The technique is easy to be performed and allows treating the lesion; nevertheless the high intra-articular pressure made a communication between the thermoablation area and the joint, at the end of procedure. The patient is actually free of disease and in good condition at 18 months from the surgery.
**Discussion** Curettage of benign lesions in tibial spinal area needs the knee joint opening and a possible contamination so that a following local relapse can be difficult to treat. The presenting approach allows the mininvasival treatment of the lesion, thermoablation or criotherapy, without joint contamination, throughout a tibial approach where a local relapse if easier to treat.
**Conclusions** Extra-articular arthroscopic guided treatment is an adequate treatment for benign lesions in tibial spines area; moreover it permits to monitor articular surface not possible with the treatment CT guided.

### *C34—ARTHROSCOPY AND SPORT-RELATED TRAUMAS 2*

### Allograft salvage procedure in multiple ACL revision surgery

A. Ruffilli*, R. Buda, M. Cavallo, F. Vannini, A. Parma, S. Giannini

Istituto Ortopedico Rizzoli (Bologna, IT)

**Introduction** Multiple ACL revisions represent an extremely demanding surgery, due to the presence of enlarged or malpositioned tunnels, hardware, injuries to the secondary stabilizers and difficulties in retrieving autologous tendons. An anatomical ACL reconstruction is not always possible. We analyzed the results in a series of patients operated with over the top reconstruction (OTTR) and lateral extra-articular plasty to the Gerdy's tubercle (LP) using Achilles (AT) or tibialis posterior tendon (TPT) allografts.
**Materials and methods** From 2002 to 2008, 24 male athletes with a mean age of 30.8 ± 8.1 years were operated. 14 patients were soccer players. 20 of the patients had two, while four patients had three previous reconstructions. IKDC score and KT evaluation were used at a mean 3.3 ± 1.2-year follow-up (range 2–7 years).
**Results** The mean IKDC subjective score at follow-up was 81.3 ± 14.0. The IKDC objective score rated A or B in 84% of the patients. Of the 20 good results, 18 patients resumed sports activity at the pre-injury level. KT side-to-side difference averaged 3.5 mm in the TPT, versus 3.2 mm in the AT group. No significant differences were noted between the AT and TPT group.
**Discussion** ACL revision surgery is an extremely demanding surgery. OTTR, allowing to by-pass the problems related to the presence of old femoral tunnels and fixation devices, represents a valid option. The lateral extra-articular plasty improves the stability in the early post-operative period allowing a more aggressive rehabilitation. The use of allograft permits to avoid further donor site morbidity, as autograft tendons are no longer available in revision surgery. Long tendon grafts as AT and TPT are needed.
**Conclusions** Multiple ACL revision surgery is a salvage procedure. The technique described is able to guarantee good results permitting to overcome the anatomical alterations related to previous surgery but is not suitable in case of complex instability.

### Trans-tibial double bundle ACL reconstruction: 2-year experience

E.C. Cenna*[1], F. Rosso[2], S.V. Belsanti[2], E. Graziano[1], F. Galetto[1], M. Crova[1]

[1]II Clinica Ortopedica, AO CTO-M.Adelaide (Turin, IT);
[2]Università degli Studi di Torino (Turin, IT)

**Introduction** In the last few years trying to be more anatomical many authors suggested to "push down" the femoral tunnel at ten or two o'clock, using clock navigation. Although a frequent persistent glide jerk test after single bundle reconstruction could be due to an untreated periferical laxity as well related to unproper functional ACL reconstruction. The purpose of this study was to analyse our results with at least 2 years FU and discuss tricks and pittfulls in the transitibial DB technique using Volpi tibial guide and standard devices for preparing femoral tunnels and fixation.
**Materials and methods** Since 2007, 30 ACL reconstruction with single bundle (SB) technique and 30 with double bundle (DB) one have been performed at the II Orthopaedic Clinic of CTO Hospital in Turin. Single and double bundle were performed with trans-tibial technique trying to be in the center of the two ACL insertional areas for the SB and inside the areas for the DB reconstruction. All patients were prospectively checked about range of motion, stability (Lachman and Jerk test), KT-1000 evaluation, propriception and sports activities.
**Results** In SB group no failure was found, while one partial graft failure (PL bundle) in a DB patient for a second trauma at the third month after surgery was found. Range of motion was completely restored in the two groups without statistical significant differences. Average KT 1000 test was <3 mm; the pivot shift test was negative (compared side-to-side) in the 95% of the DB and 80% of the SB patients. Muscolar strength was evaluated with isokinetic test a propiceptivity with Delos and no statistically significance differences were found. The average Lysholm score for the two groups growed up from 64.9 to 88.9 without significative differences between SB and DB reconstructions. All the patients returned to their previous work and sports activities (included tennis and soccer at agonism level).

**Conclusions** The DB ACL reconstruction allows restoring anatomy with very good functional results at 2 years. The transtibial technique, with tunnel correctly positioned for area and inclination, allows performing the tunnels inside the insretional area of physiological ACL. Besides this technique is easily reproducible and allows, with some tricks, the use of standard devices for femoral trasversal fixation.

### Functional recovery after anterior cruciate ligament reconstruction in patients over 40

M. Broccatelli[1], G. De Ioannon[1], F.R. Ripani*[2], P.P. Mariani[1], F. Felici[1], F. Margheritini[1]

[1]Università degli Studi di Roma Foro Italico (Rome, IT); [2]Università “Sapienza” (Rome, IT)

**Introduction** Anterior Cruciate Ligament (ACL) rupture is one of the most common knee injuries in sports. Recently several authors found excellent clinical surgery results in young sportsmen and in individuals over 40-year-old motivated to a specific and intensive rehabilitation protocol.The aim of this study is to examine the functional recovery after the ACL Surgery in two different age groups, younger and adults.

**Materials and methods** Thirteen patients, 7 under 40 years-old (mean age 21.5 ± 4.03) and 6 over 40 years-old (mean age 41 ± 3.67) after 1 year of reconstruction-surgery for an isolated rupture of ACL, participated in this study. The groups were similar in regard to sex, stature and weight distribution, absence of meniscal or chondral lesions and post-operative rehabilitation programs, but different in age and in surgical-technique. Clinical evaluation of the patients was performed with the International Knee Documentation Committee (IKDC) and the Knee Injury and Osteoarthritis Outcome Score (KOOS) before and 12 months after surgery. Bilateral thigh muscle strength was assessed with a Kin-Com Isokinetic Dynamometer. Quadriceps and Hamstring muscle strenght, was evaluated by isometric and isokinetic measurements.

**Results** After one-year follow-up evaluation, the IKDC demonstrated no significant differences between the groups before and after operation. Furthermore the KOOS showed that the patients under 40 returned to practice sports: pre-operative value was 66.6 and post-operative 90.5; those over 40 were able to practice vigorous activity indicated a pre-operation value of 64.4 and post-operation of 92.8.

**Discussion** There was a marked improvement in the activity of daily life in sport and in the quality of life in both groups. The isometric test demonstrated that the deficit in maximal strength between healthy and operated limb was not statistically significant in both groups, and isokinetic test showed similar and excellent recovery of strength in the flexors (80% compared to healthy leg) and extensors (85%) in the operated limb in both groups.

**Conclusions** This study demonstrates that the age does not negatively affect postoperative results. Thus, the indication for the reconstruction should be based on individual factors such as level of activity or subjective feeling of instability rather than on a dogmatic age limit.

### ACL restoration with healing response: our experience

E. Cenna*[1], M. Dolfin[2], E. Balboni[2], F. Galetto[1], L. Drocco[1], M. Crova[1]

[1]II Clinica Ortopedica, AO CTO-M. Adelaide (Turin, IT); [2]Università degli Studi di Torino (Turin, IT)

**Introduction** The aim of this study was to assess the effectiveness of the method “Healing Response” proposed by Steadman in the repair of incomplete lesions of the anterior cruciate ligament.

**Materials and methods** *Casuistry*: We included in this study 53 patients aged between 13 and 56 years that underwent to surgery between 2003 and 2010, with clear signs of laxity (Lachman and Jerk + with soft stop) and X-ray imaging (MRI) and intra-operative one of ACL incomplete lesion. Of these, 40 have been re-evaluated, at a minimum follow-up of 24 months. *Description of the Healing Response*: 5–8 arthroscopic microfractures, at a distance of 2–3 mm apart, were performed at the proximal insertion of the ACL to a depth of about 3 mm, preserving the remnants of the ligament. The post-operative rehabilitation protocol included the use of a brace in extension for 60 days, with cargo and ability to perform flexion–extension movements in a closed kinetic chain 3 times a day. Considerable importance was given to the recovery of the quadriceps tone. *Evaluation criteria*: all patients were clinically evaluated with specific tests for the measurement of anterior laxity of the knee (Lachman test, anterior drawer test) and a instrumental evaluation with KT 1000. Were also completed the IKDC and the Lysholm scores.

**Results** The clinical and instrumental evaluation found good/excellent results in most of patients. Only in 4 cases traditional reconstruction of ACL was necessary for the continued of the symptoms and clinical signs of laxity.

**Conclusions** The results of this study suggest that the stimulation of bone marrow can give rise to a reparative response of the cruciate ligaments. In consideration of the good results obtained from our series, but also the broader Steadman, we should further study of this treatment option, expanding the series and increasing the period of follow-up. The patients' functional requirements evaluation and maintenance of the selection criteria is necessary. There were no significant differences regarding patient age or the time between injury and surgery.

### Anterior cruciate ligament reconstruction with Biointrafix tibial fixation: preliminary results

C. Carulli*, S. Soderi, A. Macera, C. Martini, M. Villano, M. Innocenti

Prima Clinica Ortopedica, Università di Firenze (Florence, IT)

**Introduction** Brand new absorbable devices for ligaments' tibial fixation are available on the market: among these, Biointrafix® system (Mitek, DePuy) has been released during last years but to date no publications showed any clinical results. Aim of this study is to report our preliminary experience in ACL reconstruction using this peculiar tibial fixation, evaluating the clinical results in a series of 62 consecutive patients treated at our Institution.

**Materials and methods** Sixty-two consecutive patients affected by ACL injuries were recruited for ligament reconstruction with hamstrings at our Department between July 2009 and November 2010. Surgery assisted by arthroscopy was performed by the Senior Surgeon

(MI) with Endobutton® CL/Ultra femoral fixation (Smith & Nephew), and Biointrafix® tibial fixation. All patients, after informed consent to the procedure, were evaluated in the pre-operative setting with the Knee Injury and Osteoartrhitis Outcome Score (KOOS), International Knee Documentation Committee (IKDC) score, and KT-1000: post-operative follow-up was conducted at 6 and 12 months with the same evaluations. X-rays were performed immediately after surgery, and at 6 and 12 months to assess the incidence of tibial tunnel enlargement with this device.

**Results** All subjects completed the follow-up analysis. No intra-operative or post-operative complications were registered. At 6-months evaluation, all patients were considered fully healed, and good results were noted at KOOS and IKDC sores. Particularly, objective IKDC showed level "A-normal" in 63% of the subjects, "B-nearly normal" in the remnants. KT-1000 evaluation showed a residual tibial translation between 0 and 2 mm in 65% of the study population, of 3–5 mm in the other 35%. Radiologic assessment at 6-months follow-up showed no significative tunnel variation; at 1 year follow-up, we verify non symptomatic or clinically evaluable tunnel enlargement in 4 cases. However, all scores improved significantively.

**Discussion** During last decades, many devices for tibial fixation were proposed. Improvements in biotechnology allowed the ideation of absorbable fixations that in vitro showed equivalent mechanical resistance compared to metallic systems. In particular, even its diffusion, no reports about clinical outcomes with Biointrafix are at now published.

**Conclusions** In our preliminary experience, we assessed that tibial fixation with Biointrafix showed no complications, good intra-operative strength, and all patients healed at the expected time, without any contraindications on our classical aggressive rehabilitation protocol proposed after ACL reconstruction with hamstrings.

### Arthroscopy of the peripheral compartment of the hip

F. Atzori*, A. Aprato, C. Alluto, A. Massè

S.C.D.U. Ortopedia e Traumatologia (Orbassano-Turin, IT)

**Introduction** The peripheral compartment of the hip has been disregarded for several years, and only a few surgeons stressed the clinical importance of this area of the joint. With the increasing understanding of hip pathology, the need for arthroscopic access not only for the central compartment but also for the periphery became evident.

**Materials and methods** We reviewed retrospectively 20 surgical hip arthroscopies performed using the portal to the peripheral compartment as proposed by Dienst.

**Results** Arthroscopy of the peripheral compartment of the hip performed without traction showed very low risk of potential complications, such as soft tissue injuries, neurological injuries, and cartilage damage.

**Discussion** The arthroscopic technique of the peripheral compartment of the hip without traction is safe and reproducible. The pathologic conditions of the hip involving only one compartment are rare, so it is our recommendation to combine the two techniques, with and without traction of the joint, to assess and treat diseases in both compartments.

**Conclusions** Arthroscopy of the peripheral compartment of the hip joint has become an integral part of hip arthroscopy, and helped to the understanding of the arthroscopic anatomy and pathogenesis of femoroacetabular impingement. With the improvement of this technique and the development of additional surgical instrumentation, a new field of therapeutic options has evolved.

### *C35—KNEE 1*

### The role of the PCL in total knee arthroplasty: histological assessment and radiographic correlation

V. De Santis*, V. Izzo, M. Breccia, G. Milano, C. Fabbriciani

Università Cattolica del Sacro Cuore (Rome, IT)

**Introduction** There is an ongoing debate about the role of the PCL in total knee arthroplasty. Correct joint line, physiological joint kinematics and proprioceptive function of the ligament are the main topics for PCL retaining in TKA. Aim of our study is to evaluate the status of the PCL in patients with knee arthritis by histologic analysis in face of its degenerative phenomena.

**Materials and methods** Fourteen PCL were harvested for histological evaluation from 14 consecutive patients at primary TKA. After blinded data analysis we performed a correlation with the degree of radiographic osteoarthritis (Ahlback classification). Five out of 14 PCL ligaments were excluded from the study because of their severe degeneration (Ahlback grade V) which did not allow an optimal retrieval for subsequent histological analysis. Population was composed as follows: 7 female and 2 male, mean age 71.2 years (range 65–85 years) (males = 76.5 years, females = 69.7 years). The retrieved samples were processed and subjected by hematoxylin & eosin staining. Histologic examination was carried out using conventional light microscopy. A multivariate analysis with Fisher-Test was obtained by correlating the histological grade with radiographic osteoarthritis in face of Ahlback classification. We divided samples into two groups: low (grade I-II) and high (grade III-IV) of Ahlback degeneration.

**Results** Histological analysis showed: evidence of fibrosis in all the samples, presence of histiocytes in 77.78%, lymphoid aggregates in 66.67%, vascular proliferation in 55.6%, chondral metaplasia in 55.6%, synovial hyperplasia in 55.6%, calcifications in 33%, mucoid degeneration in 22.2%, fragmentation in 22.2%, cystic degeneration in 22.2%, hemosiderin in 11.1%. Radiographic evaluation consisted in 3 patients with osteoarthritis grade II, 4 with grade III and 2 with grade IV. Histological data was then correlated with grading described by Ahlback. Our analysis showed that a high Ahlback grade (III-IV) was correlated with the presence of mucoid degeneration ($p = 0.0158$), with cartilaginous metaplasia ($p = 0.002$), with fragmentation ($p = 0.015$) and with the presence of calcifications ($p = 0.001$). These correlations were statistically significant.

**Discussion** We demonstrated that Ahlback arthritis grading could be a predictor of histological PCL degeneration, thus a valuable aid in selecting the most reliable implant. PCL in knee arthritis, as detected in our study and supported by several studies, suffers a clear involvement in the degenerative process, and these changes, especially with grade III and IV arthritis, endanger biomechanical function of the ligament.

### CR TKA, high flex and ligament balancing: 4-year follow-up

C. Zara*, S. Abusaa

Casa di Cura Villa Igea (Ancona, IT)

**Introduction** We would like to show the mid term results of a study done by using a total prosthesis with a design that enhances flexion and performance. In particular we wish to underline the values of the achieved flexion and patient satisfaction.

**Materials and methods** The study takes into consideration patients that underwent a total knee revision from January 2006 to January 2010, implanting the Permedica Prime prosthesis according to L. Whiteside's 'Ligament balancing technique'. The peculiarity of the prosthesis is in the design which improves the flexion angle whilst preserving the C. P.: the femoral distal cut has a −2 mm thickness cut-off the posterior condyles. The femoral component has an anterior thickness of 8 mm, the posterior −9 mm; the curvature of the back condyle is moved back; the back of the polyethylene is flat, with a slope of 4° combined with the 3° tibial cut makes the definitive slope 7°. Patients included in the study are 236 for 258 implants (22 bilateral implant); 152 Men and 84 Woman; Average age is 69 years (from 56 to 74 years); the pathology is always arthrosis; 177 are varum knees, 91 valgus knees; follow-up is 1–4 years.
**Results** The evaluation was performed using the Knee Society Score, closely monitoring the flexion angle value. The average preoperative score was 71.4; the average control score was 142.6; the average development was 74.8.
**Conclusions** The follow-up is still short and does not yet lead to any conclusions; however some considerations are possible. The flexion values reached by our patients are comparable to those of patients with a P.S. prosthesis; the degree of satisfaction is high (there are many more patients who do not feel the prosthesis than those who do feel it); the performance is not certainly that of a fixed plate mono-compartmental prosthesis, however it is not far from this; there were no complications regarding the LCP.

### T.T.S. femoral fixation in the recostruction of the anterior cruciate ligament

P.P. Cané*, M. Fravisini, V. Cigna

U.O. Ortopedia, Sol et Salus (Rimini, IT)

**Introduction** This study aimed at the evaluation of the clinical outcome in the reconstruction of the anterior cruciate ligament (ACL) with autologous implant of semitendinosus and gracilis tendons (STG) using Top Traction System (T.T.S.) femoral fixation.
**Materials and methods** The non-randomized prospective study included 600 patients that underwent an ACL reconstruction with the use of STG, follow-up spanned from a minimum of 3 years to a maximum of 7 years. Femoral and tibial fixation of the tendons was carried out with (T.T.S.) and a bio-absorbable polylactic–hydroxyapatite interface screw respectively. All surgical procedures were performed by the same surgeon using standard surgical procedure, and all patients followed the same post-operative rehabilitation protocol. Evaluation of the clinical result was based on IKDC and Lysholm scoring systems.
**Results** Almost all patients reported a normal or near-normal functionality recovery. The majority of medium–high level sport active patients regained function as before the injury occurred and return to sport activity.
**Conclusions** Results showed that T.T.S. femoral fixation has proven efficacy in the ACL reconstruction.

### The acute treatment of proximal ACL rupture with PRFM growth factors

F. Erschbaumer*[1], P. Di Benedetto[2], A. Causero[2]

[1]Ospedale di Bressanone (Bressanone, IT);
[2]Clinica Ortopedica, Azienda Ospedaliero-Universitaria di Udine (Udine, IT)

**Introduction** Literature documents ACL healing in certain circumstances: microfractures stimulate healing response. Growth factors stimulate ACL healing in vitro; in vivo environment is less favourable.
**Materials and methods** From January to September 2009, 12 patients were operated with arthroscopic PRFM interposition between ACL stump and femoral bone.
**Results** After 1 year patients were controlled by Rolimeter, IKDC, Lysholm Tenger activity score and MRI: 8 out of 12 knees were stable, 4 knees were unstable.
**Conclusions** Preliminary results are promising, but larger studies with longer follow up are needed.

### Combined anterior cruciate ligament reconstruction and unicompartimental knee arthroplasty

C. Terzaghi*[1], V. Ferrari[2], C. Legnani[2], W. Albisetti[3], G. Mineo[3], A. Ventura[1]

[1]U.O.S.D. Chirurgia Articolare Mininvasiva, Istituto Ortopedico G. Pini (Milan, IT);
[2]Scuola di Specializzazione in Ortopedia e Traumatologia, Università degli Studi di Milano (Milan, IT);
[3]VI Divisione, Istituto Ortopedico G. Pini, Università degli Studi di Milano (Milan, IT)

**Introduction** Medial compartmental knee osteoarthritis is a frequent occurrence which is commonly treated with proximal tibial osteotomy, unicompartmental knee arthroplasty (UKA) or total knee arthroplasty (TKA). The options for treatment of the young active patient with isolated symptomatic osteoarthritis of the medial compartment and pre-existing deficiency of the anterior cruciate ligament (ACL) are limited. Axial misalignment, over-weight, and knee instability are considered to be contraindications.
**Materials and methods** We evaluated 12 patients with primary ACL deficiency and concomitant symptomatic osteoarthritis of the medial compartment who were treated with ACL reconstruction with hamstrings and Allegretto UKA (Zimmer Inc., Warsaw, Indiana, USA), between 2006 and 2009. For 8 patients we performed one-step surgical procedure, for 4 patients we performed two-step surgical procedure. Mean age at surgery was 53.6 years. Minimum follow-up was 1.5 years. We evaluated all patients with KOOS score, Oxford Knee score, Womac score, Tegner score, clinical and radiological evaluation.
**Results** At the last follow-up, no patient had radiological evidence of component loosening, no infection, and no knee remainder instability. The subjective and objective outcome assessed with the point scales documented satisfactory average results.
**Discussion** The options for treatment of the young active patient with isolated symptomatic osteoarthritis of the medial compartment and pre-existing deficiency of the anterior cruciate ligament are limited. The UKA possesses the advantages of bone-stock preservation, reduced invasiveness and blood loss, a quicker recovery, better knee kinematics and greater cost-effectiveness in comparison to TKA.
**Conclusions** This combined surgical treatment seems to be a viable treatment option for young active patients with symptomatic arthritis of the medial compartment, in whom the anterior cruciate ligament has been ruptured.

### Biological fixation of the bone graft in anterior cruciate ligament reconstruction with bone-patellar tendon-bone: a CT study

R. Iorio*, A. Redler, A. Vadalà, L. Valeo, M. Ferretti, G. Camillieri, G. Argento, F. Conteduca, A. Ferretti

U.O.C. Ortopedia e Traumatologia, Ospedale S. Andrea (Rome, IT)

**Introduction** Anterior cruciate ligament (ACL) reconstruction is a worldwide operation. Despite the high rate of satisfactory results, failures of the graft sometimes take place even without trauma at long-term follow-up. Osteo-integration of the graft is one of the key-points for a successful outcome of the reconstruction. In this study we evaluated the integration of the bone-plug of patients operated on for ACL reconstruction with bone-patellar-tendon-bone graft (BPTB).
**Materials and methods** We prospectively evaluated 16 patients operated on with BPTB with a CT exam at a mean follow-up of 13 months, with the aim of assessing the integration of the graft inside the tibial bone tunnel. At follow-up patients were also evaluated by physical examination, international evaluation scales and KT-100 arthrometer. In order to objectively assess the percentage of graft's integration we created a radiological 4-grade evaluation scale: grade III was given in cases of complete integration of the bone-plug with the surrounding tunnel wall; grade II in cases of integration > than 50%; grade I in cases of integration < than 50%; grade 0 in cases of a total lack of osteo-integration.
**Results** Radiologically, six patients (37.5%) were classified as grade III, four patients (25%) as grade II, four patients (25%) as grade I, and two patients (12.5%) as grade 0. Clinically all patients were pleased with the operations performed and reported a satisfactory feeling of knee stability. We did not find any correlation between clinical and radiological results (*p*-value: 0.270; Chi-squared: 3.921).
**Discussion** The use of BPTB has been preferred by many authors in the past because of the reported better results over time compared to the use of hamstrings (HS). The secure and fast osteo-integration of the bone-plug was the reason for such a choice. Because of the radiological studies performed on the osteo-integration of the bone-plug in patients operated on for recurrent shoulder dislocation with the Bristow-Laterjet procedure, we decided to study what was really happening inside the bone tunnel of the knee. In particular we examined the tibial tunnel which is judged by many authors as the weak point of the ACL biomechanical fixation construct. Results of our study confirmed how the same incomplete osteointegration of the bone-plug takes place in the knee as well as at the level of the anterior glenoid neck.
**Conclusions** According to our results, the theoretical advantage of using a bone-plug graft in ACL reconstruction because of a better and faster osteo-integration should be seriously re-examined.

### *C36—KNEE 2*

### The rotating platform knee prosthesis: our experience

P.R.F. Ferrari*, A. Vesnaver, F. Piccinini, E. Niccolò

O.C. S. Pelegrino (Castiglione D/S-Mantova, IT)

**Introduction** Several studies using a wide variety of plants have documented the effectiveness and longevity of total knee replacement. The long-term success is related to factors such as the proper alignment, the correct indications and surgical technique (1.2). The continuous research and increased demand, especially in young patients has led to changes and new solutions. Changing the design of the prosthesis on the femoral geometry of the shield to improve patellar tracking, the new generation of polyethylene sterilization with gamma and the rotating platform are some of the characteristics of the PFC Sigma and LCS (Depuy Orthopaedics Italy) we have adopted.
**Materials and methods** From April 2004 to April 2009 were implanted in our Division with 392 total knee cemented moving plate (298 PFC sigma 76.02% 23.98% LCS and 94). The admission diagnosis in all cases was primary or post-traumatic gonarthrosis. In 69 cases (17.60%) had been executed one or more interventions (49 osteotomies, 12.5%).
**Results** Ten prostheses were reviewed (2.55%), 1 case for deep infection (*S. aureus*) treated with antibiotic-loaded spacer, 2 cases for aseptic loosening with subsidence of the medial tibial plateau, respectively by 1.5 and 2 AA' plant which already at 1-year follow-up was a visible line between metal ipertrasparenza back and tibial plateau (treated with revision prosthesis, 1 elsewhere). This finding was highlighted in an academic follow-up in 9 other asymptomatic cases (2.29%) currently under strict control.
**Discussion** International literature shows that the rotating platform prostheses have greater congruence with the shield of polyethylene femoral translation with consequent reduction of A/P with respect to the fixed plate prosthesis, in particular those with preservation of the PCL and symmetrical condyles.
**Conclusions** The implant depends on a moving plate for the need to better balance ligament. The sacrifice of the PCL femoral rollback, which would guarantee a good but not always a good tropism or correct voltage, is a vicarage posterior stabilization with greater knee flexion (PFC Sigma) or a polyethylene (LCS). The results of our study are in line with the international literature.

### Custom-fit cutting blocks for total knee replacement

A. Ensini*, A. Timoncini, A. Feliciangeli, P. Barbadoro, M. Busacca, S. Giannini

Istituto Ortopedico Rizzoli (Bologna, IT)

**Introduction** During the last decade computer-assisted technique have made total knee replacement (TKR) safer and more accurate: the surgical navigation system allows controlling and regulate the prosthetic components. In 2010 Medacta International and Smith & Nephew introduced a new technology in TKR based on custom-fit cutting blocks. These cutting guides for the former design is built from CT scans acquisition of the hip, knee and ankle or from MRI scans acquisition of the knee and X-ray lower-limb overview. The purpose of this study is to assess and compare the accuracy of two custom-fit technologies in terms of alignment of the customized cutting blocks, bone cuts and final prosthetic components.
**Materials and methods** This study, approved by the Rizzoli Ethical Committee in December 2010 and lasting 8 months, is expected to enroll 30 patients. Fifteen patients will undergo to CT scans of the hip, knee and ankle and as many patients to MRI scans of the knee and X-ray lower-limb overview. All the scans will be loaded on a special website and sent to the manufacturers that will produce the 3D knee model and the implant planning. The surgeon, with a personal password, will access to the web planning and will control, possibly modify, and approve it to the cutting blocks production. After 4 weeks from the approval all the patients will implant with TKR. A knee surgical navigation system will be used for recording intra-operative alignment of bone cuts as performed by means of the custom-fit cutting blocks and relevant component positioning. Prosthetic components alignments will be also assessed post-operatively on X-ray images according to a shape-matching technique. The accuracy of the custom-fit blocks will be evaluated through the comparison between pre-operative planning, and intra/post-operative data.

**Conclusions** Custom-fit cutting blocks for TKR may offer many advantages in terms of accuracy and alignment of prosthetic components. We will present the comparison between the two new technologies.

### Patient specific cutting blocks in total knee replacement: preoperative planning reliability

F. Baldo*, B. Boniforti

Ospedale S. Andrea (Vercelli, IT)

**Introduction** The main purpose in knee surgery is anatomic axis reconstruction, in order to grant the best function and the lowest failure rate. Computer-assisted surgery was a great technical improvement, but disadvantages were higher complexity, with longer operating time and costs. Given this situation a new system was developed, with patient matched cutting blocks created on preoperative imaging data. Purpose of this work is to verify anatomic reconstruction obtained and preoperative planning reliability.

**Materials and methods** Five patients with knee arthrosis were included in this study, 1 male ad 4 female, mean age 69.2 years (range 60–74 years). They underwent a baseline CT scan of the knee and scout images of the hip and ankle before surgery. For each patient images were elaborated with Medacta My Knee System, creating a preoperative planning. The surgeon inspected and validated the planning concerning the implant size, the different resection levels and femoral rotation. The planning was used to create a three-dimensional bone model on the specific patient anatomy. This bone modeling acts as the base used to create the anatomical cutting blocks. These were used during surgery for resection, fitting the patient's knee morphology without using any alignment jigs to position them. Each patient underwent a further CT after surgery, to verify correct anatomic reconstruction and preoperative reliability.

**Results** Data are still on elaboration, but preliminary ones show perfect preoperative reliability and anatomical reconstruction.

**Discussion** Looking at preliminary results My Knee system allows, respect to computer-assisted surgery, greater precision in preoperative planning, and consequently during surgery, shorter operative time, low number of instruments on operative table and lower costs. Other studies show a reduction of blood loss and lower systemic emboli.

**Conclusions** From preliminary results My Knee system allows a greater preoperative planning reliability and higher accuracy in anatomical reconstruction, with also greater advantages during surgery.

### Rotating-Hinge Knee arthroplasties: mid-term results

A. Bistolfi*, G. Massazza, F. Rosso, D. Deledda, V. Gaito, F. Lagalla, C. Olivero, M. Crova

AO CTO/M. Adelaide, Università degli Studi di Torino, Dipartimento di Ortopedia e Traumatologia e MDL (Turin, IT)

**Introduction** In knee revision and first implant surgery, in cases of significant ligamentous instability or massive bone losses, the use of constrained implants or diaphyseal stems may be necessary. Among these, the rotational hinge models, like Rotating-Hinge-Knee (RHK®) offer greater stability than the Condylar-Constrained-Knee ones (CCK®), but they also transfer higher stress on the bone interface. This study analyzes the results of 47 RHK® implant.

**Materials and methods** From 2002 to 2009, 35 revisions with RHK® model (R) and 12 cases of primary implants (P) were performed in 44 patients (33 R and 11 P) of whom 72.3% were women (average age, 72.6 years (SD ± 8.9) in group R and 55.1 (SD ± 12.7) in group P. The indications to surgery for the group R were: 25 aseptic loosenings, 4 septic loosenings, 4 instabilities and 2 polyethylene wears. The indications to surgery for the group P were: 5 post-traumatic arthritis, 4 severe valgus instabilities, 2 arthritis with rigidity and one arthrogryposis. The patellar replacement was performed in 19 cases in group R (54.3%) and in 5 cases in group P (46.6%). The HSS questionnaire was used for the clinical evaluation and the "Knee Society Roentgenographic Evaluation System "was performed for the X-ray one.

**Results** 40 patients re-entry in this study (30 R, 10 P) with an average follow-up of 44.6 months (SD ± 22.1). The scores significantly increased without differences between the two groups. In 20 cases of group R (76.9%) radiolucent lines were detected (2 cases progressive); in 5 cases of group P (50%) radiolucent lines were found (3 cases progressive). There were 7 failures in the R with a cumulative survival of 70.1% (SD ± 12.1%), and 4 failures in group P with cumulative survival of 63.5% (SD ± 14.5%).

**Discussion** Revision surgeries are often complex, with severe ligamentous instability or bone defect. Even with the two-stage technique, the risk of infection resumption is not eradicated. Due to the complexity of the cases where these models are used, the survival rate is less than other systems with partial constrain.

**Conclusions** RHK® model provides good joint stability and adhesion to bone by meta-epiphyseal cementation combined with the uncemented stems; it is indicated in cases of revision and in primary "complex" implants.

### Mid-term outcomes of unicompartmental knee arthroplasty

V. Sessa*[1], F. Forconi[1], U. Celentano[1], U. Trovato[1]

Rome, IT

**Introduction** Since its introduction, UKA has been an alternative to TKA or high tibial osteotomy for management of isolated unicompartmental knee arthritis especially in younger patients. We report that surgeons who favoured TKA over UKA for unicompartmental OA of the knee cited the significantly lower revision rates of TKA as the reason for their choice. On the other hand, those who favoured UKA had a broader basis for their preference including: less extensive surgical procedure, bone stock preservation and faster recovery. The purpose of this study was to define the mid-term clinical outcome and the survival rate of the medial UKA with minimal invasive surgical approach.

**Materials and methods** The study included 83 patients operated on consecutively from 2004 to 2008 by the same senior surgeon. The age of the patients ranged between 48–76 years. Every patient enrolled in the study responded to Carr criteria. Mid-term clinical evaluation was effected assessing the Knee Society Scoring System.

**Results** Five patients were lost at follow-up, leaving 78 patients (92 knees) with an average follow-up of 3.7 years (range 3–7 years). At the latest follow-up, the survival rate was 94.6%; Average total knee score was 171 (range 94–200). Eighty-six percent of the patients indicated satisfaction with the procedure. Five knees failed requiring revision.

**Conclusions** This study confirms UKA as one of the most feasible surgical option to approach unicompartmental OA especially in younger patients with reproducibles and predictable outcomes. Although early midterm studies cited a high rate of satisfactory results after UKA, long-term survivorship and outcome continue to be a concern. Although these concerns are not without foundation, they have to be addressed with recent advancements in prosthetic component design, instrumentation and surgical technique, and appropriate patient selection.

### Endo-model implants for knee revision surgery: long-term results

A. Bistolfi*, G. Massazza, F. Rosso, F. Lagalla, F. Galetto, C. Olivero, M. Crova

Dipartimento di Ortopedia e Traumatologia e MDL, AO CTO/M. Adelaide, Università degli Studi di Torino (Turin, IT)

**Introduction** In revision knee arthroplasties surgery the use of constrained implants or diaphyseal stems to compensate ligamentous instability and/or bone losses is often necessary. According to some authors, the models with rotational constraint may have early loosening because of the high stress at the bone-prosthesis interface. This study analyzes the results of a series of Endo-Model® (Link) knee arthroplasties for revision surgery.

**Materials and methods** 53 Endo-Model® were implanted for revision surgery in 49 patients: 33 women, 17 men, with an average age of 69.6 years (SD ± 6.5). Indications for surgery were 20 aseptic loosenings, 13 septic ones, 8 wears/breakages of the polyethylene, 3 femoro-tibial instabilities, one dislocation, one peri-prosthetic fracture. In 7 cases a single component was replanted (revision of previous Endo-model): 2 polyethylenic-hinge ruptures, 2 dislocations, 2 tibial wears, one femoral stem rupture. The HSS questionnaire was used for clinical evaluation, while the "Knee Society Roentgenographic Evaluation System "was used for radiographic evaluation.

**Results** 32 patients (36 prostheses) completed the study (65.3%; 7 revision of one component) with an average follow-up of 155.2 months (SD ± 40.1). All HSS scores significantly increased. In particular, the total score increased from 58.4 (SD ± 10.8) to 85.5 (SD ± 10.1) and the range of motion from 81.3° (SD ± 11.2) to 102.6° (SD ± 143). There were 11 cases of failure (27.7%): 2 amputations, 4 revisions of the entire system and 5 of a single component. In other 9 cases rupture of the polyethylenic-hinge was detected (25%) and, among these, 3 patients underwent to revision surgery. The cumulative survival rate was 80.4%. In 8 cases, progressive radiolucent lines (22.2%) was detected.

**Discussion** Endo-Model® provides good joint stability and resistance for the massive cementation and it is indicated in cases of revision, first "complex" implants and in cases with ligamentous instability and severe bone loss. In addition, modular solutions and press-fit stems that may facilitate implantation are currently available. However, because of the complexity of the cases in which this model is used, the survival rate is less than in other systems with partial hinge or the first implant.

**Conclusions** Endomodel implant can secure good results for revision surgery at medium-long term.

### Early experiences in knee prosthesis with the visionaire system

E. Bonicoli[1], S. Marchetti*[1], L. Andreani[1], A. Baluganti[1], D. Caramella[2], M. Lisanti[1]

[1]Clinica Ortopedica Universitaria (Pisa, IT);
[2]Dipartimento di Oncologia, dei Trapianti e delle Nuove Tecnologie in Medicina (Pisa, IT)

**Introduction** In the last few years the knee prosthesis has been in continuous development as concerning both the design and the surgical technique. Despite the experience and the improvement of the toolkits, the defects of alignment and implant placement are still frequent. For these reasons we have started to use the Visionaire System (Smith/Nephew), which allows the creation of patient-specific cutting masks that can be used in the operating theatre throughout the development of preoperative data.

**Materials and methods** Our experience began in January 2011: we selected some patients with varus deviation of the knee without serious associated diseases. Three weeks before the operation, each patient performed X-rays in the upright position and MRI of the knee (1.5 Tesla) [STEP 1]. All the above mentioned tests together with the patient data are sent to Smith/Nephew laboratories in Memphis (USA) for the reworking [STEP 2]. A week later, the digital pre-operative planning was sent in which the surgeon could assess the osteotomy lines and the size of the components: the system was interactive and all changes were communicated to the laboratory that would make changes based upon feedback [STEP 3]. Once the planning time was finished, the data were used by the Smith/Nephew laboratories in order to create ready-for-use patient-specific cutting templates that were shipped a few days before surgery.

**Results** From the first implementations of the Visionaire system we have verified a high level of precision in all cases. Besides, we have seen a deviation from the digital planning less than one mm. Since the whole part of the evaluation of this prosthetic alignment (axis, cuts, balancing…) is made during the pre-operative phase, by using this system we have observed a reduction of the operating time. All this, together with the elimination intramedullary alignment system, resulted in decreased blood loss.

**Conclusions** On the basis of our first experiences, we believe that the Visionaire System represents an improvement compared to current surgical techniques. The possibility of having cutting custom templates based on a careful preoperative planning, apart from reducing bleeding and surgical time, may reduce the number of failures in the long run. Nevertheless, to quantify the potential benefits and disadvantages of the Visionaire System, further studies and data collection are needed based on a larger number of patients.

### ROUND TABLES

### *BLOOD CONSERVATION STRATEGIES: IMPORTANT TOOLS*

### Platelet rich plasma in Achilles tendon repair: in vivo study on Wistar rats

A. Busilacchi*, M. Del Torto, D. Ramazzotti

Clinica Ortopedica, Università Politecnica delle Marche (Ancona, IT)

**Introduction** Platelet rich plasma has nowadays a wide diffusion in orthopaedic and traumatic pathology. Is well known that the growth factors (GFS) released through the platelet lysate are involved in the healing process, stimulating the proliferation of resident cells and structural proteins. Goal of the present study was to assess safety and efficacy of PRP in healing of Achilles tendon subtotal tears, using an animal experimental model.
**Materials and methods** For the experiment 32 Wistar rats were used. The PRP was obtained by killing 2 animals. After the required centrifugations, the precursor of the final PRP was activated with thrombin 20 U/ml and irradiated at 25 Gy.
**Surgical procedure** In both the posterior limbs: para-Achilleal medial incision, sectioning 2 of the 3 fascicles of the tendon. After randomization one tendon was treated with a standard dose of PRP, while the contralateral was treated with a control solution (1CPD:9saline). Randomly animals were killed in $CO_2$ chamber at day 40 and day 60. Each specimen underwent histological and hystomorphometric analyses. Some specimen were evaluated with Micro-CT analisys. Finally the concentration of 10 GFs contained in the PRP was assessed using monoclonal antibodies (in triplicate procedure).
**Results** Light microscopy and hystomorphometric analyses showed an higher collagen synthesis and fibroblastic like cells proliferation in treated tendons in comparison with controls at 40 days. After 60 days these differences were not so meaningful. Moreover, a neo-angiogenesis phenomenon around the lesion area was observed in treated ones. During the study no adverse (AE) or side effects (SE) were observed, neither calcifications, neither metaplasic ossifications. Micro-CT data show in treated a non-homogeneous healing tissue, if compared with native tendon.
**Conclusions** The results of this study complemented with the conclusions achieved in literature sustain that PRP stimulates tenocytes proliferation and collagen production, mostly in the postoperative-period, although it has no meaningful effects in long term on the quality of the healed tendon. Even if we did not observed any AE or SE, we cannot assess that in a long term PRP is completely safe.

### TRA.DAT.E. (Transfusion Data in orthopaedic Election) study. Multidisciplinary approach for elective orthopaedic patients: preliminary results

I. Beverina*[1], U. Fusco[2], L. Micheletto[3]

[1]Centro Trasfusionale, Az. Osp. Ospedale Civile di Legnano (Legnano-Milano, IT);
[2]U.O. Ortopedia, Presidio di Tradate, Az. Osp. Ospedale di Busto Arsizio (Tradate-Varese, IT);
[3]U.O. Anestesia e Rianimazione, Presidio di Tradate, Az. Osp. Ospedale di Busto Arsizio (Tradate-Varese, IT)

**Introduction** The reduction of blood loss in orthopaedic surgery can be achieved through multiple approaches, with the involvement of several professionals: anesthesiologists, surgeons, transfusionists. Data were collected in Tradate Hospital, where the corresponding author was chief of the Blood Transfusion Center during the period under study.
**Materials and methods** 1075 surgeries of hip and knee arthroplasty (performed during the period 2004–2009) were evaluated for different parameters, having as a goal the reduction of homologous transfusion (HT). The tools used for this purpose were: (1) issue of Guidelines for Good Use of Blood; (2) tailored autologous blood donation; (3) systemic use of tranexamic acid; (4) treatment of preoperative anaemia; (5) intra-articular injection of tranexamic acid in knee surgery.
**Results** Between 2008 and 2009, 6.0% of patients were transfused, compared to 27.3% between 2004 and 2005. Post-operative blood recovery was never used. To date we collected data regarding 748 medial charts: a preliminary statistical analysis showed a significant reduction in blood loss in patients treated with antifibrinolytic (374 patients for each group—$p < 0.000$) without increasing the risk of thrombosis. It is still assessing the impact of the intra-articular injection of antifibrinolytic drug.
**Discussion** A multidisciplinary approach is effective in reducing the need for transfusion in orthopaedic surgery.
**Conclusions** The antifibrinolytic drug should be part of a blood sparing strategy in this type of surgery. Final results will be presented and discussed.

### Using a fibrin sealant in total knee replacement

A. Spinarelli*, V. Patella, V. Pesce, G. Vicenti, A. Martucci, A. Monno, B. Moretti

Clinica Ortopedica e Traumatologica II, Azienda Ospedaliero.Universitaria Policlinico (Bari, IT)

**Introduction** The operation of total knee arthroplasty is burdened by a major intra and post-operative blood loss, which often requires the use of blood transfusion. We studied different methods of conservation of blood mass, but the ideal solution is to reduce blood loss. To do this you can take advantage of a surgical fibrin sealant derived from a cryoprecipitate of human origin which, sprayed on the surgical site, mimics the final stage of coagulation thus reducing bleeding.
**Materials and methods** In the II Department of Orthopaedics and Traumatology of the University Hospital Policlinico of Bari "Aldo Moro" a surgical fibrin sealant (Quixil®) was used from 2008 to 2010 on 110 patients undergoing surgery for total knee arthroplasty. Patients were divided into two groups: *group A*, 64 patients receiving standard intraoperative hemostasis + 10 ml of Quixil®; group B, 46 patients receiving only the standard intraoperative haemostasis. The two samples were homogeneous for age, sex, weight, height and race. All operations were performed with tourniquet, always by the same team using the same technique, patients were given the same thromboprophylaxis with Clexane® 4,000 IU 12 h before surgery and then every 24 h until the 35th day post-surgery. The parameters evaluated were: the post-operative blood loss, decrease in haemoglobin and transfusion requirements.
**Results** The post-operative blood loss was estimated at 450 ± 261.41 ml of blood in group A and 898 ± 423 ml of blood in group B with a mean difference of 448 ml. The decrease of haemoglobin (Hb) in group A was 1.9 ± 1 g/dL versus 4.2 ± 1.7 g/dl in group B. In 32 (50%) patients of group A a transfusion of 1 unit of concentrated red blood cells (CRBC) was required while in 5 (8%) patients a transfusion of two units. In 46 (100%) patients of group B a transfusion of 1 unit of CRBC was required while in 23 (50%) patients a transfusion of two units. The results for the three parameters considered were statistically significant ($p < 0.001$).

**Conclusions** The use of Quixil® in total knee replacement reduces postoperative bleeding and use of blood transfusions, maintaining high levels of Hb. In our study we found a reduction of about 50% of blood loss and use of transfusions.

### Effect of preoperative blood donation and intra- and postoperative blood recovery on homologous blood transfusion requirement in joint replacement

L. Pisano*[1], A. Malatesta[2], D. Pastorelli[2]

UOD Ortopedica, Azienda Ospedaliera San Giovanni Addolorata (Rome, IT);
[2]Servizio Immunotrasfusionale, Azienda Ospedaliera San Giovanni Addolorata (Rome, IT)

**Introduction** The risks of homologous blood transfusions are known (transfusional reactions, HCV, HIV, coagulapathy from consumption of platelets and coagulation factors). We can accept these risks in emergency but not in election surgery. Are the methods for blood recovery in hip and knee replacement useful to decrease the incidence of homologous transfusion? To which one we give preference?
**Materials and methods** To answer these questions, we conducted a prospective study examining the clinical data of three groups of patients: (1) patients with autologous blood pre-deposit; (2) patients with pre-deposit and blood salvage during and/or after surgery; (3) patients with blood salvage during and/or after surgery. We reviewed the clinical data of 128 patients in the period of January to December 2007 who were submitted to total primary hip or knee replacement, evaluating: (1) the levels of preoperative Hb at the day of surgery, the first, second, third, fifth and the 30th day; (2) he percentage of patients that received auto-transfusion; (3) comparison of intraoperative versus intra-postoperative blood salvage; (4) the incidence of homologous blood transfusion.
**Results** The results obtained were as follows: (1) 27 patients (21.1%) received homologous blood transfusions, out of whom 13 patients were transfused with more than one bag (max 3); (2) in cases of only intraoperative blood salvage the quantity was less than 100 ml and not transfused; (3) in 27 out of 30 cases of intra- and postoperative blood salvage, recovered blood was transfused (90%); (4) 30 patients were prepared with autologous blood predeposit (17 with two bags) and of these only 4 patients (13.3%) received homologous blood transfusion.
**Discussion** The application of homologous blood transfusion in prosthetic hip and knee implant surgery reported in the literature is related to preoperative Hb levels. We observed that blood saving technique permits the application of less homologues blood in patients with similar levels of preoperative Hb.
**Conclusions** The application of homologous blood transfusions in our series (mean age 73 years, and prevalence of non-cemented implants at greater postoperative bleeding) is 21%. The postoperative recovery technique has the highest efficiency.

**Suggested readings**

1. Ayers DC, Murray DG, Duerr DM (1995) Blood salvage after total hip arthroplasty. JBJS 77:1347–1351.
2. Grosvenor DMPH, Goyal VBS, Goodman SMD (2000) Efficacy of postoperative blood salvage following total hip arthroplasty in patients with and without deposited autologous units. JBJS 82:951–954.
3. Garvin KL, Feshuk CA, Sekundiak TD, Lyden ER (2005) Blood salvage and allogenic transfusion needs in reviision hip arthroplasty. Clin Orthop Related Res 441:205–209.

### How many patients really benefit from autologous blood donation in hip and knee elective prosthetic surgery? Mathematical simulation and cost analysis associated with different modalities of selection

I. Beverina*

Centro Trasfusionale, Az. Osp. Ospedale Civile di Legnano (Legnano, IT)

**Introduction** Autologous Blood Donation (ABD) has been, for several decades, the most frequent procedure used to minimize the likelihood of homologous transfusion (HT) in elective surgery, especially orthopaedics. Currently, the crucial role of ABD in achieving a reduction in HT is being questioned.
**Materials and methods** Data were collected in Tradate hospital, where the author was head of the Blood Transfusion Center during the period under study. In our Institution ABD is performed if Predicted Blood Losses are <1 unit, according to Mercuriali's algorithm (Curr Med Res Opin, 1996). Once the data had been collected (*Group A*), two scenarios were mathematically simulated: no patients (pts) with ABD (*Group B*) and all pts with ABD (*Group C*), and both Group B and C were compared to the real situation (*Group A*) respecting the Absolute Risk (AR) for each theoretical group of receiving HT. The costs were also compared, considering the official price for each haemocomponent in Italy.
**Results** In the period 2005–2008 we selected 452 pts undergoing hip or knee prosthetic surgery. Our simulation shows: (1) a comprehensive execution of ABD (*Group C*) is not justified, since it determines increased costs (95,074 USD vs. 48,576 in the *Group A*) compared to a slight reduction of the AR of HT (−4.0%); (2) the exclusion of all pts from ABD (*Group B*) results in a slight increase in AR (+6.9%) with lower costs (23,334 USD); (3) the selection method so far adopted has proved to be a good compromise between cost containment and reduction in AR of HT; (4) the AR increase is not so significant, compared to a halving of costs, to justify the use of ABD in pts with hematocrit > 38%.
**Discussion** Our records show that a serious revision of ABD in orthopaedic surgery is required.
**Conclusions** An ABD program tailored on the individual patient is effective in containing costs, without bringing a significant increase in transfusion risk. On the ground of our data, since 2010 we began an ABD program only for pts with hematocrit > 38%.

### Study of efficacy and safety of "double unit" red blood cell concentrate by apheresis: comparison with collection of 1 or 2 units of whole blood

I. Beverina*

Centro Trasfusionale, Az. Osp. Ospedale Civile di Legnano (Legnano, IT)

**Introduction** Autologous Blood Donation (ABD) is usually performed by collection of 1 or 2 units of autologous whole blood (WB). The main limitations of this approach are the extent of maximum volume collected (generally not exceeding 450 cc each session) and the "post-withdrawal hypovolemia". Current apheresis devices allow us to collect in a single session two units of red blood cells (2RBC), without the limits mentioned above. We compared, in terms of efficacy and safety, the data of 27 pts who predeposited 2RBC with pts who donated 1 (1WB) or 2 (2WB) units of 350 cc of WB (23 pts for each group).

**Materials and methods** Data were collected in Tradate hospital, where the author was head of the Blood Transfusion Center during the period under study. We performed 2RBC collection by the MCS® + Haemonetics device, with a target of 410 cc of RBC volume or as to maintain the post-apheresis hematocrit (Hct) > 29%. We used Mercuriali's algorithm (Curr Med Res Opin, 1996) to determine the number of units of WB to be taken. The interval between donation and surgery has never been less than 21 days for both types of procedure.
**Results** Data are consecutively presented, as mean value, for patients who have donated 1WB, 2WB, 2RBC. Pre-Hct: 40.3%—38.3%—38.1%; RBC volume collected: 148—259—340; days between donation and surgery: 25—24—26; post-donation Hct (%): 36.6—32.1—29.3; RBC mass recovery (g): 85—131—254 corresponding to 0.5—0.8—1.5 standard units of homologous blood ($p < 0.00$ one way ANOVA test for the last three comparisons). We found an inverse correlation between RBC mass recovery and post-donation Hct ($r = -0.68$). We recorded one vasovagal reaction for each group in 1WB and 2WB, none in group 2RBC.
**Discussion** 2RBC has proven to be extremely well tolerated by patients. Regarding the extent of recovery of RBC mass, the study confirms that a rapid achievement of hematocrit values close to 30% is the only non-pharmacological method to stimulate erythropoiesis consistently. The increased production of RBC brings a benefit in terms of cost-effectiveness.
**Conclusions** In our Institution the autologous 2RBC, unless inadequate venous access, is nowadays the modality of choice for ABD.

### The hemostatic system "QUIXIL®" in hip prosthesis

T. Scuccimarra*, G. Guerra, D. Vanni, C. Colucci, V. Salini

Clinica Ortopedica e Traumatologica, Università degli Studi "G. D'Annunzio" (Chieti, IT)

**Introduction** Major orthopaedic surgery is associated with blood loss intra-and post-operative, with a marked influence on stroke recovery and rehabilitation and, in particular cases, ischemic heart, brain or kidney damage. Occasionally religious or organic issues (immune sensitization, on congenital or acquired basis) that prevent the blood transfusion coexist. The local application of fibrin glue or as a spray, in order to directly reduce the intra-and postoperative bleeding, has already yielded interesting results in general surgery, and encourages their use in orthopaedic surgery.
**Materials and methods** We examined 50 patients aged between 55 and 85 years included into 2 groups. The operation of total hip arthroplasty was performed by the same surgeon who implanted by direct side, the same type of prosthesis. We have excluded patients with severe cardiovascular, metabolic or autoimmune diseases, which would have affected the homogeneity of the sample. The following parameters were considered: haemoglobin (hb) and pre-operative hematocrit; loss intra-and post-operative; hb postoperative (12, 24, 48, 3rd and 5th day), units of transfused blood.
**Results** In patients treated with Quixil®, we demonstrated better control of blood loss, reduced postoperative bleeding (Hb curve down less pronounced) with decreased incidence of hematoma and pain in the operated region.
**Discussion** Quixil® is a fibrin hemostatic composed of human thrombin, human fibrinogen and tranexamic acid, free of bovine thrombin and therefore immunization risk-free. When mixed, the components form fibrin rapidly and, creating cross-links with the collagen fibers of the tissue, create a stable clot.
**Conclusions** Quixil® was effective in reducing bleeding and the need for blood transfusions. According to this study total hip arthroplasty can be performed with a significant reduction in use of blood transfusions, perpetuating thereby the action of clotting fibrin placed in the operative field and reducing the chance of bruising and blood loss from drains, and therefore the need for transfusion.

### Reduction of blood loss using tranexamic acid in total knee and hip arthroplasties

F. Specchiulli, M. Rendina*, M. Berardi

Cattedra di Malattie dell'Apparato Locomotore, Università degli Studi di Foggia (Foggia, IT)

**Introduction** Adverse transfusion reaction, increased cost of safe blood, the refusal of transfusion on religious grounds have led to the development of new methods of controlling postoperative bleeding.
**Materials and methods** We carried out a randomized study in 80 patients who underwent a total hip and knee replacements. Patients were assigned into one of the following groups. The patients who received tranexamic acid (500 mg in two bolus doses) comprised 20 who underwent THR (*group A*) and 20 who underwent TKR (*group C*), while 20 THR patients (*group B*) and 20 TKR patients (*group D*) had no pharmacological intervention (*control group*).
**Results** Total blood loss was significantly reduced in patients given tranexamic acid in both the THR and TKR groups, with a reduction of 56.2 and 49% respectively. The number of units of blood transfused was 32 in group A versus 50 units in group B, with a reduction of 37.1% ($p = 0.008$), and 28 in group C versus 46 in group D, with a reduction of 39% ($p = 0.006$). No severe complications, such as venous or pulmonary thrombosis, were noted in any of the patients who received the agent.
**Discussion** The literature shows discordant reports of efficacy of t.a. treatment in THR patients. Most studies agree that the tranexamic acid is useful in determining a significant reduction in postoperative blood loss. Our data appear in agreement with these authors.
**Conclusions** In conclusion, this finding suggests that tranexamic acid reduces postoperative blood losses and transfusion requirements in patients undergoing THR and TKR surgery. Therefore, we recommend its use for controlling postoperative bleeding.

### *BONE BANKING: NATIONAL AND REGIONAL ORGANIZATION*

### A-Flex Grafton in hip reconstruction

A. Teti*, M. Papalia, F. Favetti, F. Casella, F. Falez

UOC Ortopedia e Traumatologia, Ospedale S. Spirito, Azienda USL RM/E (Rome, IT)

**Introduction** A-Flex Grafton is a disk-shaped filler for bone defects, made of demineralized bone matrix (DBM): it is available in 60 mm diameter and it allows for simple manipulation to fit bone voids in acetabular wall, both for primary and revision hip surgery. Aim of the present study is to confirm the effectiveness of this scaffold, on the basis of results achieved with our first 15 cases. We will also try to settle pre-operative indications and intra-operative parameters to a proper use of this bone-filler.
**Materials and methods** We used A-Flex Grafton in 15 cases, 3 primary hip reconstructions and 12 revision procedures (6 of them for aseptic loosening, 6 during two-staged revision for septic loosening). The scaffold was positioned directly on the acetabular wall, before cup implantation, and in all revision procedures an amount of morcellized bone grafting was impacted to improve bone-stock reconstruction. A clinical and radiological follow-up was performed at 1, 2, 3 and 6 months post-operatively.

**Results** We detected an adequate osteo-integration in all 15 cases (100%) at 6 months, both in primary and revision procedures (even in case of impaction bone grafting). No complication was observed, such as infection or loosening.

**Discussion** A-Flex Grafton was launched in 2009, and the short period of investigation does not allow conclusive responses. Previous studies demonstrated osteoinductivity and osteoconductivity of DBM, in particular in term of bone induction compared to other bone matrices. Our results suggest the effectiveness of A-Flex supporting cup fixation and bone chips integration in both in primary and revision surgery.

**Conclusions** A-Flex is an easy to be used and manipulated bone inductive scaffold, without supportive function; it is able to fill bone voids in acetabular reconstruction (associated or not to bone chips impaction) and to promote component fixation and bone chips integration.

### *INTERVENTIONAL RADIOLOGY*

### Calcific tendinopathy of rotator cuff: comparision between ultrasound (US)-guided perforation and lavage using a two-needle technique and arthroscopic management

A. Di Francesco*, A. Conchiglia, L. Zugaro, C. Masciocchi, S. Flamini

A.S.L. N° 1 Avezzano-Sulmona-L'Aquila, Ospedale Civile S. Salvatore (L'Aquila, IT)

**Introduction** We report a prospective study comparing percutaneous ultrasound and arthroscopic surgical treatment in the treatment of calcific tendinopathy of the rotator cuff.

**Materials and methods** From June 2007 to June 2010, we enrolled 40 patients with symptomatic calcific tendinopathy of the rotator cuff (larger than 2 cm × 2 cm), with a median follow-up of 1 year. Patients were studied radiologically with X-rays, ultrasound and MRI (number of calcification, size, location, state of maturation) and clinically evaluated with the Constant-Murley scale (pain, motion and strength) and randomly assigned to *group A* (mini-invasive ultrasound treatment) and *group B* (surgical treatment Arthroscopic). The evaluation of patients in group A and B was performed before treatment and 1 month and 1 year later. Particular attention was placed on the characteristics of radiological density of calcification. Both the ultrasound-guided procedure and arthroscopic surgical treatment were performed by a single operator for each treatment. The procedure consists of US-guided technique with double needle with local anaesthetic bursal space. The arthroscopic procedure was performed in regional anaesthesia with interscalene plexus, emptying of the calcification and sometimes with arthroscopic suture.

**Results** *Group A*. Control at 1 month: 16 patients with a complete regression of joint pain and complete recovery of daily activities and range of motion; in 4 patients, partial reduction of the articular symptoms with a reduction range of motion of 30%. Control at 1 year: in 2 patients was observed by MRI tendon injury.

*Group B*. Control at 1 month in 17 patients with complete regression of joint pain and complete recovery of daily activities and range of motion; in 2 patients partial reduction of the articular symptoms with a reduction range of motion of 30%. Control at 1 year in 1 patient was observed by MRI tendon injury.

**Discussion** The pathology of calcific tendinopathy of the rotator cuff is still difficult to interpret. By this work we want to understand the cause of these invalid results, and also to seek a treatment with fewer risks and better results.

**Conclusions** By now the treatment of this disease through the echo-guided procedure is minimally invasive and very low costing. No differences were observed in the two groups regarding the results also with a tendon suture in arthroscopic treatment. The case at 1 year-control, in each group with tendon injury, is attributable to the state of maturation of calcification, this calcification had a high radiological density (dense and hard).
